# Heterophase *fcc‐hcp‐fcc* High‐Entropy Alloy Nanomaterials with Tailored Electron Divergence for Selective Ammonia Electrosynthesis

**DOI:** 10.1002/adma.202521096

**Published:** 2026-01-22

**Authors:** Xiang Meng, Mingzi Sun, Xinyi Li, Jing Chen, Yunhao Wang, Fengkun Hao, Fu Liu, Quanlin Zhao, Liang Guo, Yuecheng Xiong, Juan Wang, Guozhi Wang, Mingzheng Shao, Chaohui Wang, Xiaoqing Huang, Qinghua Zhang, Xiao Zhao, Bolong Huang, Zhanxi Fan

**Affiliations:** ^1^ Department of Chemistry City University of Hong Kong Kowloon Hong Kong SAR China; ^2^ Hong Kong Branch of National Precious Metals Material Engineering Research Center (NPMM) City University of Hong Kong Kowloon Hong Kong SAR China; ^3^ Department of Applied Biology and Chemical Technology The Hong Kong Polytechnic University Kowloon Hong Kong SAR China; ^4^ Key Laboratory of Automobile Materials of MOE School of Materials Science and Engineering Jilin University Changchun China; ^5^ Institute of Physics Beijing National Laboratory For Condensed Matter Physics Chinese Academy of Sciences Beijing China; ^6^ State Key Laboratory of Physical Chemistry of Solid Surfaces College of Chemistry and Chemical Engineering Xiamen University Xiamen China; ^7^ Hong Kong Institute for Clean Energy City University of Hong Kong Kowloon Hong Kong SAR China; ^8^ City University of Hong Kong Shenzhen Research Institute Shenzhen China

**Keywords:** ammonia, electrocatalysis, high‐entropy alloys, nitrate reduction reaction, phase engineering

## Abstract

Electrocatalytic nitrate reduction reaction provides a broad prospect for developing green electrochemical ammonia production and efficient treatment of industrial wastewater rich in nitrate, but poses a challenge to the high activity and stability of electrocatalysts. Herein, we report the versatile and scalable one‐pot solvothermal synthesis of a series of RuFeMMnMo (M═CoNi, Co, and Ni) high‐entropy alloy (HEA) nanomaterials, possessing a unique face‐centered cubic‐hexagonal close‐packed‐face‐centered cubic (*fcc‐hcp‐fcc*) heterophase. The highly random distribution of multiple metal components and the tunable diversity of metal atomic arrangements can be realized simultaneously. Significantly, RuFeCoNiMnMo HEAs present a high Faradaic efficiency of 99.3 % and a promising yield rate of 83.35 mg h^−1^ mg_cat_^−1^ toward ammonia production at −0.6 V vs. reversible hydrogen electrode. Ex/in situ characterizations and theoretical calculations have revealed that by high electron coupling of high‐entropy effect, heterophase *fcc‐hcp‐fcc* RuFeCoNiMnMo HEAs have shown strong electronic modulations with charge redistributions. The positive charges and negative charges for Ru sites and Ni/Co sites promote the adsorption of key intermediates and generation of active protons, respectively, which guarantees efficient nitrate reduction due to the reduced energy barriers.

## Graphical abstract

Heterophase *fcc‐hcp‐fcc* RuFeMMnMo (M═CoNi, Co, and Ni) high‐entropy alloy nanomaterials have been successfully synthesized using a one‐pot approach. The highly random distribution of multiple metal components and the tunable diversity of metal atomic arrangements can be realized simultaneously. By combining metals with different work functions, *fcc‐hcp‐fcc* RuFeCoNiMnMo nanoflowers present strong electronic modulations, promoting selective nitrate electroreduction to ammonia.

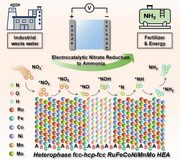

## 1 Introduction

Ammonia (NH_3_) is the core feedstock for nitrogen fertilizer production, which is of great significance for global food security and supporting human growth and development. The global demand for NH_3_ in fertilizer production was 132 million metric tons (Mt) in 2021, and it is expected to reach 165 Mt per year by 2050 [1, 2, 3]. In addition, NH_3_ has also been regarded as an important hydrogen carrier for carbon‐free energy. Its characteristics of high‐volume energy density (11.5 MJ L^−1^), net‐zero carbon emission, and easy liquefaction storage and transportation make it a supplementary scheme for hydrogen energy economy [4, 5, 6]. At present, the industrial‐scale NH_3_ synthesis is the Haber‐Bosch process, which operates under high pressures (200–350 atm) and high temperatures (400°C–600°C), and accounts for 1.44% of global carbon dioxide emission [7, 8]. Increasing attention has been paid to the electrocatalytic nitrate (NO_3_^−^) reduction reaction (NO_3_RR), using renewable electricity as driving force and NO_3_^−^ in industrial wastewater as nitrogen source. The dual role of NO_3_RR makes it a key link between environmental protection and future clean energy [9, 10, 11]. NO_3_RR is an interdependent multi‐step reaction involving multiple electron‐proton (eight electrons and nine protons) transfer processes, which puts higher requirements on the activity, selectivity, and stability of the electrocatalyst. Noble metals such as ruthenium (Ru) have been considered as the effective active site for NO_3_RR [12, 13]. However, the active sites of mono/bi‐metallic catalysts are limited, and cannot simultaneously stabilize the multiple key intermediates (e.g., *NO, *NOH, *N, *NH, *NHOH, and *NH_2_OH). By introducing various metal elements into Ru‐based nanomaterials, the heterogeneous catalytic process can be effectively regulated to break the interdependence between the adsorption, reaction, and desorption processes [14].

The high‐entropy alloy (HEA) system is a good strategy to improve the limited product selectivity and activity of electrocatalysts. HEA electrocatalysts have more multiple components (containing five or more than five uniformly mixed metal elements) and a more disordered structure, which enables the special high‐entropy effect and cocktail effect (interesting interactions among incorporated elements), and could significantly promote the NO_3_RR performance [15, 16, 17, 18, 19]. Since the concept of HEA was proposed in 2004, HEAs for electrocatalysis have received unprecedented attention. The electrocatalytic performance has been improved effectively by matching the type, quantity, and ratio of metal components, adjusting the electronic structure (e.g., defect, doping, single atoms, heterostructure, and strain), and designing the tunable bulk and surface structures [16, 20, 21, 22, 23, 24, 25]. However, it remains difficult to break the intrinsic properties of HEAs and design the active site in a targeted way. By adjusting the atomic arrangement of metals, phase engineering of metal nanomaterials can greatly change the intrinsic catalytic properties [26, 27, 28, 29, 30]. The face‐centered cubic (*fcc*) phase of metals demonstrates the characteristic stacking sequence of “ABC” along the close‐packed direction, while the hexagonal close‐packed (*hcp*) phase shows the “AB” stacking sequence [31, 32]. If the phase engineering concept is introduced into the high‐entropy system, then the highly random distribution of multiple metal components and the tunable diversity of metal atomic arrangement could be realized simultaneously [15, 33].

Reported HEA nanomaterials are synthesized mainly by non‐equilibrium methods under harsh conditions and high‐energy consumption. In this work, we report the synthesis of heterophase *fcc‐hcp‐fcc* RuFeMMnMo (M═CoNi, Co, and Ni) HEA nanoflowers via the facile one‐pot solvothermal method with versatility and scalability. Compared with RuFeNiMnMo and RuFeCoMnMo HEAs, there is a higher electron divergence between the components of RuFeCoNiMnMo, resulting in an uneven distribution of electrons, due to the co‐introduction of Co and Ni with high work functions. Ru, Fe, Mn, and Mo active sites lose electrons and show positively charged (δ^+^) properties, which are more conducive to the adsorption of NO_3_^−^ and other active intermediates. Impressively, RuFeCoNiMnMo HEAs demonstrate excellent NO_3_RR performance with the highest Faradaic efficiency (FE) of near 100% (99.9 %) at −0.3 V vs. reversible hydrogen electrode (vs. RHE), and maximum yield rate of 83.35 mg h^−1^ mg_cat_^−1^ at −0.6 V (vs. RHE) for NH_3_ electrosynthesis. In addition, the FE could maintain above 90% over a wide potential range (−0.2–−0.6 V vs. RHE). Density functional theory (DFT) calculations have indicated that the superior electroactivity of RuFeCoNiMnMo HEAs originated from the co‐existence of *hcp* and *fcc* phases as well as the electronic optimizations induced by Co sites. The surface charge redistributions in RuFeCoNiMnMo HEAs supply strong adsorption of key intermediates to facilitate the NO_3_RR with stronger reaction trends and lower energy costs.

## 2 Results and Discussion

### 2.1 Synthesis and Structural Characterization

**FIGURE 1:**
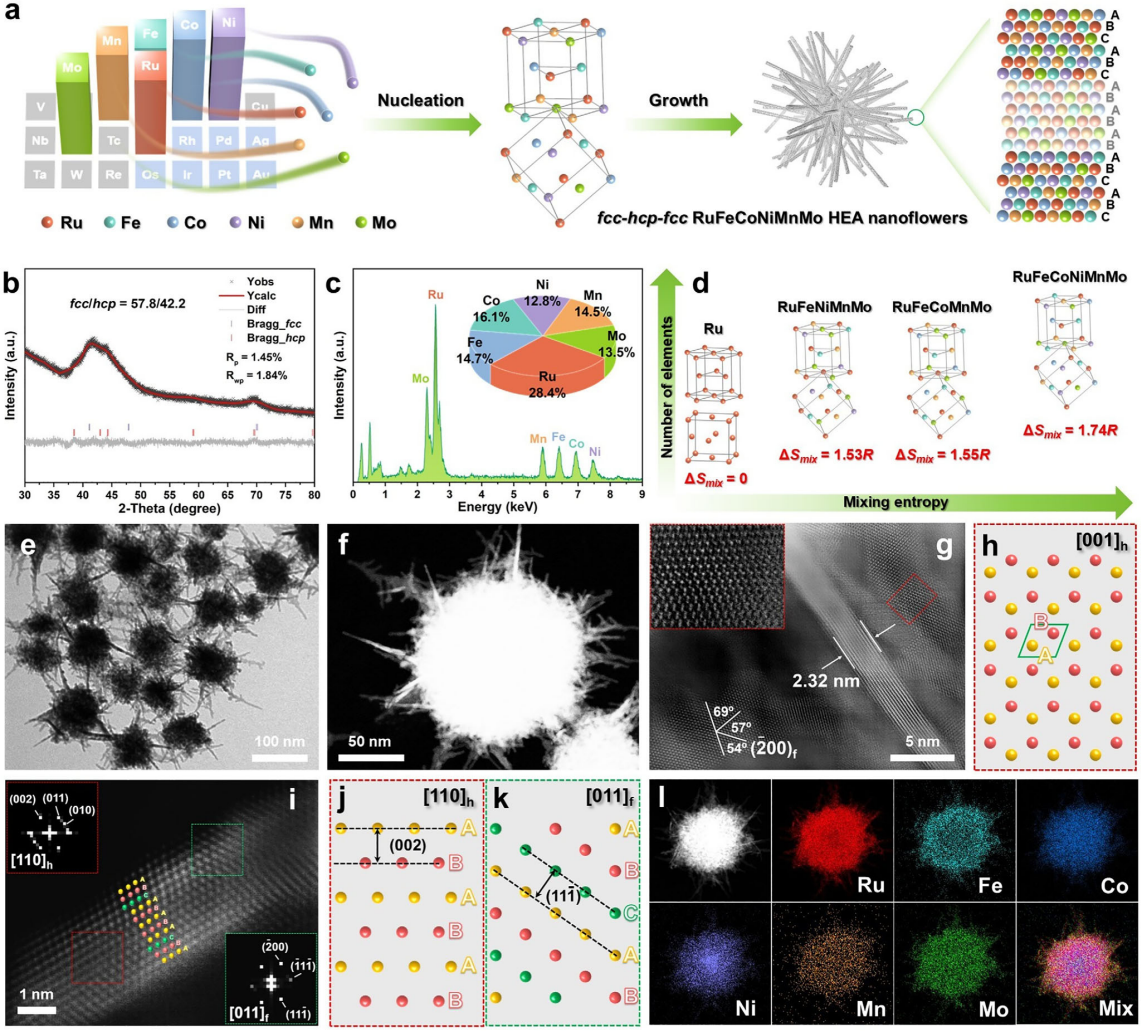
Synthesis and structural characterization. (a) Scheme of the synthesis of heterophase *fcc‐hcp‐fcc* RuFeCoNiMnMo HEA nanoflowers, containing six metals with different work functions. (b) XRD pattern of *fcc‐hcp‐fcc* RuFeCoNiMnMo HEAs with detailed Rietveld refinement. The black cross curve and red curve represent the measured pattern and fitting result. (c) SEM‐EDS spectrum of *fcc‐hcp‐fcc* RuFeCoNiMnMo HEAs. Inset: The pie chart shows the proportion of every component. (d) Conceptional illustration of the high‐entropy effect about mixing entropy and number of elements from pure *fcc* or *hcp* Ru to *fcc‐hcp‐fcc* RuFeMMnMo (M═CoNi, Co, and Ni) HEAs. (e–g) TEM (e), HAADF‐STEM (f), and atomic resolution HAADF‐STEM (g) images of *fcc‐hcp‐fcc* RuFeCoNiMnMo HEAs. Inset of (g): the enlarged image of the red dashed rectangle‐marked region in (g). (h) Atomic arrangement model of the *hcp* phase viewed along the [001]_h_ direction. (i) Atomic resolution HAADF‐STEM image of *fcc‐hcp‐fcc* RuFeCoNiMnMo HEAs. Insets of (i): the corresponding fast Fourier transform (FFT) patterns of the red (*hcp*) and green (*fcc*) dashed squares marked regions in (i). (j) Atomic arrangement model of the *hcp* phase viewed along the [110]_h_ direction. (k) Atomic arrangement model of the *fcc* phase viewed along the [011]_f_ direction. (l) HAADF‐STEM image and the corresponding elemental mappings of *fcc‐hcp‐fcc* RuFeCoNiMnMo HEAs.

A series of heterophase *fcc‐hcp‐fcc* HEA nanoflowers were synthesized via a scalable one‐pot solvothermal method. For the typical synthesis of RuFeCoNiMnMo HEA, triruthenium dodecacarbonyl (Ru_3_(CO)_12_), iron(III) acetylacetonate (Fe(acac)_3_), cobalt(II) acetylacetonate (Co(acac)_2_), nickel(II) acetylacetonate (Ni(acac)_2_), manganese carbonyl (Mn_2_(CO)_10_), and molybdenum hexacarbonyl (Mo(CO)_6_) were used as the metal precursors. Mo(CO)_6_ also acted as the reducing agent, and oleylamine was the solvent, as schematically illustrated in Figure 1a (Supporting Information for more details). The crystalline structure of as‐synthesized heterophase *fcc‐hcp‐fcc* RuFeCoNiMnMo HEAs was investigated by X‐ray diffraction (XRD). From Figure 1b, two shoulder peaks appeared at 38.5° and 44.0°, which match well with the *hcp* phase (JCPDS No. 06–0663), while a wide main peak at 41.4° appears between the reference peak of *hcp*‐(002) and *fcc*‐(111), suggesting the co‐existence of *hcp* and *fcc* phases (JCPDS No. 88–2333) [34]. No obvious characteristic peaks could be assigned to the diffraction from metallic Fe, Co, Ni, Mn, Mo, and relevant oxides [14]. Rietveld refinement analysis demonstrates that the ratio of the *fcc* and *hcp* phase is 57.8/42.2. The weak and wide diffraction peaks in XRD pattern could be attributed to the ultrathin nanostructure and lattice distortion induced by the atomic size differences of multiple elements [35]. Scanning electron microscopy energy dispersive X‐ray spectroscopy (SEM‐EDS) measurement shows that the atomic ratio of Ru/Fe/Co/Ni/Mn/Mo is 28.4/14.7/16.1/12.8/14.5/13.5 (Figure 1c). Additionally, three prerequisites for the formation of HEA system were calculated: (1) the mixing entropy (Δ*S_mix_*) is 1.74*R*; (2) the mixing enthalpy (Δ*H*_mix_) is −7.0 kJ mol^−1^; (3) the atomic radius difference (*δ*) is 4.4% (Note S1 and Tables S1–S3). Above calculation results prove that the as‐synthesized heterophase *fcc‐hcp‐fcc* RuFeCoNiMnMo alloys meet the prerequisites for forming HEA and there is no elemental segregation [15, 36, 37]. Impressively, the Δ*S_mix_* of RuFeCoNiMnMo is higher than that of RuFeNiMnMo (1.53*R*) and RuFeCoMnMo (1.55*R*), respectively, indicating greater atomic disorder, which is conducive to activating more active sites (Figure 1d).

Transmission electron microscopy (TEM), high‐resolution TEM (HRTEM), and high‐angle annular dark‐field scanning TEM (HAADF‐STEM) characterizations show that heterophase *fcc‐hcp‐fcc* RuFeCoNiMnMo HEAs exist as 3D nanoflower structure (Figure 1e,f; Figure S1), assembled by ultrathin nanodendrites with a thickness of about 2 nm (Figure 1g; Figure S2). The crystal structure of heterophase *fcc‐hcp‐fcc* RuFeCoNiMnMo HEAs was investigated by the atomic resolution HAADF‐STEM. The clear atomic arrangement resembling a hexatomic ring fits the typical *hcp* phase viewed along the [001]_h_ direction (Figure 1g), as shown in the atomic model in Figure 1h [38, 39]. The atomic arrangement in the bottom‐left corner of Figure 1g corresponds to the *fcc* phase along the [011]_f_ zone axis, but the angle of crystal facets is slightly different from that of standard map, which should be attributed to the defect caused by high‐entropy effect and the difference in atomic sizes. Importantly, from Figure 1i, the middle region of crystals shows the typical atomic stacking sequences of “AB” along the close‐packed [001]_h_ direction, and the two ends show the atomic stacking sequences of “ABC” along the close‐packed [11$\bar{1}$]_f_ direction, proving the existence of *fcc‐hcp‐fcc* heterophase [40, 41]. The lattice spacings are measured to be 0.21 and 0.22 nm, corresponding to the (002)_h_ and (11$\bar{1}$)_f_ facets, respectively, as shown by the atomic model of *hcp* and *fcc* phases (Figure 1j,k) [38]. TEM energy dispersive X‐ray spectroscopy (TEM‐EDX) elemental mappings show the homogeneous distribution of Ru, Fe, Co, Ni, Mn, and Mo in a single RuFeCoNiMnMo nanoflower (Figure 1l).

By regulating the reaction conditions, it was found that the reaction time and dosages of Mo(CO)_6_ play an important role in the synthesis of heterophase *fcc‐hcp‐fcc* RuFeCoNiMnMo HEAs (Figures S3–S8). At the reaction time of 3 h, only nanoparticles were obtained, and the atomic ratio of Ni occupies 50%, which indicates that Ni is easier to be reduced (Figures S3a,b and S4a). The XRD pattern matches well with typical Ni (JCPDS No. 04–0850, Figure S5) [42]. When the reaction time was increased from 3 to 6 h, nanoflowers assembled by nanodendrites were observed, but the assembly degree is relatively low (Figure S3c,d). The atomic ratio of Ni is still ca. 50%, indicating that this nanoflower is Ni‐rich (Figure S4b). When the reaction time was increased to 24 h, the flower‐like structure could be maintained stably (Figure S3e,f). For the dosages of Mo(CO)_6_, with the increase of dosages from 11 to 44 mg, the morphology changes a little (Figure S6), but the diffraction peak of *fcc* phase becomes stronger (Figure S8). In addition, it is worth noting that the reaction temperature (Figures S9–S11), concentration of metal precursors (Figure S12), and dosages of Ru_3_(CO)_12_ (Figure S13) also have significant effects on the synthesis of *fcc‐hcp‐fcc* HEA nanoflowers.

In order to explore the interaction of multiple active sites, heterophase *fcc‐hcp‐fcc* RuFeNiMnMo and RuFeCoMnMo HEAs were synthesized as control samples by a similar method, respectively (Supporting Information for more details). TEM and HAADF‐STEM characterizations show that the obtained RuFeNiMnMo and RuFeCoMnMo HEAs exist as nanoflower structures assembled by nanodendrites, similar to that of RuFeCoNiMnMo HEAs (Figures S14 and S16). In addition, the atomic arrangement of RuFeNiMnMo and RuFeCoMnMo HEAs also adopts the *fcc‐hcp‐fcc* heterophase, and the elemental mappings verify the uniform distribution of elements (Figures S15 and S17). The heterophase of RuFeNiMnMo and RuFeCoMnMo was further confirmed by the XRD patterns (Figure S18). Besides, using a similar method, a series of quinary alloys (RuMoCoNiX, X═Fe, Mn), quarternary alloys (RuMoXY, XY═FeCo, FeNi, FeMn, and CoNi), and ternary alloys (RuMoX, X═Fe, Mn) have also been synthesized (Figures S20–S23).

### 2.2 X‐ray Spectral Analysis

**FIGURE 2:**
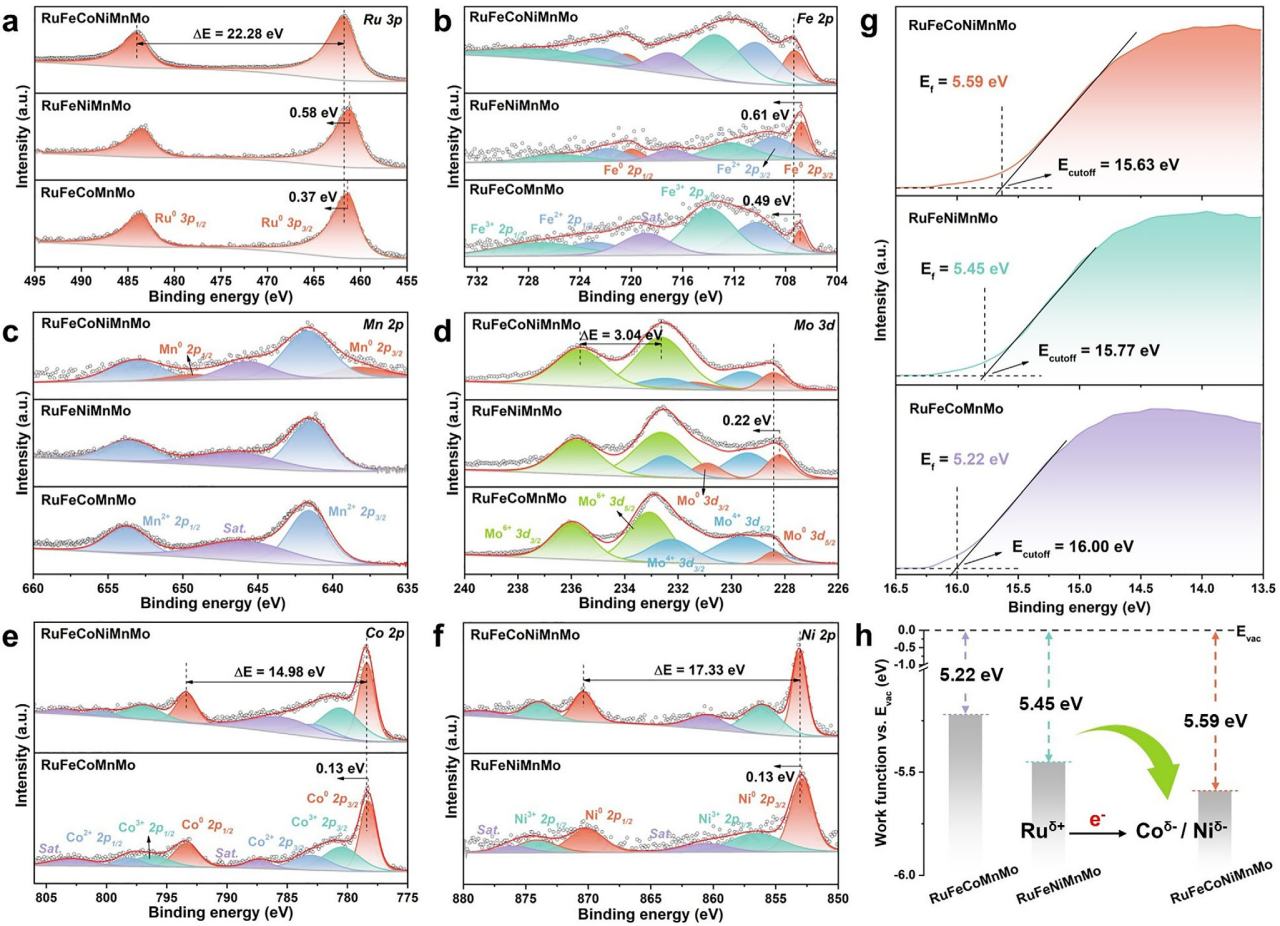
Chemical state and work function characterizations. (a–f) High‐resolution XPS spectra of *fcc‐hcp‐fcc* RuFeCoNiMnMo, RuFeNiMnMo, and RuFeCoMnMo HEAs for (a) Ru 3p, (b) Fe 2p, (c) Mn 2p, (d) Mo 3d, (e) Co 2p, and (f) Ni 2p. (g,h) UPS spectra (g) and work functions (h) of *fcc‐hcp‐fcc* RuFeCoNiMnMo, RuFeNiMnMo, and RuFeCoMnMo HEAs.

X‐ray photoelectron spectroscopy (XPS) was used to investigate the surface compositions and electron divergence effect of the heterophase *fcc‐hcp‐fcc* RuFeCoNiMnMo, RuFeNiMnMo, and RuFeCoMnMo HEAs (Figure 2; Figure S24). The high‐resolution Ru 3p XPS spectrum of RuFeCoNiMnMo shows that only the metallic Ru state exists, and the peaks located at 461.76 and 484.04 eV are assigned to the Ru^0^ 3p_3/2_ and Ru^0^ 3p_1/2_, respectively [43]. More importantly, the Ru^0^ 3p_3/2_ position of RuFeCoNiMnMo positively shifted by about 0.58 and 0.37 eV compared with that of RuFeNiMnMo and RuFeCoMnMo, respectively (Figure 2a). In the deconvoluted Fe 2p spectrum of RuFeCoNiMnMo, six peaks located at 707.36, 710.34, 713.40, 720.34, 722.17, and 726.61 eV are attributed to Fe^0^ 3p_3/2_, Fe^2+^ 3p_3/2_, Fe^3+^ 3p_3/2_, Fe^0^ 3p_1/2_, Fe^2+^ 3p_1/2_, and Fe^3+^ 3p_1/2_, respectively (Figure 2b) [14]. The peak at 716.97 eV could match with satellite feature of Fe 2p. Similar to Ru 3p, the Fe^0^ 3p_3/2_ position of RuFeCoNiMnMo has a positive shift of 0.61 and 0.49 eV compared with that of RuFeNiMnMo and RuFeCoMnMo, respectively. The Mn 2p spectrum of RuFeCoNiMnMo displays the co‐existence of metallic Mn^0^ (638.28 and 649.35 eV for Mn^0^ 2p_3/2_ and Mn^0^ 2p_1/2_) and Mn^2+^ (641.67 and 653.14 eV for Mn^2+^ 2p_3/2_ and Mn^2+^ 2p_1/2_), and the peak located at 645.75 eV corresponds to the satellite feature (Figure 2c). For the Mo 3d spectrum of RuFeCoNiMnMo, the main doublets at 228.42, 231.43, 229.53, 232.44, 232.63, and 235.67 eV are ascribed to Mo^0^ 3d_5/2_, Mo^0^ 3d_3/2_, Mo^4+^ 3d_5/2_, Mo^4+^ 3d_3/2_, Mo^6+^ 3d_5/2_, and Mo^6+^ 3d_3/2_, respectively. Similarly, The Mo^0^ 3d_5/2_ peak of RuFeCoNiMnMo positively shifted by 0.22 eV compared with that of RuFeNiMnMo (Figure 2d). The relative shift of Ru 3p, Fe 2p, and Mo 3d spectra proves the high electron divergence effect of multiple active sites in the HEA system [44]. Particularly, Ru, Fe, and Mo sites could act as the electron donors and transfer electrons to Co or Ni sites. However, for Co 2p orbital, the main peak (778.40 eV for Co^0^ 2p_3/2_) shift is only about 0.13 eV (within the fitting error range, Figure 2e), when comparing RuFeCoMnMo and RuFeCoNiMnMo (before and after adding Ni). Similar to Co 2p orbital, the Ni^0^ 2p_3/2_ position shows a slight shift after adding Co element into this HEA system (Figure 2f). The O 1s spectrum of RuFeCoNiMnMo reveals that the peak intensity of O‐lattice located at 529.92 eV for metal oxides is much weaker than that of O‐adsorbed (531.26 eV) and residual O‐containing groups (533.27 eV) on the surface (Figure S25). The detailed deconvoluted XPS analysis was summarized in Table S4. To clarify the electron state of catalyst surfaces, ultraviolet photoelectron spectroscopy (UPS) was also used to obtain the work functions (W_f_). The W_f_ difference will drive the highly spontaneous electron transfer on the catalyst surface [45]. The W_f_ of RuFeCoNiMnMo is 5.59 eV, which is higher than that of RuFeNiMnMo and RuFeCoMnMo (5.45 and 5.22 eV, respectively) (Figure 2g). The results show that electrons will transfer from the Ru, Fe, Mn, and Mo with lower W_f_ to Co and Ni with higher W_f_ (Figure 2h). Note that a more positively charged Ru active site makes it easier to adsorb NO_3_^−^ on the substrate [13].

**FIGURE 3:**
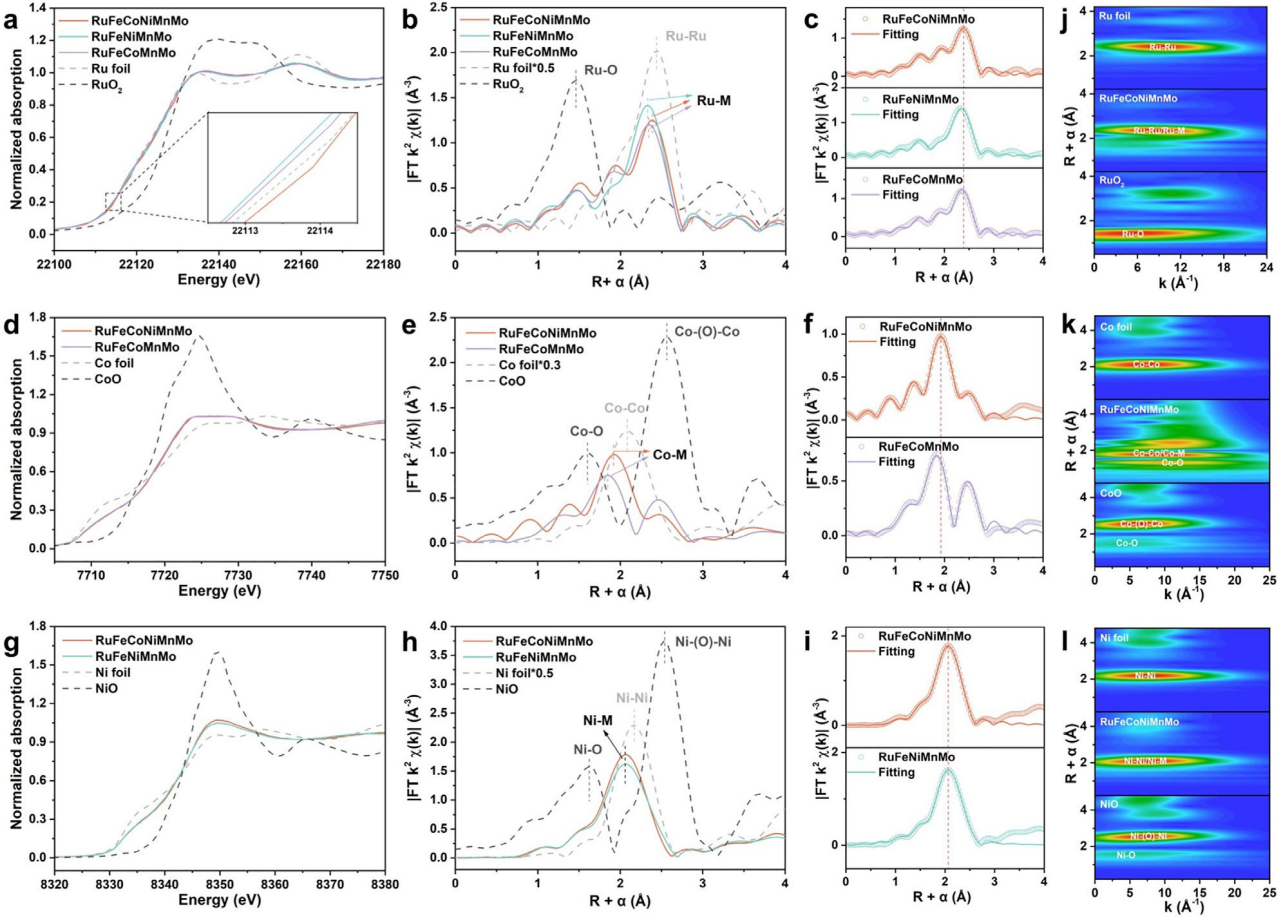
Electronic structure and coordination environment analysis. (a–c) Ru *K*‐edge XANES spectra (a), *k^2^*‐weighted FT‐EXAFS (b), and EXAFS fitting results (c) of *fcc‐hcp‐fcc* RuFeCoNiMnMo, RuFeNiMnMo, and RuFeCoMnMo HEAs in reference to Ru foil and RuO_2_. (d–f) Co *K*‐edge XANES spectra (d), *k^2^*‐weighted FT‐EXAFS (e), and EXAFS fitting results (f) of *fcc‐hcp‐fcc* RuFeCoNiMnMo and RuFeCoMnMo HEAs in reference to Co foil and CoO. (g–i) Ni *K*‐edge XANES spectra (g), *k^2^*‐weighted FT‐EXAFS (h), and EXAFS fitting results (i) of *fcc‐hcp‐fcc* RuFeCoNiMnMo and RuFeNiMnMo HEAs in reference to Ni foil and NiO. (j) WT‐EXAFS spectra at Ru *K*‐edge of *fcc‐hcp‐fcc* RuFeCoNiMnMo HEAs, Ru foil and RuO_2_, respectively. (k) WT‐EXAFS spectra at Co *K*‐edge of *fcc‐hcp‐fcc* RuFeCoNiMnMo HEAs, Co foil and CoO, respectively. (l) WT‐EXAFS spectra at Ni *K*‐edge of *fcc‐hcp‐fcc* RuFeCoNiMnMo HEAs, Ni foil and NiO, respectively.

The X‐ray absorption spectroscopy was further performed to investigate the electronic structures and local coordination environments (Figure 3). The X‐ray absorption near‐edge structure (XANES) spectra show that the Ru *K*‐edge of RuFeCoNiMnMo, RuFeNiMnMo, and RuFeCoMnMo HEAs are close to that of Ru foil, but away from RuO_2_, suggesting Ru mainly adopts the metallic state (Figure 3a) [46]. The post‐edges of all three samples show deviations in peak intensities and shapes corresponding to the reference foils and oxides, suggesting the orbital hybridization of multiple elements. Importantly, the rising edge for RuFeCoNiMnMo is located at a higher energy than that for both RuFeNiMnMo and RuFeCoMnMo, indicating the more electron loss of Ru site in RuFeCoNiMnMo [47], which is consistent with XPS results (Figures 3a and 2a). In the extended X‐ray absorption fine structure (EXAFS) spectra at Ru *K*‐edge, one dominant peak located at around 2.43 Å for Ru foil is assigned to the Ru─Ru scattering path (Figure 3b) [48]. However, the averaged Ru─M paths of RuFeCoNiMnMo, RuFeNiMnMo, and RuFeCoMnMo HEAs scatter at smaller radial distances than that for the Ru foil, suggesting the Ru atoms are surrounded by different metallic species to form the alloys. The fitting results show that the corresponding coordination numbers (C.N.) of Ru─Ru for RuFeCoNiMnMo, RuFeNiMnMo, and RuFeCoMnMo HEAs are 3.9, 3.8, and 3.8, respectively (Figure 3c; Figure S26, and Table S5). In the Co *K*‐edge XANES spectra, the white line intensities of RuFeCoNiMnMo and RuFeCoMnMo HEAs are higher than that of Co foil but much lower than that of CoO, suggesting the mainly metallic state of Co but with slight oxidation on the surface for two samples (Figure 3d). EXAFS spectra at Co *K*‐edge show that the averaged Co─M paths for RuFeCoNiMnMo and RuFeCoMnMo HEAs scatter quite differently from the Co─Co one of the Co foil, which proves that the Co atoms in HEAs have significantly different coordination environments from the Co one in Co foil (Figure 3e). In addition, the averaged Co─M path shifts to a longer radial distance from ∼ 1.85 Å of RuFeCoMnMo to ∼ 1.93 Å of RuFeCoNiMnMo, suggesting a more disordered coordination environment due to the introduction of Ni atoms (Figure 3e,f; Figure S27). The Ni *K*‐edge XANES and EXAFS spectra prove the Ni species for RuFeCoNiMnMo and RuFeNiMnMo HEAs are mainly in metallic states when compared with the reference Ni foil and NiO (Figure 3g–i; Figure S28). It is worth noting that all EXAFS spectra at Ru‐, Co‐, and Ni‐edges for RuFeCoNiMnMo, RuFeNiMnMo, and RuFeCoMnMo HEAs demonstrate the much lower scattering amplitudes in comparison with the corresponding foils, which are due to the defects and surface unsaturated coordination of the high‐entropy system (Figure 3b,e,h) [49]. The best‐fitting FT‐EXAFS results are summarized in Tables S5–S7. Then, the wavelet transformed (WT) of Ru, Co, and Ni EXAFS oscillation was further employed to analyze the valence state and coordination environment of RuFeCoNiMnMo HEAs. The intensity maxima at Ru, Co, Ni *K*‐edge of RuFeCoNiMnMo all show the down‐shifts at the R spaces, as compared to their foils due to the alloying effect (Figure 3j–l) [50].

### 2.3 Electrochemical NO_3_RR Performances

**FIGURE 4:**
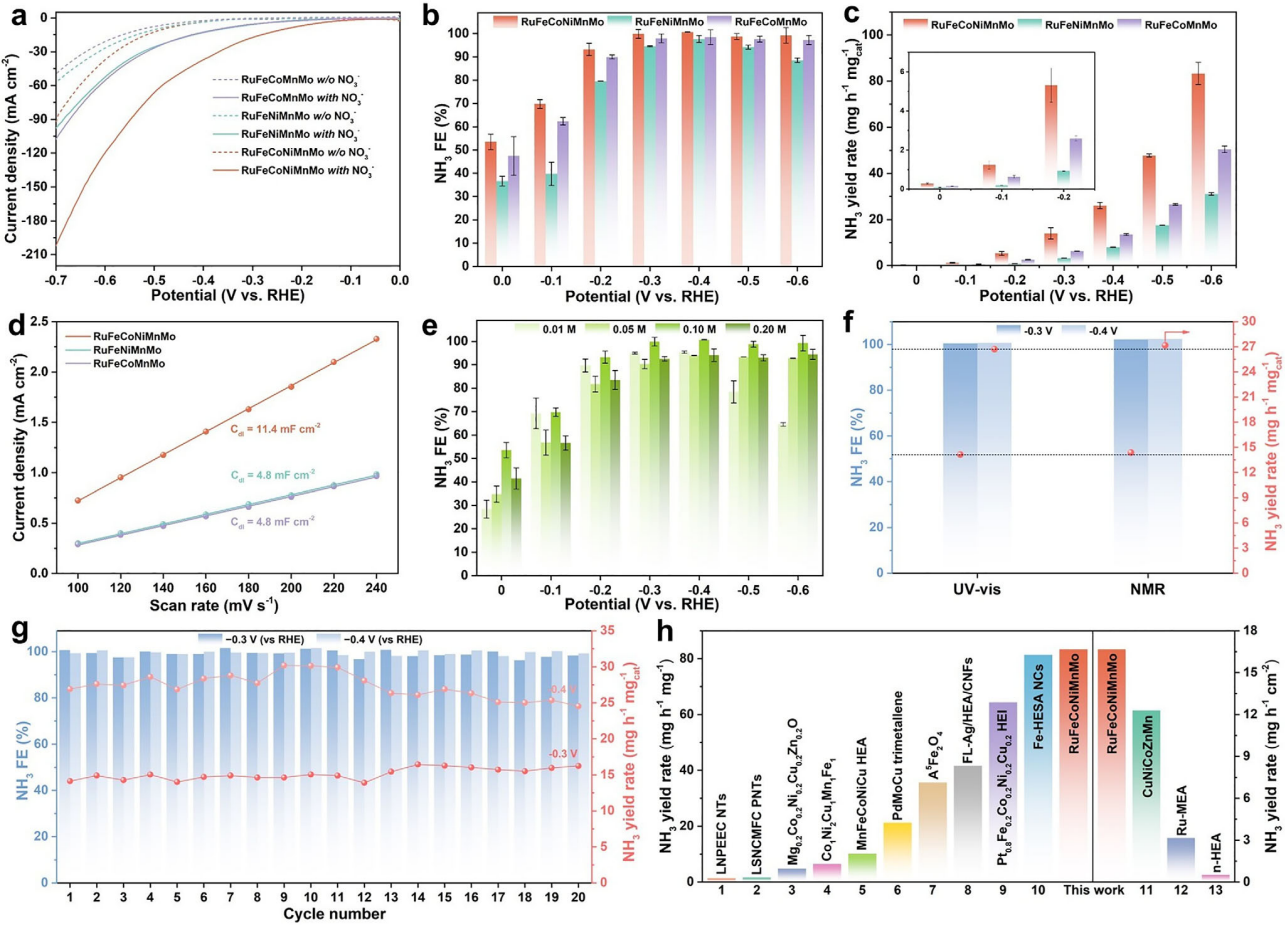
Electrocatalytic performance evaluation. (a) LSV curves of *fcc‐hcp‐fcc* RuFeCoNiMnMo, RuFeNiMnMo, and RuFeCoMnMo HEAs in 0.50 m K_2_SO_4_ with or without (*w/o*) 0.10 m KNO_3_. (b–d) NH_3_ FE (b), NH_3_ yield rate (c) and fitting results of C_dl_ (d) of *fcc‐hcp‐fcc* RuFeCoNiMnMo, RuFeNiMnMo, and RuFeCoMnMo HEAs. (e) NH_3_ FE of *fcc‐hcp‐fcc* RuFeCoNiMnMo HEAs with different nitrate concentrations. (f) Comparison of the NH_3_ FE and NH_3_ yield rate obtained by colorimetric UV–Vis and NMR methods at −0.3 and −0.4 V (vs. RHE). (g) The consecutive recycling electrolysis test of *fcc‐hcp‐fcc* RuFeCoNiMnMo HEAs at −0.3 and −0.4 V (vs. RHE). (h) Comparative analysis of NH_3_ yield rate between this work and previously reported high‐entropy/multicomponent electrocatalysts.

The electrochemical NO_3_RR performance of a series of as‐prepared heterophase *fcc‐hcp‐fcc* HEA nanoflowers was evaluated by a standard three‐electrode H‐type cell operating in neutral electrolyte containing 0.50 m K_2_SO_4_ and 0.10 m KNO_3_. The linear sweep voltammetry (LSV) curves show that all cases have an increased current density in the presence of NO_3_^−^, which proves that these *fcc‐hcp‐fcc* HEA nanoflowers have a good potential for electrochemical NO_3_RR (Figure 4a). In addition, the current density of RuFeCoNiMnMo HEAs is significantly higher than that of RuFeNiMnMo and RuFeCoMnMo, respectively, suggesting the better NO_3_RR performance of RuFeCoNiMnMo [51]. Chronoamperometry method was then used to test the NO_3_RR activity in the whole potential range from 0 to −0.6 V (vs. RHE, Figure S29). All the possible products including NH_3_ and NO_2_^−^ are determined by the colorimetric method, and quantified by comparing with standard calibration curves (Figures S30 and S31). Figure 4b shows the NH_3_ FE from 0 to −0.6 V (vs. RHE). RuFeCoNiMnMo HEAs demonstrate a promising NH_3_ FE of 99.9% at −0.3 V (vs. RHE), and FE above 90% over a wide potential range of −0.2–−0.6 V (vs. RHE), which proves that RuFeCoNiMnMo HEAs can effectively inhibit the competitive hydrogen evolution reaction (HER). In addition, the NH_3_ FE of RuFeCoNiMnMo is higher than that of RuFeNiMnMo and RuFeCoMnMo at all the tested potentials. Besides, the NH_3_ yield rate of RuFeCoNiMnMo is also higher than the other two HEAs, and gradually increases as the applied potential becomes more negative (Figure 4c). The yield rate in the tested potential range is in the order of RuFeCoNiMnMo (83.35 mg h^−1^ mg_cat_^−1^ at −0.6 V (vs. RHE)) > RuFeCoMnMo (50.48 mg h^−1^ mg_cat_^−1^ at −0.6 V (vs. RHE)) > RuFeNiMnMo (31.09 mg h^−1^ mg_cat_^−1^ at −0.6 V (vs. RHE)). The highest NH_3_ yield rate of RuFeCoNiMnMo is approximately 2.7 and 1.7 times that of RuFeNiMnMo and RuFeCoMnMo, respectively. As for NO_2_^−^, the main by‐product during NO_3_RR, all HEA samples demonstrate extremely low NO_2_^−^ FE (Figure S32). To the best of our knowledge, while maintaining the near 100% FE, RuFeCoNiMnMo HEAs demonstrate much higher NH_3_ yield rate compared with most of the previously reported high‐entropy/multi‐component or Ru‐containing electrocatalysts (Figure 4h, Tables S9 and S10).

Moreover, the quantitative analysis of NH_3_ FE and yield rate was also performed by the nuclear magnetic resonance (NMR) method using maleic acid as the external standard (Figure S33). Figure 4f shows that under two different potentials, −0.3 and −0.4 V (vs. RHE), the NH_3_ FE and yield rate measured by colorimetric and NMR methods are quite close to each other, indicating the accuracy of relevant results. The isotope labeling experiment was further performed to verify the origin of produced NH_3_ (Figure S34) [52, 53]. The NMR spectra prove that the NH_3_ detected is entirely produced by the feeding nitrate instead of the other kinds of N‐sources.

According to the above experiments, the NO_3_RR performance of RuFeCoNiMnMo is significantly better than that of RuFeNiMnMo and RuFeCoMnMo, respectively. To find out the reason for the improved NO_3_RR activity, the electrochemical active surface area (ECSA) was measured via electrochemical double‐layer capacitance (C_dl_) method (Figure S35) [54]. As shown in Figure 4d, the ECSA of RuFeCoNiMnMo (285 cm_ECSA_^−2^) is higher than that of RuFeNiMnMo and RuFeCoMnMo (120 cm_ECSA_^−2^), proving that RuFeCoNiMnMo has more active sites exposed on the surface. The more active sites could be attributed to the high electron divergence effect in HEA systems. Due to the introduction of Co and Ni with larger work functions, RuFeCoNiMnMo has the uneven distribution of electrons among various metal elements (Figure 2h), which causes the Ru site to become positively charged (δ^+^), promoting the adsorption and activation of nitrate/intermediates [55]. In addition, this kind of nanoflower structure self‐assembled by nano‐dendrites could provide abundant active sites for the electrocatalytic reactions.

To explore the applicability for different industrial scenarios, the NO_3_RR performance of RuFeCoNiMnMo HEAs toward various nitrate concentrations was investigated (Figures S36–S38). On the one hand, when the nitrate concentration increases from 0.10 to 0.20 m, the NH_3_ FE maintain above 90% over a wide potential range (−0.3 ∼ −0.6 V (vs. RHE)) and the NH_3_ yield rate could reach 73.49 mg h^−1^ mg_cat_^−1^ at −0.6 V (vs. RHE), which satisfies the conditions of high concentration of electrolytes and high potential in actual industrial production (Figure 4e; Figure S37). On the other hand, when the nitrate concentration decreases from 0.10 to 0.01 m, the highest NH_3_ FE could still reach 95.4% at −0.4 V (vs. RHE), together with NH_3_ yield rate of 17.03 mg h^−1^ mg_cat_^−1^ at −0.6 V (vs. RHE), indicating the promising NO_3_RR performance even at low nitrate concentration. Note that the FE at 0.01 m nitrate shows a volcano‐trend with the decrease of potentials, which could be attributed to the slow reaction kinetics at low potentials, as well as the strong competition of HER at more negative potentials.

The catalytic stability of RuFeCoNiMnMo HEAs was evaluated by the consecutive recycling electrolysis (Figure 4g; Figures S39–S41). Under two different potentials, −0.3 and −0.4 V (vs. RHE), the NH_3_ FE could maintain near 100% and the NH_3_ yield rate also remains stable during 20 cycles, which suggests the good catalytic stability of RuFeCoNiMnMo HEAs. Particularly, after electrolysis tests, their morphology and crystal phase can be well maintained (Figure S42). In addition, the long‐term chronoamperometry test of RuFeCoNiMnMo was conducted at −0.3 V (vs. RHE) for 10 h. With the increase of electrolysis time, the current density gradually decreased because of the depletion of nitrate. But after refreshing the electrolyte, the current density could be restored to its initial level (Figure S43). The LSV curve of the first 10 h electrolysis is basically similar to that of the second 10 h electrolysis process, which proves the high catalytic durability (Figure S44). Then, the concentration of metal ions dissolved in the electrolyte after the 10 h electrolysis was evaluated by the inductively coupled plasma optical emission spectroscopy (ICP‐OES). The result shows that the dissolution of all elements presents a negligible level, indicating that the catalysts loaded on the carbon paper could maintain stability during the long‐term durability test (Table S8).

### 2.4 Catalytic Mechanism Exploration

**FIGURE 5:**
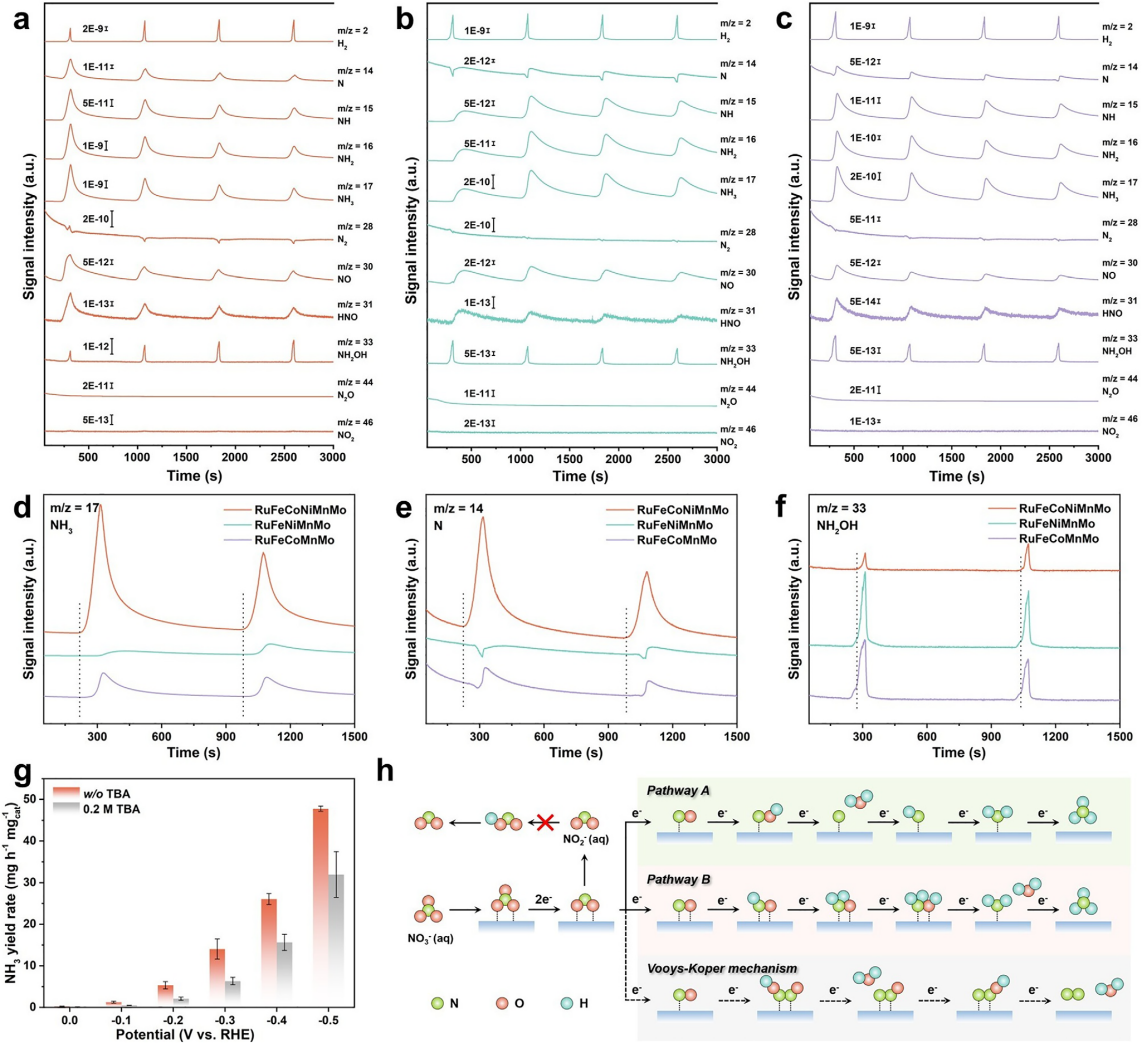
Catalytic mechanism study. (a–c) In situ DEMS spectra of *fcc‐hcp‐fcc* (a) RuFeCoNiMnMo, (b) RuFeNiMnMo, and (c) RuFeCoMnMo HEAs. (d–f) In situ DEMS spectra of (d) NH_3_, (e) N, and (f) NH_2_OH species for *fcc‐hcp‐fcc* RuFeCoNiMnMo, RuFeNiMnMo, and RuFeCoMnMo HEAs. (g) NH_3_ yield rate of *fcc‐hcp‐fcc* RuFeCoNiMnMo HEAs without and with 0.2 m TBA in electrolyte. (h) Schematic illustration for the possible reaction pathways of nitrate electroreduction.

In situ differential electrochemical mass spectrometry (DEMS) was applied to detect the unstable intermediates and products in order to study the NO_3_RR catalytic mechanism (Figure 5) [12]. Four LSV cycles (0.1–−0.7 V (vs. RHE)) with recorded mass/charge (m/z) ratio signals were performed (Figure S46). The m/z signals of 2, 14, 15, 16, 17, 28, 30, 31, 33, 44, and 46 corresponding to H_2_, N, NH, NH_2_, NH_3_, N_2_, NO, HNO, NH_2_OH, N_2_O, and NO_2_, respectively, were observed, which could reveal the potential reaction pathway (Figure 5a–c) [56]. The signal intensity of NH_3_ for RuFeCoNiMnMo HEAs is much stronger than that of RuFeNiMnMo and RuFeConMo HEAs, which corresponds to a higher NH_3_ yield rate (Figure 5d). Importantly, for the N (m/z = 14) and NH_2_OH (m/z = 33) signals, RuFeCoNiMnMo HEAs demonstrate the stronger signal of N but the weaker signal of NH_2_OH compared to RuFeNiMnMo and RuFeConMo HEAs (Figure 5e,f). According to the reported literatures, the N─O bond of *NO_3_ is continuously broken during NO_3_RR, and thus *NO_2_ and *NO intermediates are gradually generated [57, 58]. Then, *NO is a vital intermediate, whose catalytic behavior leads to the different reaction pathways (Figure 5h) [59]. If *NO is O‐end adsorbed, *NO will be reduced to *NOH, which leads to the pathway A: *NOH→*N→*NH→*NH_2_→*NH_3_ [60]. If *NO is parallelly adsorbed, the proton tends to bind with N to form *NHO, which follows the reaction pathway B: *NHO→*NH_2_O→*NH_2_OH→*NH_2_→*NH_3_ [61]. The above experimental results suggest that RuFeCoNiMnMo HEAs mainly promote the pathway A, while the two control samples facilitate the pathway B. It seems that the different reaction pathways determine the difference in NO_3_RR performance. The by‐product N_2_ (m/z = 28) is almost undetectable compared to NH_3_ (m/z = 17) at the same or lower order of magnitude, due to the high NH_3_ FE of as‐synthesized HEAs, which indicates that the Vooys‐Koper mechanism is almost completely suppressed (Figure 5h) [57]. In addition, the strong signal of H_2_ (m/z = 2) reveals the high ability to supply sufficient active hydrogen (*H) for the continuous hydrogenation. Therefore, when tert‐butyl alcohol (TBA) was introduced into the electrolyte to capture *H [56, 62, 63], there was a significant decrease in NH_3_ FE and yield rate, suggesting the vital role of *H in NO_3_RR (Figure 5g; Figures S47 and S48).

### 2.5 Theoretical Calculations

**FIGURE 6:**
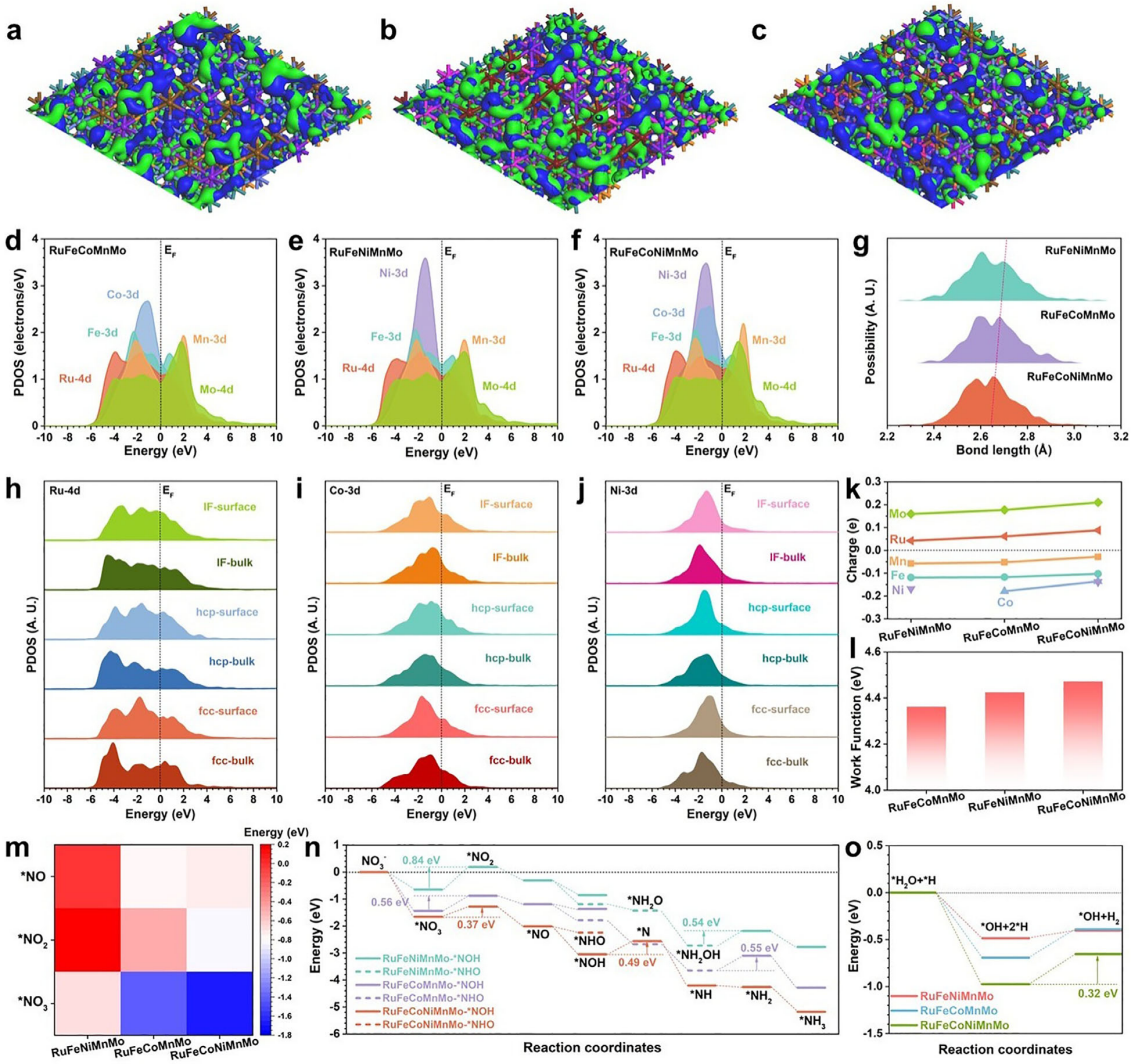
Theoretical calculations. (a–c) The electronic distributions of bonding and anti‐bonding orbitals near the Fermi level of *fcc‐hcp‐fcc* (a) RuFeCoMnMo, (b) RuFeNiMnMo, and (c) RuFeCoNiMnMo HEAs. Green balls = Ru, purple balls = Fe, light blue balls = Co, pink balls = Ni, brown balls = Mn, and orange balls = Mo. Blue isosurface = bonding orbitals, and green isosurface = anti‐bonding orbitals. (d–f) The PDOS of *fcc‐hcp‐fcc* (d) RuFeCoMnMo, (e) RuFeNiMnMo, and (f) RuFeCoNiMnMo HEAs. (g) The average bond length distributions of *fcc‐hcp‐fcc* RuFeCoMnMo, RuFeNiMnMo, and RuFeCoNiMnMo HEAs. (h–j) The site‐dependent PDOS of (h) Ru‐4d, (i) Co‐3d, and (j) Ni‐3d in *fcc‐hcp‐fcc* RuFeCoNiMnMo HEAs. (k) The charge distribution comparison. (l) The work function comparison. (m) The binding energy comparison. (n) The reaction energy changes of NO_3_RR. (o) The reaction energy changes for HER.

To investigate the superior performance of RuFeCoNiMnMo HEAs for NO_3_RR, theoretical explorations through DFT calculations were performed in this work, focusing on the electronic modulations induced by the HEAs of different metals. For the surface of RuFeCoMnMo, it is noted that the bonding orbitals are mainly distributed near the Ru and Co sites, while the Fe, Mn, and Mo only show very limited contributions to the electronic distributions near the Fermi level (E_F_) (Figure 6a). This indicates that the Ru and Co sites play as the main active sites for electron transfer and exchange during the NO_3_RR. Compared to RuFeCoMnMo, the surface of RuFeNiMnMo becomes slightly less electron‐rich due to the absence of Co and the introduction of Ni (Figure 6b). The distributions of bonding orbitals on Ni sites are slightly weakened, revealing that Ni sites are less electroactive in promoting electrocatalysis. Accordingly, the surface of RuFeCoNiMnMo is most electron‐rich, where the bonding orbitals become more dominant to supply electroactive surfaces, especially near Ru, Co, and Ni sites (Figure 6c). To have an in‐depth understanding of the electronic structures, the projected partial density of states (PDOS) of RuFeCoMnMo, RuFeNiMnMo, and RuFeCoNiMnMo are demonstrated. For all HEA structures, although the strong orbital overlapping is noted for d orbitals from different metals, the detailed electronic structures are still modulated. In RuFeCoMnMo, it was noticed that Co‐3d orbitals display an obvious peak and mainly dominate the electron density near the E_F_ (Figure 6d). For both Fe‐3d and Mn‐3d orbitals, there are evident e_g_‐t_2g_ splitting effects, which limit their contributions during electron transfer and exchange. In contrast, the e_g_‐t_2g_ splitting in Ru‐4d orbitals is alleviated, which plays an important role in enhancing the electron density near the E_F_. Although Ni‐3d orbitals in RuFeNiMnMo exhibit a sharp peak at E_v_‐1.45 eV (E_v_ denotes 0 eV), they cannot effectively contribute to the electron density near E_F_, indicating the critical role of Co sites in the HEAs (Figure 6e). For RuFeCoNiMnMo, the good overlapping between Co‐3d and Ni‐3d orbitals are evident, which significantly promotes the adsorptions of H_2_O for the generation of *H during the NO_3_RR (Figure 6f). More importantly, there is an evident increase of electron density near the E_F_, especially for Co sites, which is attributed to the strong high‐entropy effect among different metals, leading to improved surface electroactivity for NO_3_RR. The comparisons of electronic structures confirm the significant role of Co sites in guaranteeing the high electroactivity of HEA for NO_3_RR. The average bond length distribution comparisons reveal a gradual shrinkage in bond lengths from RuFeNiMnMo to RuFeCoNiMnMo, which indicates the modulations induced by the Co sites (Figure 6g). Then, the site‐dependent PDOS is unraveled to investigate the electronic structures in RuFeCoNiMnMo. As the main active sites, the Ru‐4d orbitals show upshifted d‐band centers in the *hcp* phase, and the interface (IF) region between *fcc* and *hcp*, supporting the co‐existence of *fcc* and *hcp* phases is beneficial for the electroactivity (Figure 6h). A similar phenomenon is also noted in Co‐3d orbitals, where the IF regions display the highest d‐band center toward the E_F_ to benefit the NO_3_RR process (Figure 6i). However, for Ni sites, the *fcc* phase and the IF regions show slightly upshifted 3d orbitals, which represents a different trend (Figure 6j). These results demonstrate that the co‐existence of the *fcc* and *hcp* phases with sufficient IF regions ensures the high electroactivity of RuFeCoNiMnMo. From RuFeNiMnMo to RuFeCoNiMnMo, the charge analysis unravels that all metals display increased charges with enlarged valence states in RuFeCoNiMnMo, indicating the introduction of Co sites promotes the electronic optimizations (Figure 6k). Among all the metals, Ru and Mo sites show the most positive charge, and the Ni and Co sites attract electrons with the most negative charges. As the main active sites, Ru with positive charges facilitates the adsorptions of NO_3_^−^ in the solution, while the Ni and Co sites promote the dissociation of H_2_O to supply sufficient protons for NO_3_RR. In addition, the highest W_f_ of RuFeCoNiMnMo also enhances the adsorptions of nitrate, which is consistent with the experimental characterizations (Figure 6l).

The NO_3_RR performance was further explored from an energetic perspective by highlighting the adsorption energies and reaction trends. The adsorption energies of key intermediates display an order of RuFeCoMnMo > RuFeNiMnMo > RuFeCoNiMnMo, revealing that RuFeCoNiMnMo is prone to activate the nitrate due to the strongest binding strength (Figure 6m). It is noted that the adsorption energies of *NO_2_ are higher than both *NO_3_ and *NO, representing that the conversion from *NO_3_ to *NO_2_ induces potential energy barriers. The reaction energetics also confirm that all three HEA catalysts meet an evident barrier for the reduction of *NO_3_ to *NO_2_, in which RuFeCoNiMnMo exhibits the smallest energy barrier (Figure 6n). For the competition between *NHO and *NOH, both reaction pathways are possible, where RuFeCoMnMo and RuFeNiMnMo favor the *NHO pathway while RuFeCoNiMnMo undergoes the *NOH pathway, which agrees well with the in situ DEMS results. For the *NHO pathway, the dehydration of *NH_2_OH to *NH_2_ shows an energy barrier of 0.54 and 0.55 eV for RuFeCoMnMo and RuFeNiMnMo, respectively. Meanwhile, the RuFeCoNiMnMo meets the rate‐determining step (RDS) at the conversion of *NOH to *N with an energy cost of 0.49 eV. Compared to RuFeCoMnMo and RuFeNiMnMo, RuFeCoNiMnMo displays the most preferred reaction trends for NO_3_RR with the lowest barriers. For the competing side reaction of NO_3_RR, the HER trends are also demonstrated (Figure 6o). Although RuFeCoNiMnMo shows the strongest dissociation of water, the strong binding of protons largely results in the largest barrier of 0.32 eV for the formation of H_2_, representing the reduced selectivity to the HER during the NO_3_RR.

## 3 Conclusion

In summary, we have developed the scalable heterophase *fcc‐hcp‐fcc* RuFeMMnMo (M═CoNi, Co, and Ni) HEAs as a promising electrocatalyst for NO_3_RR. The high‐entropy effect with tailored electron divergence and heterophase arrangement of metal atoms result in superior electrocatalytic performance. Notably, RuFeCoNiMnMo HEAs present the NH_3_ FE of near 100% at low overpotential (−0.3 V (vs. RHE)), and above 90% within a wide potential range (from −0.2 to −0.6 V (vs. RHE)) in a neutral environment. The NH_3_ yield rate could reach up to 83.35 mg h^−1^ mg_cat_^−1^ at −0.6 V (vs. RHE). Even at a low nitrate concentration of 10 mm, an excellent FE of 95.4 % at −0.4 V (vs. RHE), together with NH_3_ yield rate of 17.03 mg h^−1^ mg_cat_^−1^ at −0.6 V (vs. RHE) could still be achieved. In addition, RuFeCoNiMnMo HEAs demonstrate superior catalytic durability during the 20 consecutive electrolysis cycles. Ex/in situ characterizations and DFT calculations have revealed that the RuFeCoNiMnMo HEAs possess the strongest interactions among metals with the shortest average bond lengths, which induce surface charge redistributions to promote the activity of the main active sites. Accordingly, the overall NO_3_RR reaction trend of RuFeCoNiMnMo HEAs has been largely enhanced with reduced energy barriers. This work suggests the high feasibility and interesting cocktail effect of unconventional phase high‐entropy alloys for boosting NO_3_RR toward practical scenarios and large‐scale applications.

## Conflicts of Interest

The authors declare no conflicts of interest.

## Supporting information




**Supporting File**: adma72099‐sup‐0001‐SuppMat.docx.

## Data Availability

The data that support the findings of this study are available from the corresponding author upon reasonable request.
