# Supplementary material for: Heterophase fcc‐hcp‐fcc High‐Entropy Alloy Nanomaterials with Tailored Electron Divergence for Selective Ammonia Electrosynthesis
Source: Adv Mater. 2026 Jan 22;38(12):e21096. doi: 10.1002/adma.202521096 (PMC12933016; doi:10.1002/adma.202521096)
Supplement: Supplementary file 1 — Supporting File: adma72099‐sup‐0001‐SuppMat.docx. [file ADMA-38-e21096-s001.docx]

Supporting Information

**Heterophase *fcc-hcp-fcc* High-Entropy Alloy Nanomaterials with Tailored Electron Divergence for Selective Ammonia Electrosynthesis**

Xiang Meng, Mingzi Sun, Xinyi Li, Jing Chen, Yunhao Wang, Fengkun Hao, Fu Liu, Quanlin Zhao, Liang Guo, Yuecheng Xiong, Juan Wang, Guozhi Wang, Mingzheng Shao, Chaohui Wang, Xiaoqing Huang, Qinghua Zhang,^*^ Xiao Zhao,^*^ Bolong Huang,^*^ and Zhanxi Fan^*^

**Table of Contents**

**Experimental Methods**

**Figure S1.** (a) HAADF-STEM, (b) bright-field STEM, and (c-d) TEM images of RuFeCoNiMnMo HEAs.

**Figure S2.** A side-view HRTEM image of RuFeCoNiMnMo HEAs.

**Figure S3.** TEM images of RuFeCoNiMnMo HEAs at different reaction times: (a,b) 3 h, (c,d) 6 h, and (e,f) 24 h.

**Figure S4.** SEM-EDS spectra of RuFeCoNiMnMo HEAs at different reaction times: (a) 3 h, (b) 6 h, and (c) 24 h. Insets: pie charts showing the atomic ratios of different metal elements.

**Figure S5.** XRD patterns of RuFeCoNiMnMo HEAs at different reaction times of 3 h, 6 h, and 24 h.

**Figure S6.** TEM images of RuFeCoNiMnMo HEAs with different dosages of Mo(CO)_6_: (a,b) 11 mg, (c,d) 33 mg, and (e,f) 44 mg.

**Figure S7.** SEM-EDS spectra of RuFeCoNiMnMo HEAs with different dosages of Mo(CO)_6_: (a) 11 mg, (b) 33 mg, and (c) 44 mg. Insets: pie charts showing the atomic ratios of different metal elements.

**Figure S8.** XRD patterns of RuFeCoNiMnMo HEAs with different dosages of Mo(CO)_6_ (11 mg, 33 mg, and 44 mg).

**Figure S9.** TEM images of RuFeCoNiMnMo HEAs with different reaction temperatures: (a,b) 180 ºC, and (c,d) 220 ºC.

**Figure S10.** SEM-EDS spectra of RuFeCoNiMnMo HEAs with different reaction temperatures: (a) 180 ºC, and (b) 220 ºC. Insets: pie charts showing the atomic ratios of different metal elements.

**Figure S11.** XRD patterns of RuFeCoNiMnMo HEAs with different reaction temperatures (160 ºC, 180 ºC, and 220 ºC).

**Figure S12.** TEM images of RuFeCoNiMnMo HEAs with different concentrations of precursor (taking the concentration of Ru_3_(CO)_12_ in OAm as the standard): (a,b) 3.2 mg mL^-1^, (c,d) 0.8 mg mL^-1^, and (e,f) 0.4 mg mL^-1^.

**Figure S13.** TEM images of RuFeCoNiMnMo HEAs with different dosages of Ru_3_(CO)_12_: (a,b) 7 mg, and (c,d) 5 mg.

**Figure S14.** (a) HAADF-STEM, (b,c) TEM, and (d) bright-field STEM images of RuFeNiMnMo HEAs.

**Figure S15.** (a) HAADF-STEM image, (b,c) atomic resolution HAADF-STEM images (Insets: FFT patterns corresponding to the regions marked by red dashed squares), and (d) EDS elemental mappings of RuFeNiMnMo HEAs.

**Figure S16.** (a) HAADF-STEM, (b,c) TEM, and (d) bright-field STEM images of RuFeCoMnMo HEAs.

**Figure S17.** (a) HAADF-STEM image, (b,c) atomic resolution HAADF-STEM images (Insets: FFT patterns corresponding to the regions marked by red dashed squares), and (d) EDS elemental mappings of RuFeCoMnMo HEAs.

**Figure S18.** XRD patterns of RuFeNiMnMo and RuFeCoMnMo HEAs.

**Figure S19.** SEM-EDS spectra of (a) RuFeNiMnMo and (b) RuFeCoMnMo HEAs. Insets: pie charts showing the atomic ratios of different metal elements.

**Figure S20.** TEM images of (a,b) RuMoCoNiMn and (c,d) RuMoCoNiFe alloys.

**Figure S21.** SEM-EDS spectra of (a) RuMoCoNiMn and (b) RuMoCoNiFe alloys. Insets: pie charts showing the atomic ratios of different metal elements.

**Figure S22.** TEM images of (a,b) RuMoFeCo, (c,d) RuMoFeNi, (e,f) RuMoFeMn, and (g,h) RuMoCoNi alloys.

**Figure S23.** TEM images of (a,b) RuMoFe and (c,d) RuMoMn alloys.

**Figure S24.** XPS full spectra of RuFeCoNiMnMo, RuFeNiMnMo, and RuFeCoMnMo HEAs.

**Figure S25.** The O 1s XPS spectrum of RuFeCoNiMnMo HEAs.

**Figure S26.** Inverse Fourier transform EXAFS fitting results of Ru for (a) RuFeCoNiMnMo, (b) RuFeNiMnMo, and (c) RuFeCoMnMo HEAs, respectively.

**Figure S27.** Inverse Fourier transform EXAFS fitting results of Co for (a) RuFeCoNiMnMo and (b) RuFeCoMnMo HEAs.

**Figure S28.** Inverse Fourier transform EXAFS fitting results of Ni for (a) RuFeCoNiMnMo and (b) RuFeNiMnMo HEAs.

**Figure S29.** The chronoamperometric curves for (a) RuFeCoNiMnMo, (b) RuFeNiMnMo, and (c) RuFeCoMnMo HEAs, respectively, at various potentials (vs. RHE).

**Figure S30.** The UV-vis calibration curves of NH_3_ using different concentrations of NH_4_Cl solutions as standards. (a) UV-vis curves. (b) The linear fitting result.

**Figure S31.** The UV-vis calibration curves of NO_2_^-^ using different concentrations of KNO_2_ solutions as standards. (a) UV-vis curves. (b) The linear fitting result.

**Figure S32.** The NO_2_^-^ FE for RuFeCoNiMnMo, RuFeNiMnMo, and RuFeCoMnMo HEAs, respectively, at various potentials.

**Figure S33.** The NMR calibration curves of NH_4_^+^ using C_4_H_4_O_4_ as internal standard. (a) ^1^H NMR spectra with different ^14^NH_4_Cl concentrations. (b) The linear fitting result.

**Figure S34.** NMR spectra of the electrolytes after electrolysis at -0.30 V (vs. RHE) without or with K^14^NO_3_ and K^15^NO_3_ as the feeding nitrogen sources.

**Figure S35.** The CV curves for (a) RuFeCoNiMnMo, (b) RuFeNiMnMo, and (c) RuFeCoMnMo HEAs, respectively, at the sweep rates of 100, 120, 140, 160, 180, 200, 220, and 240 mV s^-1^.

**Figure S36.** The chronoamperometric curves for RuFeCoNiMnMo at various potentials with different concentrations of KNO_3_: (a) 0.01 M, (b) 0.05 M, and (c) 0.20 M.

**Figure S37.** The NH_3_ yield rate for RuFeCoNiMnMo HEAs with different concentrations of KNO_3_ (0.01 M, 0.05 M, 0.10 M, and 0.20 M) at various potentials.

**Figure S38.** The NO_2_^-^ FE for RuFeCoNiMnMo HEAs with different concentrations of KNO_3_ (0.01 M, 0.05 M, 0.10 M, and 0.20 M) at various potentials.

**Figure S39.** The NO_2_^-^ FE for RuFeCoNiMnMo HEAs during the consecutive electrolysis of 20 cycles at -0.4 V (vs. RHE).

**Figure S40.** The chronoamperometric curves for RuFeCoNiMnMo HEAs during the consecutive electrolysis of 20 cycles at (a) -0.3 and (b) -0.4 V (vs. RHE).

**Figure S41.** The LSV curves for RuFeCoNiMnMo HEAs after every consecutive electrolysis.

**Figure S42.** (a-c) TEM images, and (d) XRD patterns of RuFeCoNiMnMo HEAs after the electrolysis test. CP: carbon paper.

**Figure S43.** The chronoamperometric curves for RuFeCoNiMnMo HEAs during the long-term electrolysis (refresh the electrolyte every 10 h).

**Figure S44.** The LSV curves for RuFeCoNiMnMo HEAs after the long-term electrolysis.

**Figure S45.** The NMR spectra for RuFeCoNiMnMo HEAs after the long-term electrolysis.

**Figure S46.** Four LSV scan cycles (0.1 to -0.7 V (vs. RHE)) during in-situ DEMS test for (a) RuFeCoNiMnMo, (b) RuFeNiMnMo, and (c) RuFeCoMnMo HEAs, respectively.

**Figure S47.** The chronoamperometric curves for RuFeCoNiMnMo HEAs at 0.2 M TBA.

**Figure S48.** (a) The NH_3_ FE, and (b) NO_2_^-^ FE for RuFeCoNiMnMo HEAs without (*w/o*) TBA and with 0.2 M TBA, respectively.

**Note S1.** Calculation of three prerequisites (the mixing entropy, mixing enthalpy, and atomic radius difference) for the formation of high entropy alloys.

**Table S1.** Calculation of Δ*S*_mix_ of RuFeCoNiMnMo, RuFeNiMnMo, and RuFeCoMnMo HEAs.

**Table S2.** Calculation of Δ*H*_mix_ of RuFeCoNiMnMo, RuFeNiMnMo, and RuFeCoMnMo HEAs.

**Table S3.** Calculation of *δ* of RuFeCoNiMnMo, RuFeNiMnMo, and RuFeCoMnMo HEAs.

**Table S4.** A summary of XPS results for RuFeCoNiMnMo, RuFeNiMnMo, and RuFeCoMnMo HEAs.

**Table S5.** A summary of the Ru *K*-edge EXAFS fitting results for RuFeCoNiMnMo, RuFeNiMnMo, and RuFeCoMnMo HEAs.

**Table S6.** A summary of the Co *K*-edge EXAFS fitting results for RuFeCoNiMnMo and RuFeCoMnMo HEAs.

**Table S7.** A summary of the Ni *K*-edge EXAFS fitting results for RuFeCoNiMnMo and RuFeNiMnMo HEAs.

**Table S8.** The concentration of metal ions dissolved in the electrolyte after the electrolysis, which is detected by ICP-OES.

**Table S9.** Comparison of NO_3_RR performances between HEAs in this work and previously reported high-entropy/multicomponent electrocatalysts.

**Table S10.** Comparison of NO_3_RR performances between RuFeCoNiMnMo HEAs in this work and previously reported Ru-containing electrocatalysts.

**References**

**Experimental Methods**

**Chemicals and reagents.**

Triruthenium dodecacarbonyl (Ru_3_(CO)_12_, 98%) was purchased from Energy Chemical. Nickel(II) acetylacetonate (Ni(acac)_2_, 95%), manganese carbonyl (Mn_2_(CO)_10_, 98%), molybdenumhexacarbonyl (Mo(CO)_6_, 99.9%), ammonium chloride (NH_4_Cl, 99.5%), sodium hydroxide (NaOH, ≥98%), salicylic acid (99.5%), sodium citrate tribasic dihydrate (99%), *p*-aminobenzenesulfonamide (H_2_NC_6_H_4_SO_2_NH_2_, ≥99%), N-(1-naphthyl)ethylenediamine dihydrochloride (98%), phosphoric acid (H_3_PO_4_, ≥85 wt% in H_2_O), oleylamine (OAm, C18: 80-90%), maleic acid (≥99%), and deuterium oxide (D_2_O, 99%) were obtained from Shanghai Aladdin Biochemical Technology Co., Ltd. Iron(III) acetylacetonate (Fe(acac)_3_, 97%), potassium nitrate (KNO_3_, 99%), potassium nitrate-15N (K^15^NO_3_, 98%), and Nafion solution (~ 5 wt%) were supplied by Sigma-Aldrich. Cobalt(II) acetylacetonate (Co(acac)_2_, 97%), potassium sulfate (K_2_SO_4_, 99%), and sodium hypochlorite solution (NaClO, 6-14% active chlorine basis) were purchased from Shanghai Macklin Biochemical Co., Ltd. Sulfuric acid (H_2_SO_4_, 98%), and n-hexane (95%) were purchased from Anaqua Global International Inc. Ltd. Ethanol (absolute, 99.9%) was purchased from Unichem Ltd. Sodium nitroferricyanide solution (C_5_H_4_FeN_6_Na_2_O_3_, 99%) was obtained from Alfa Aesar. All the chemicals and reagents were used as received without further purification. All the aqueous solutions were prepared by deionized (DI) water with a resistance of 18.2 MΩ cm.

**Materials synthesis.**

Heterophase *fcc-hcp-fcc* RuFeMMnMo (M = CoNi, Co, and Ni) high-entropy alloy (HEA) nanoflowers were synthesized by a facile one-pot solvothermal method. In a typical synthesis of RuFeCoNiMnMo HEAs, 8.0 mg of Ru_3_(CO)_12_, 8.0 mg of Fe(acac)_3_, 4.4 mg of Co(acac)_2_, 3.3 mg of Ni(acac)_2_, 4.9 mg of Mn_2_(CO)_10_, and 22 mg of Mo(CO)_6_ were dissolved in 5 mL of OAm, followed by ultrasonication for 5 h to obtain the homogenous solution. The mixture was then transferred to the Teflon-lined autoclave (25 mL capacity), and was heated at 200 ºC for 12 h. After the reaction, the product was collected by centrifugation and washed four times with n-hexane/ethanol (v/v = 3/1) mixture.

For the synthesis of RuFeNiMnMo and RuFeCoMnMo HEAs, all the experimental conditions are similar to those of RuFeCoNiMnMo HEAs, except that Co(acac)_2_ or Ni(acac)_2_ was not added, respectively.

**Characterizations.**

Powder X-ray diffraction (XRD) measurement was conducted on a Rigaku SmartLab X-ray diffractometer with Cu K_α_ source (λ = 0.15406 nm). Scanning electron microscope energy dispersive X-ray spectroscopy (SEM-EDS) measurement was conducted through QUANTA 250. Transmission electron microscope (TEM) and high-resolution TEM (HRTEM) images were collected on a JEOL-2100F TEM at an accelerating voltage of 200 kV. The spherical aberration-corrected high-angle annular dark-field scanning TEM (HAADF-STEM) images were obtained on a high-resolution aberration-corrected TEM (JEOL JEM-ARM200F). X-ray photoelectron spectroscopy (XPS) data was collected by Thermo Scientific Nexsa spectrophotometer, and the C 1s peak at 284.6 eV was used as the standard to correct for charging effects. Ultraviolet photoelectron spectroscopy (UPS) test was conducted on the Thermo Fisher Scientific ECSALAB Xi^+^. Inductively coupled plasma optical emission spectroscopy (ICP-OES, Avio 220 Max) was applied to detect the concentration of metal ions. The X-ray absorption spectra (XAS) were collected at the 1W1B beamline station in Beijing Synchrotron Radiation Facility (BSRF). XANES spectra were calibrated and normalized using Athena software. Theoretical phase shifts and amplitude functions were calculated from FEFF 8.4. The extracted EXAFS oscillations were k^2^-weighted and Fourier-transformed to R space. The curve fittings of k^2^-weighted EXAFS data in R space were carried out with Artemis. In-situ differential electrochemical mass spectrometry (DEMS) patterns were recorded on the Linglu DEMS analysis system from Shanghai Linglu Instrument Co., Ltd.

**Electrochemical NO_3_RR measurements.**

Before the NO_3_RR tests, the Nafion 117 membrane was pretreated by 5 wt% H_2_O_2_ solution at 80 ºC for 1 h first, followed by 0.5 M H_2_SO_4_ solution at 80 ºC for 1 h, and finally by DI water at 80 ºC for another 1 h. The electrocatalytic test was performed in a typical H-type cell with Ivium-n-Stat multiple channels electrochemical workstation. In a typical three electrode system, the Pt foil and Ag/AgCl (filled with saturated KCl solution) were used as the counter and reference electrodes, respectively. And the carbon paper coated with 0.2 mg of HEA catalyst acted as the working electrode with an area of 1 cm^2^. In this work, the neutral electrolyte was used, which is composed of 0.5 M K_2_SO_4_ and 0.1 M KNO_3_ in most cases. When exploring the effect of nitrate concentration, the nitrate concentration was changed to 0.01 M, 0.05 M, and 0.20 M, respectively. Before the test, the electrolyte solution was bubbled with high-purity argon for 20 mins to prevent possible side-competitive reactions. Both the cathode and anode chambers of H-cell were filled with 25 mL of electrolyte. The cycle voltammetry (CV) method was used to remove the capping agents of catalysts and activate the catalyst surface by scanning 50 cycles at a sweep rate of 50 mV s^-1^ with 0.5 M K_2_SO_4_ solution. The linear sweep voltammetry (LSV) was conducted at a scan rate of 5 mV s^-1^. The chronoamperometry test was performed at a set of potentials for 0.5 h under the stirring rate of 600 rpm. The electrolyte resistance was recorded at the open circuit potential with frequencies ranging from 100 kHz to 0.1 kHz, and all the measured potentials using the three-electrode system were manually 85% compensated. All the potentials refer to reversible hydrogen electrode (RHE): E (vs. RHE) = E (vs Ag/AgCl) + 0.197 + 0.059 × pH. The electrochemical double-layer capacitance (C_dl_) was measured by CV curves with different scanning rates of 100, 120, 140, 160, 180, 200, 220, and 240 mV s^-1^ at the non-Faradaic region. Then, the electrochemical active surface area (ECSA) was calculated by C_dl_: ECSA = C_dl_/C_s_, where C_s_ refers to the specific capacitance. Two methods were used to test the stability of HEA electrocatalysts. Firstly, consecutive recycling electrolysis was carried out at two different potentials, i.e. -0.3 and -0.4 V (vs. RHE), respectively, for 0.5 h. Secondly, the long-term electrolysis was performed for 10 h without refreshing electrolyte. In most cases of this work, the electrochemical tests were performed within the potential range of 0 to -0.6 V (vs. RHE).

**Product analysis.**

In this work, ammonia (NH_3_) is the main product, and nitrite (NO_2_^-^) is the possible by-product. NH_3_ and NO_2_^-^ were quantified by the indophenol blue method. For the NH_3_ analysis, firstly, electrolyte was taken out after the NO_3_RR test and diluted to the suitable concentration for detection. Typically, 2 mL of diluted electrolyte solution were mixed with 2 mL of A solution (1 M NaOH solution with 5 wt% salicylic acid and 5 wt% trisodium citrate dihydrate), 1 mL of B solution (NaClO solution, 6-14% active chlorine basis), and 0.2 mL of C solution (1 wt% sodium nitroferricyanide solution). After being kept in the dark for 2 h, UV-vis spectrophotometer (Shimadzu-UV1700) was used to record the absorbance value at the wavelength of 655 nm. A set of standard NH_4_^+^ solutions was prepared to build the calibration curve. In addition, NMR method was also introduced to prove the accuracy of relevant data. As for the NO_2_^-^, similar to the NH_3_ analysis, electrolyte was taken out after the test and diluted to the suitable concentration for detection. Then 5 mL of diluted electrolyte were mixed with 0.1 mL of color-developing regent (4 g of *p*-Aminobenzenesulfonamide, 0.2 g of N-(1-Naphthyl)ethylenediamine dihydrochloride, and 10 mL of phosphoric acid were mixed into 50 mL of DI water). After being kept in the dark for 20 min, UV-vis spectrophotometer was used to record the absorbance value at the wavelength of 540 nm. A set of standard NO_2_^-^ solutions was prepared to build the calibration curve.

**Calculations of Faradaic efficiency (FE) and yield rate.**

The FE of NH_3_ was calculated using Eq (1):

$FE ({NH}_{3})=\frac{8\times F\times C\times V}{M\times Q}\times100\%$ Eq (1)

The FE of NO_2_^-^ was calculated using Eq (2):

$FE ({NO}_{2}^{-})=\frac{2\times F\times C\times V}{M\times Q}\times100\%$ Eq (2)

The yield rate of NH_3_ was calculated using Eq (3):

$Yield rate ({NH}_{3})=\frac{C\times V}{m\times t}\times100\%$ Eq (3)

The yield rate of NO_2_^-^ was calculated using Eq (4):

$Yield rate ({NO}_{2}^{-})=\frac{C\times V}{m\times t}\times100\%$ Eq (4)

Where *F* is the Faraday constant (96485 C mol^-1^), *C* is the measured concentration of NH_3_ or NO_2_^-^ (mg L^-1^), *V* is the electrolyte volume in the working electrode chamber (L), *M* is the molar mass of NH_3_ or NO_2_^-^ (mg mol^-1^), *Q* is the total charge passed (C), *m* is the total mass of catalyst (mg), and *t* is the reaction time (h).

**Theoretical calculations setup.**

To investigate the performance of NO_3_RR, DFT calculations were performed through the embedded CASTEP packages to understand the electronic modulations and reaction energetics in HEA catalysts.^[1]^ The generalized gradient approximation (GGA) and Perdew-Burke-Ernzerhof (PBE) functionals were applied for the descriptions of exchange-correlation interactions.^[2-4]^ The plane-wave cutoff energy was set to 380 eV due to the selection of ultrafine quality. Meanwhile, the ultrasoft pseudopotentials were used for all calculations. The Broyden-Fletcher-Goldfarb-Shannon (BFGS) algorithm was applied for all the energy minimizations, where the coarse quality k-point with a separation of 0.07 Å^-1^ was selected to balance calculation accuracy and efficiency.^[5]^

In this work, RuFeCoMnMo, RuFeNiMnMo, and RuFeCoNiMnMo HEAs were constructed by combinations of Ru (111) of the fcc phase and Ru (001) of the hcp phase in a 4×4×1 supercell. The corresponding compositions of each metal element follow the experimental characterizations. For all the catalyst surfaces, a 20 Å vacuum space in the z-axis was introduced to ensure full relaxation. For all the geometry optimization, we have applied stringent convergence criteria including: 1) the total energy difference should not exceed 5×10^-5^ eV/atom, 2) the inter-ionic displacement should be converged to smaller than 0.005 Å, and 3) the Hellmann-Feynman forces should not exceed 0.001 eV/Å.

**In-situ DEMS test.**

The in-situ DEMS patterns were acquired by an electrochemical cell in conjunction with a mass spectrometer. Carbon paper coating with catalysts, Pt foil, and Ag/AgCl were used as the working, counter, and reference electrodes, respectively. The electrolyte was 0.5 M K_2_SO_4_ solution with 0.1 M KNO_3_. The scanning range of LSV was from 0.1 to -0.7 V (vs. RHE). For every sample, four consecutive scans were applied to obtain mass signals without changing electrolyte.

**
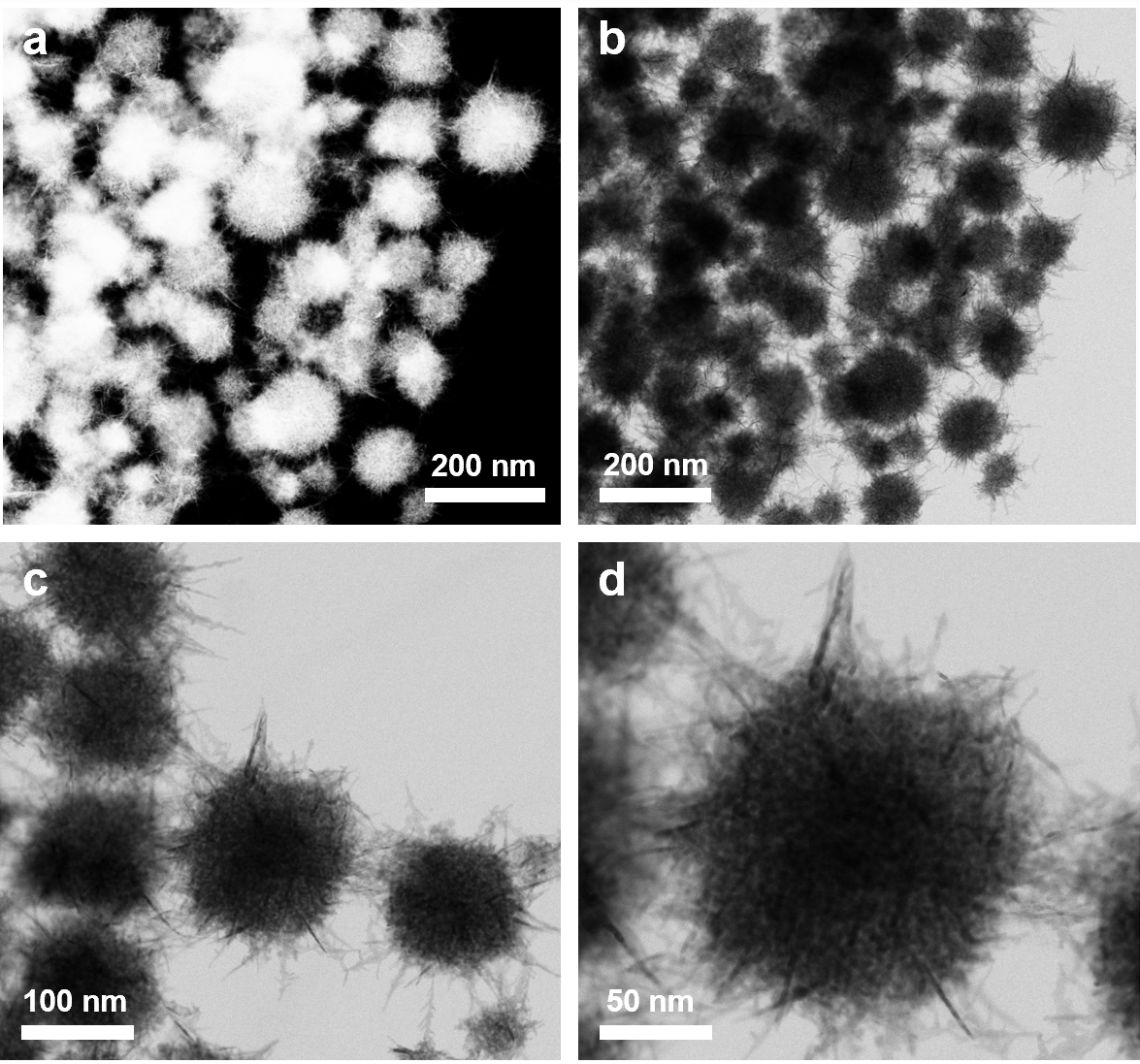
**

**Figure S1.** (a) HAADF-STEM, (b) bright-field STEM, and (c-d) TEM images of RuFeCoNiMnMo HEAs.

**
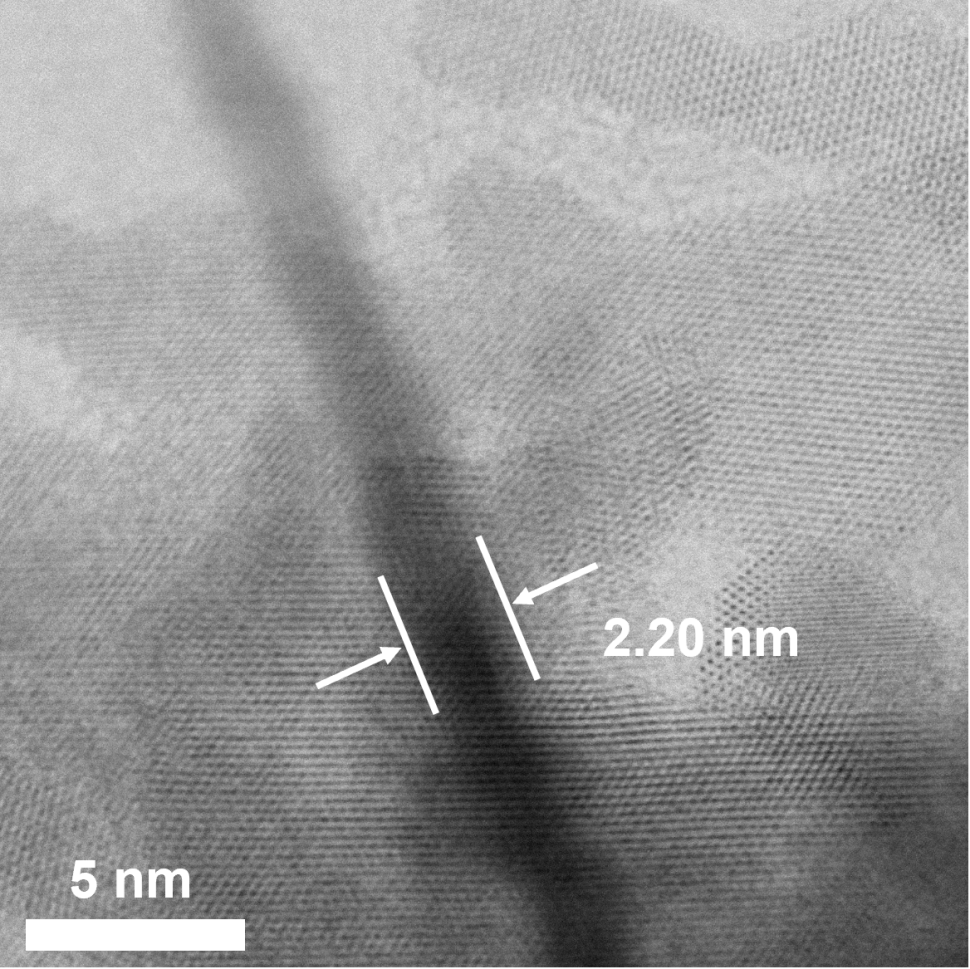
**

**Figure S2.** A side-view HRTEM image of RuFeCoNiMnMo HEAs.


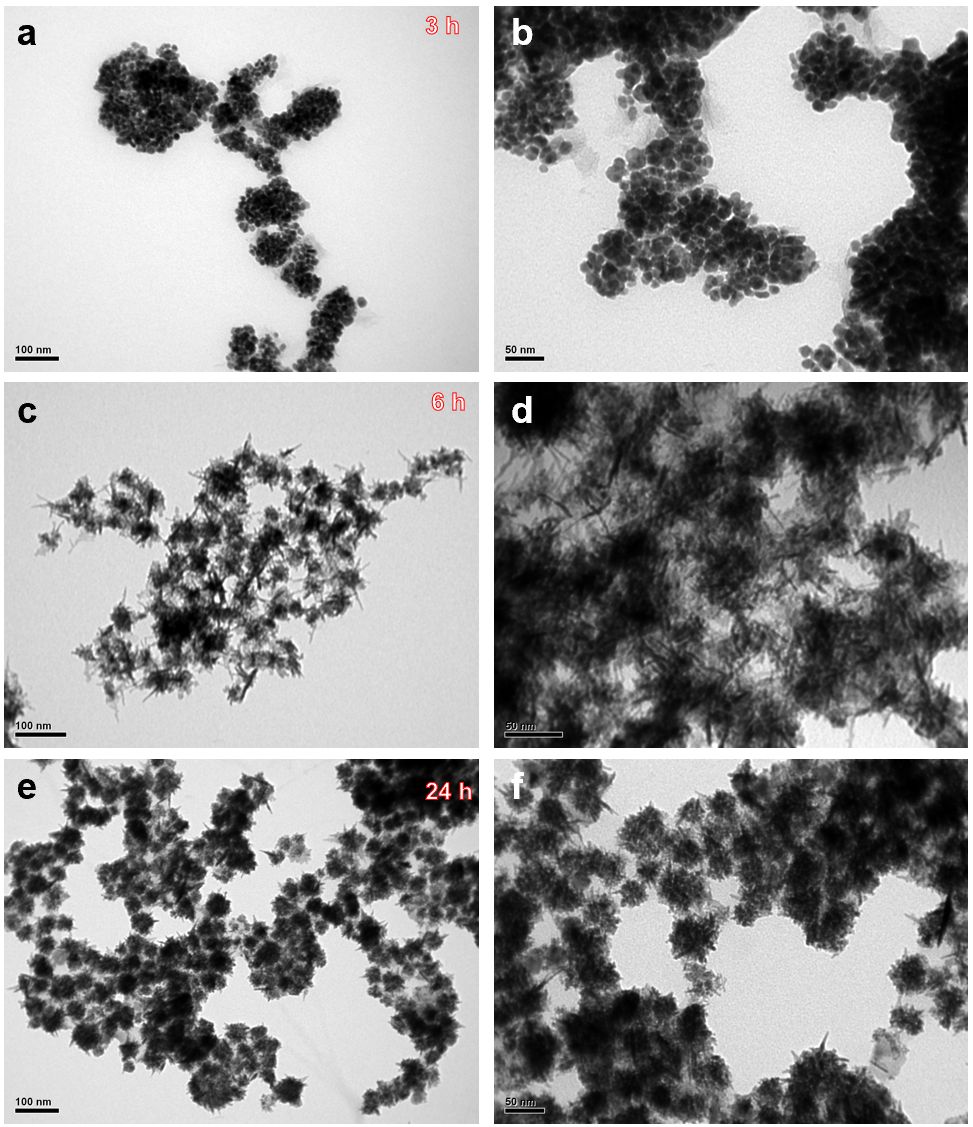


**Figure S3.** TEM images of RuFeCoNiMnMo HEAs at different reaction times: (a,b) 3 h, (c,d) 6 h, and (e,f) 24 h.


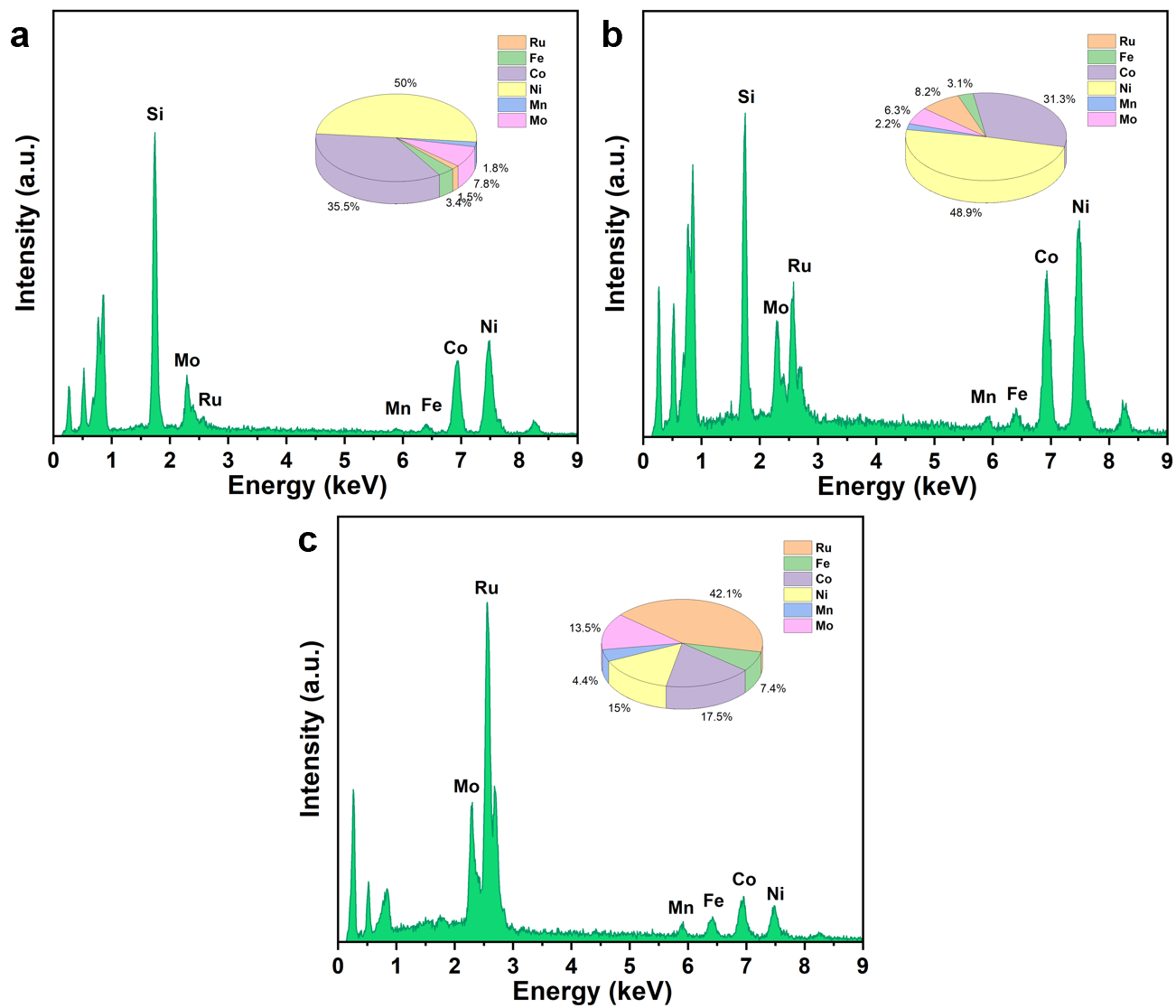


**Figure S4.** SEM-EDS spectra of RuFeCoNiMnMo HEAs at different reaction times: (a) 3 h, (b) 6 h, and (c) 24 h. Insets: pie charts showing the atomic ratios of different metal elements.


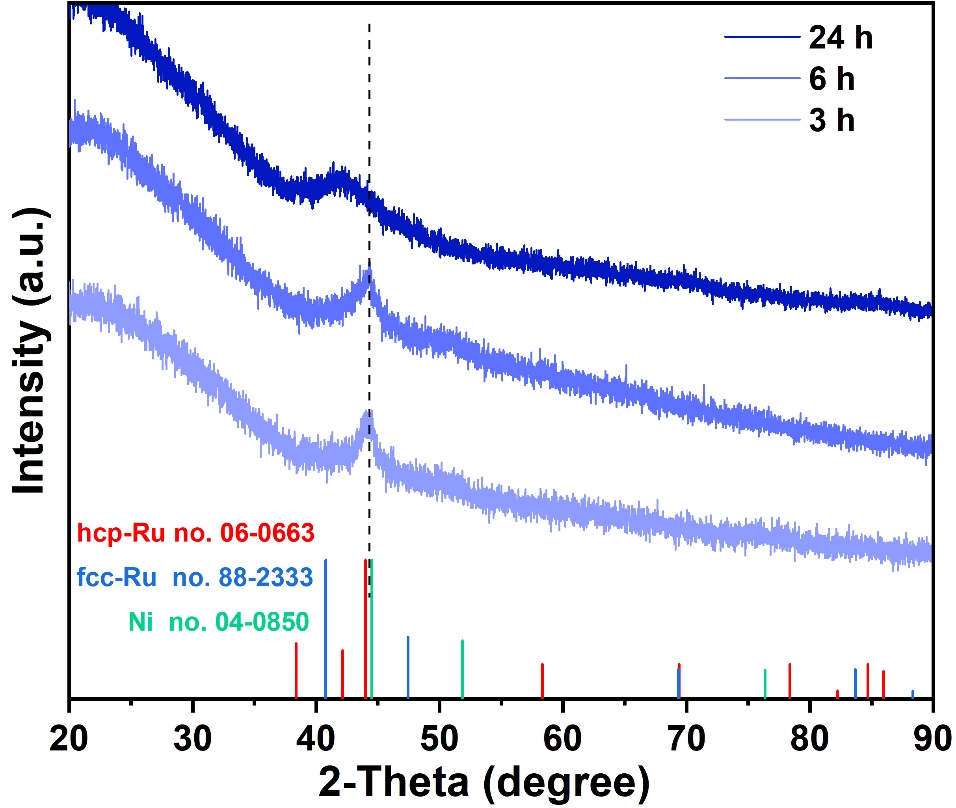


**Figure S5.** XRD patterns of RuFeCoNiMnMo HEAs at different reaction times of 3 h, 6 h, and 24 h.


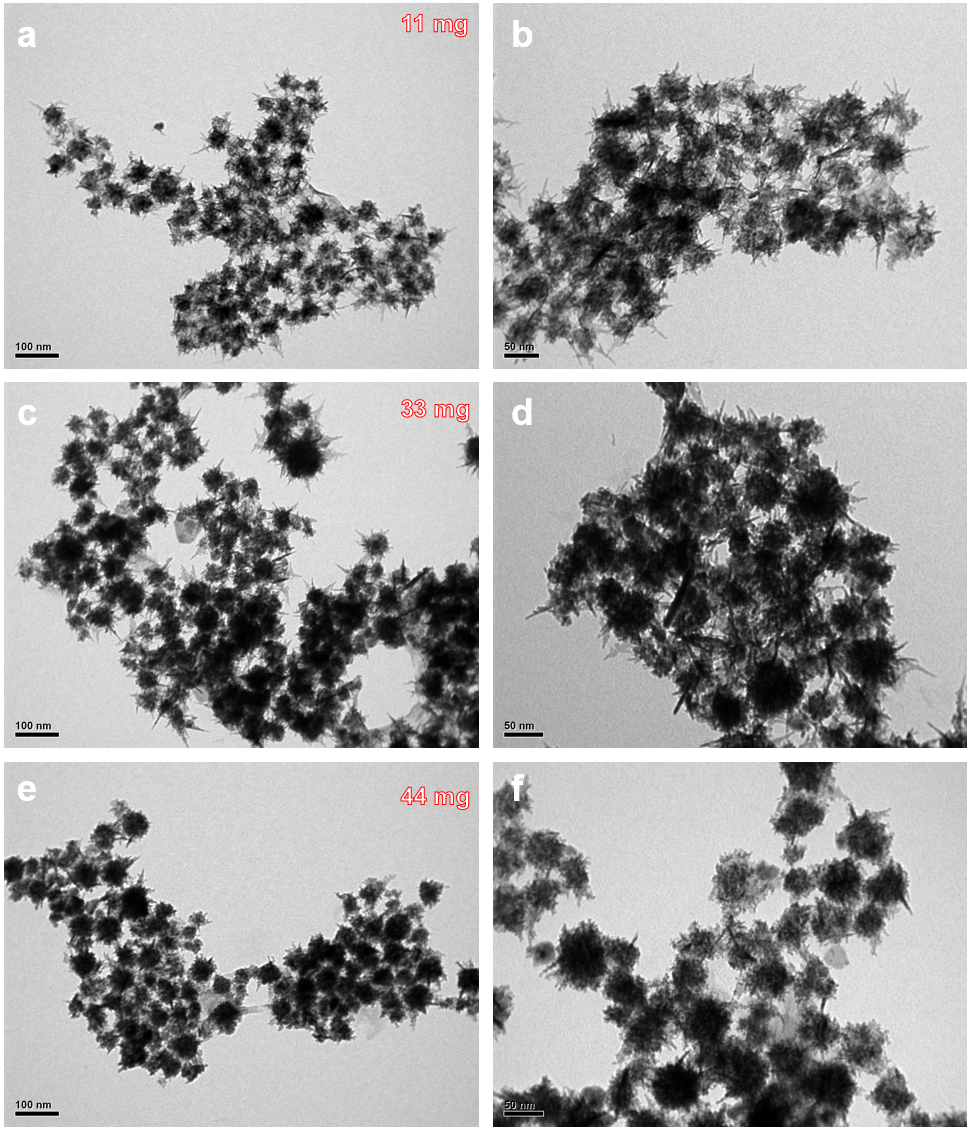


**Figure S6.** TEM images of RuFeCoNiMnMo HEAs with different dosages of Mo(CO)_6_: (a,b) 11 mg, (c,d) 33 mg, and (e,f) 44 mg.


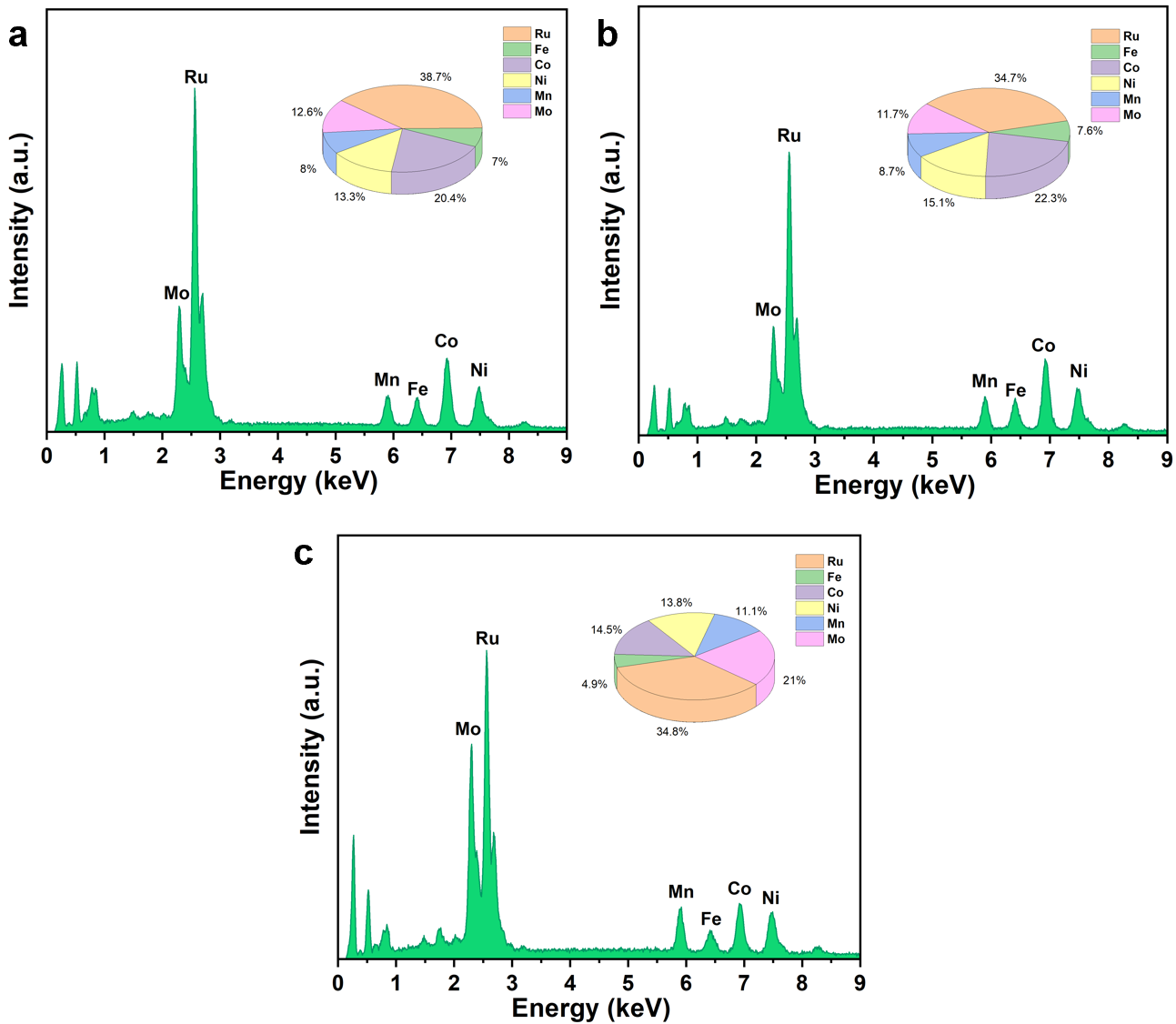


**Figure S7.** SEM-EDS spectra of RuFeCoNiMnMo HEAs with different dosages of Mo(CO)_6_: (a) 11 mg, (b) 33 mg, and (c) 44 mg. Insets: pie charts showing the atomic ratios of different metal elements.


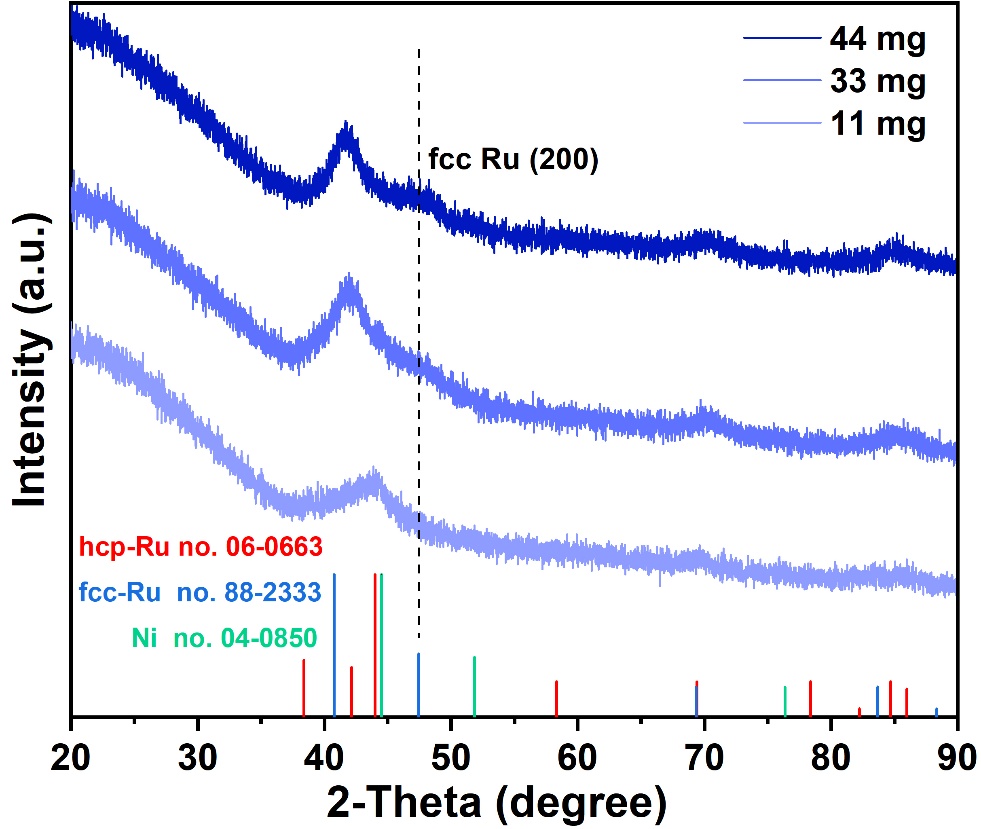


**Figure S8.** XRD patterns of RuFeCoNiMnMo HEAs with different dosages of Mo(CO)_6_ (11 mg, 33 mg, and 44 mg).


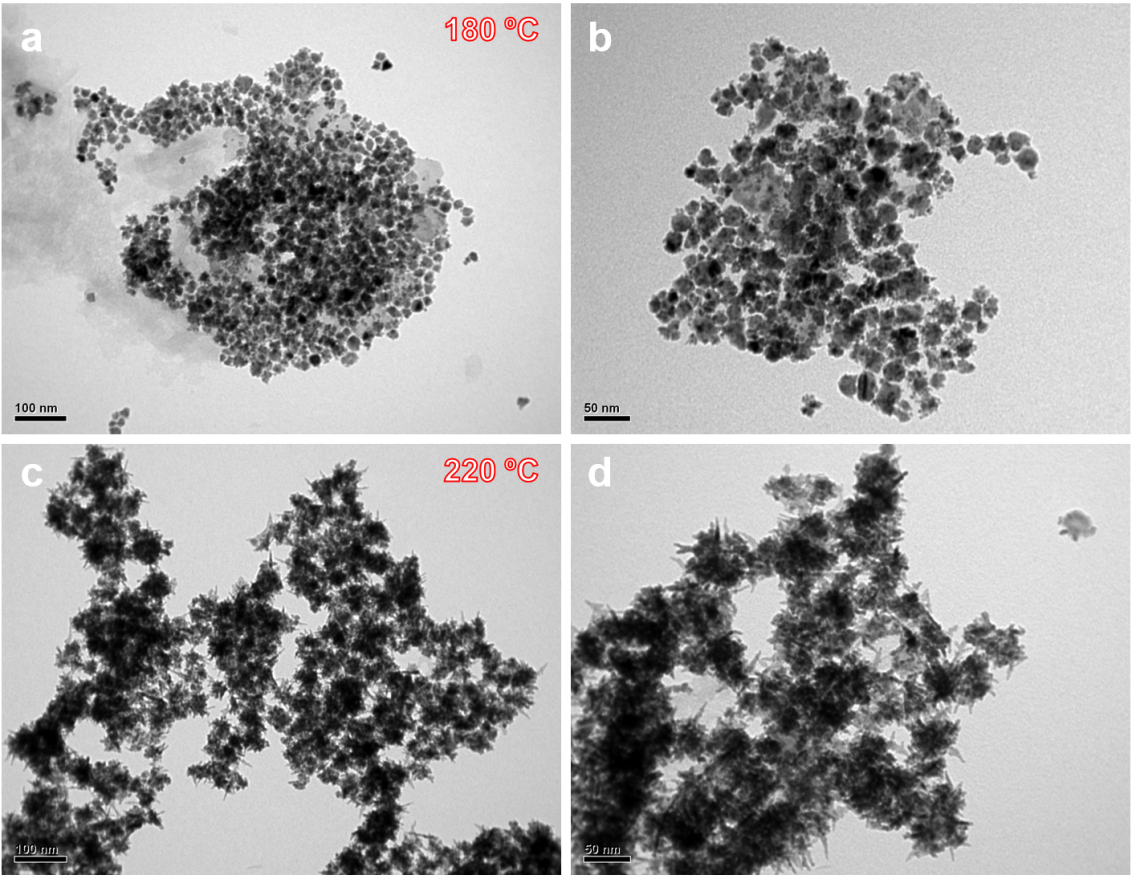


**Figure S9.** TEM images of RuFeCoNiMnMo HEAs with different reaction temperatures: (a,b) 180 ºC, and (c,d) 220 ºC.


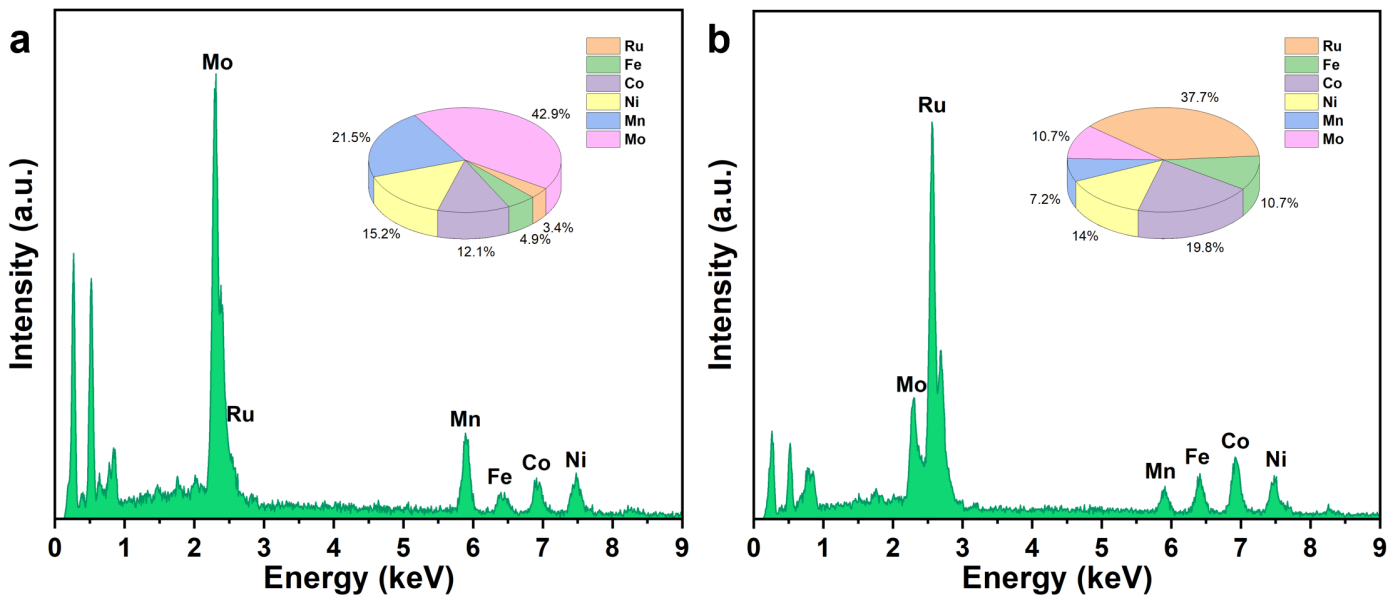


**Figure S10.** SEM-EDS spectra of RuFeCoNiMnMo HEAs with different reaction temperatures: (a) 180 ºC, and (b) 220 ºC. Insets: pie charts showing the atomic ratios of different metal elements.


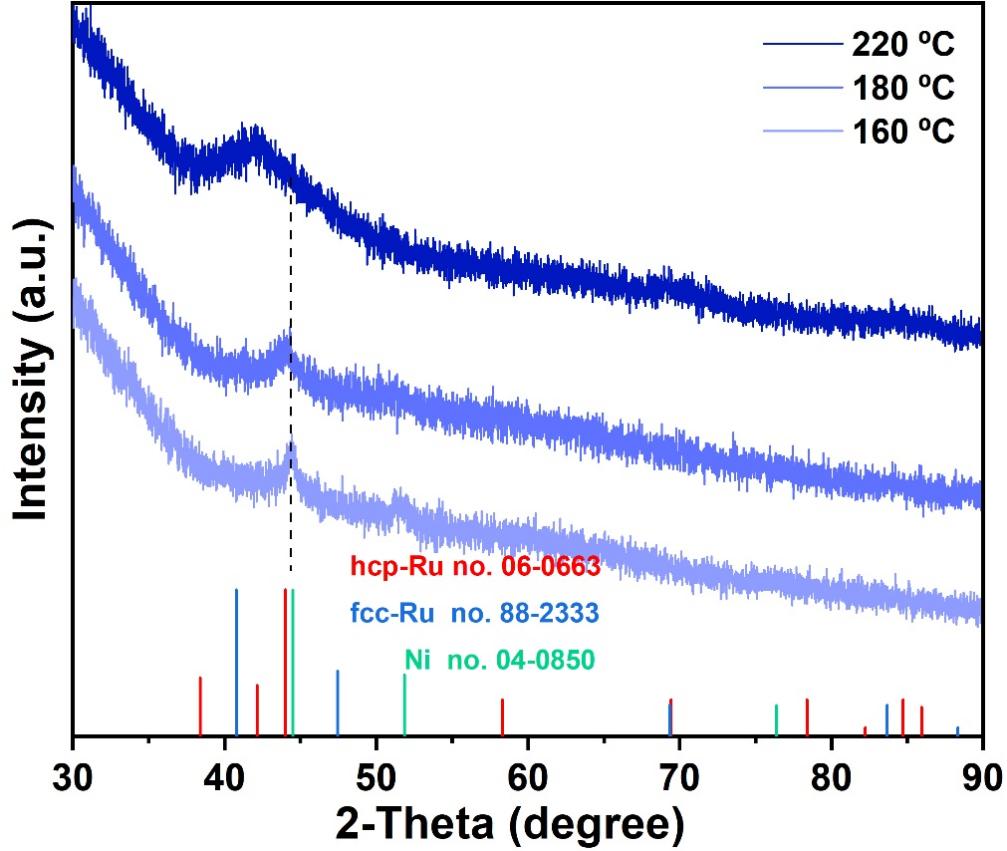


**Figure S11.** XRD patterns of RuFeCoNiMnMo HEAs with different reaction temperatures (160 ºC, 180 ºC, and 220 ºC).


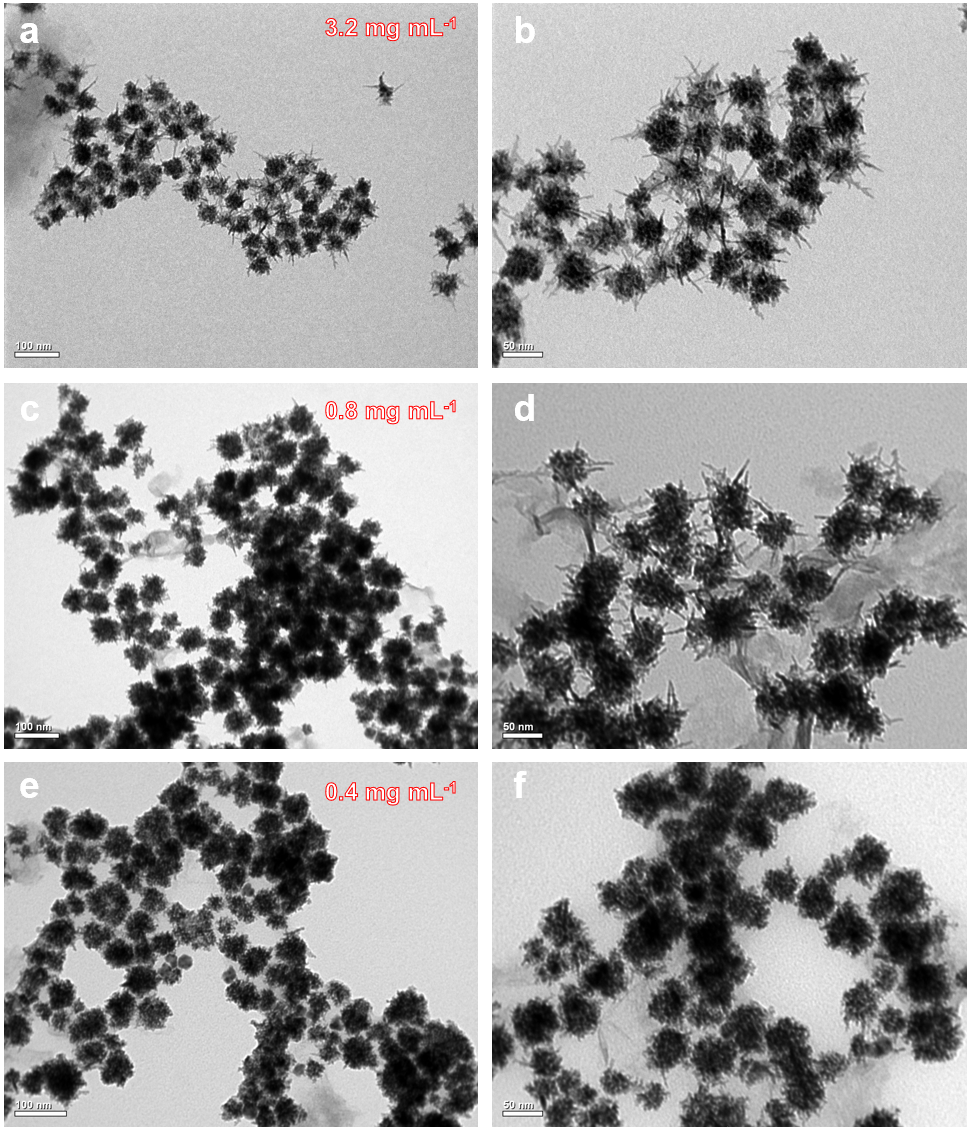


**Figure S12.** TEM images of RuFeCoNiMnMo HEAs with different concentrations of precursor (taking the concentration of Ru_3_(CO)_12_ in OAm as the standard): (a,b) 3.2 mg mL^-1^, (c,d) 0.8 mg mL^-1^, and (e,f) 0.4 mg mL^-1^.


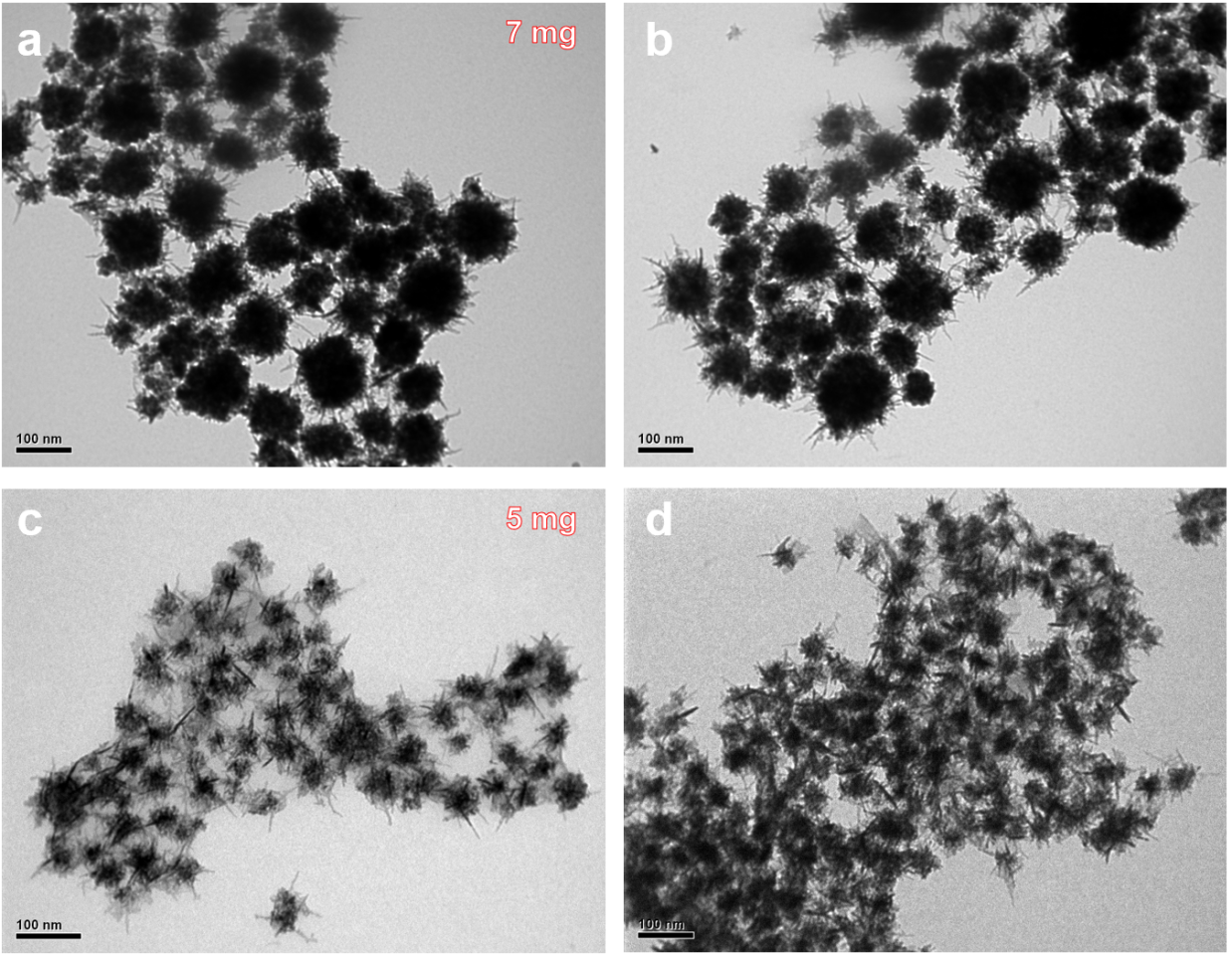


**Figure S13.** TEM images of RuFeCoNiMnMo HEAs with different dosages of Ru_3_(CO)_12_: (a,b) 7 mg, and (c,d) 5 mg.


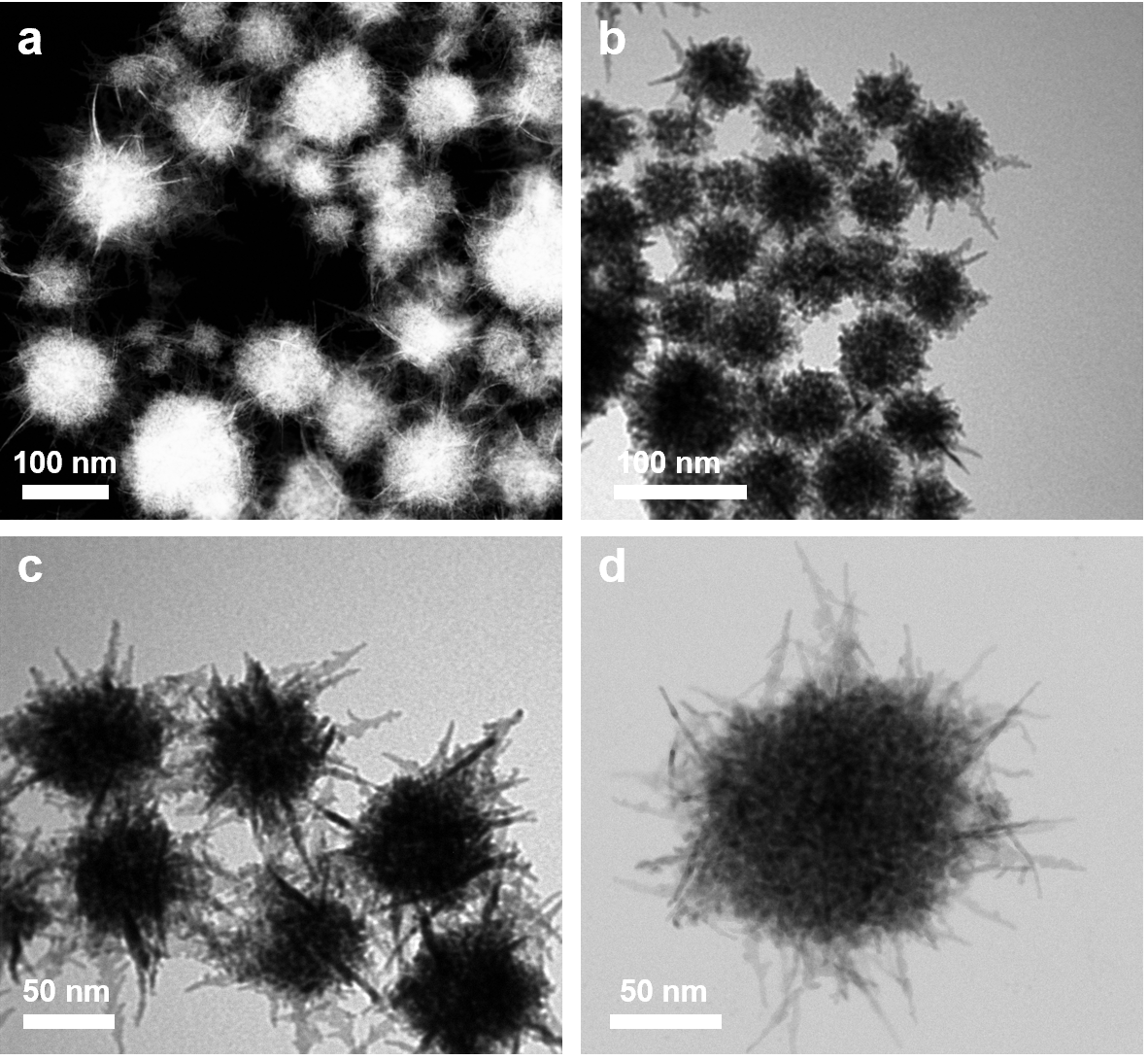


**Figure S14.** (a) HAADF-STEM, (b,c) TEM, and (d) bright-field STEM images of RuFeNiMnMo HEAs.


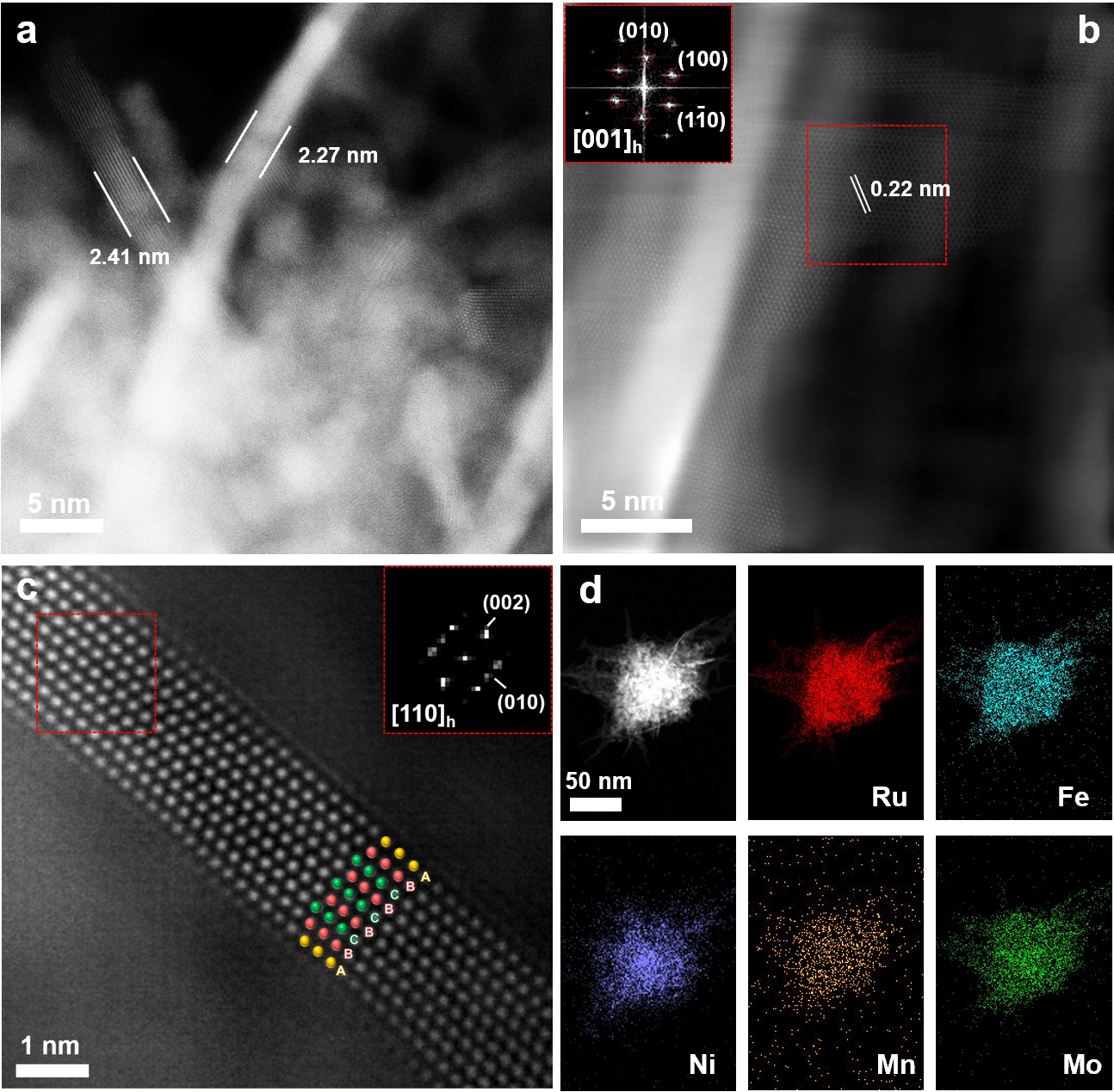


**Figure S15.** (a) HAADF-STEM image, (b,c) atomic resolution HAADF-STEM images (Insets: FFT patterns corresponding to the regions marked by red dashed squares), and (d) EDS elemental mappings of RuFeNiMnMo HEAs.


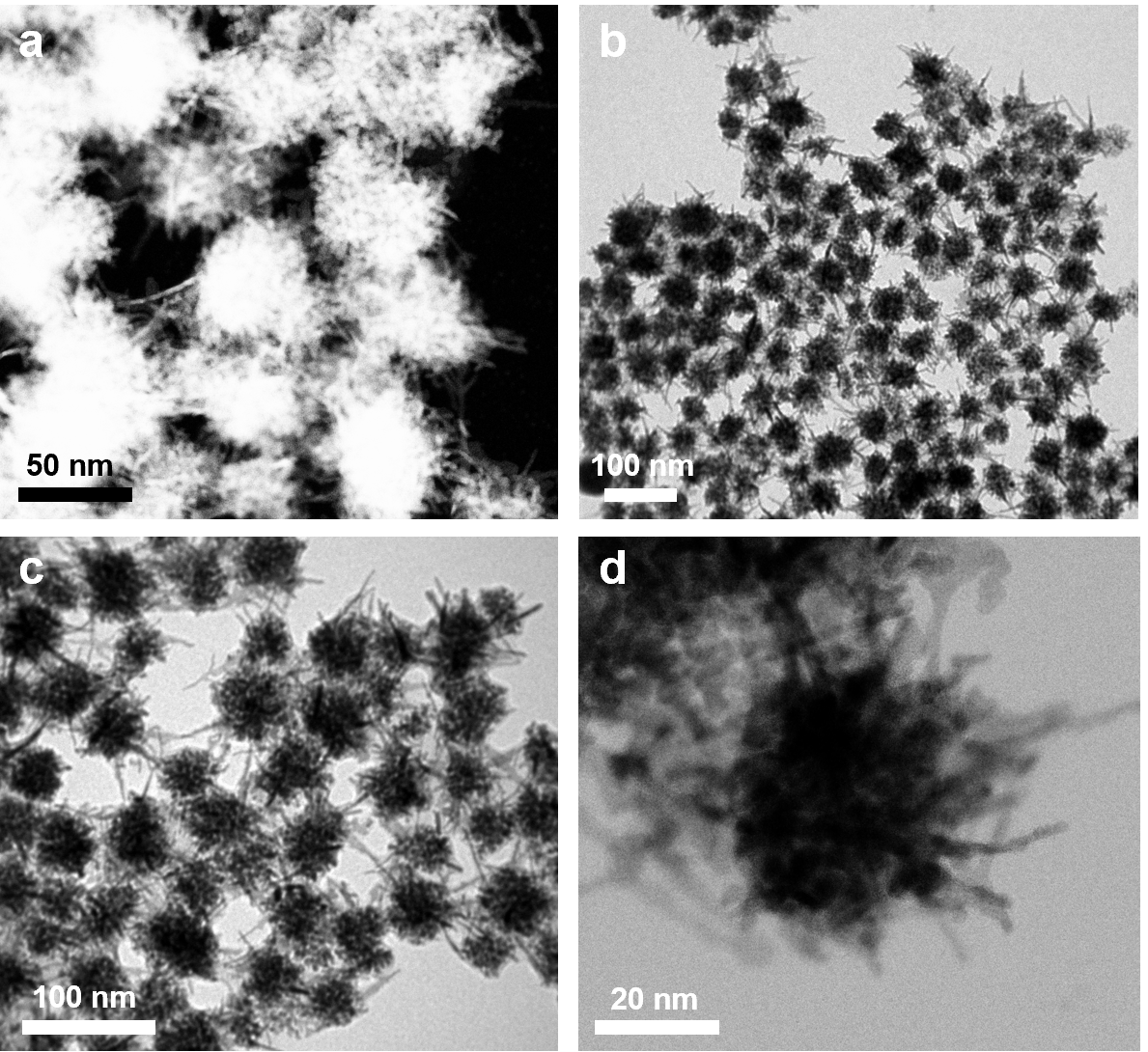


**Figure S16.** (a) HAADF-STEM, (b,c) TEM, and (d) bright-field STEM images of RuFeCoMnMo HEAs.


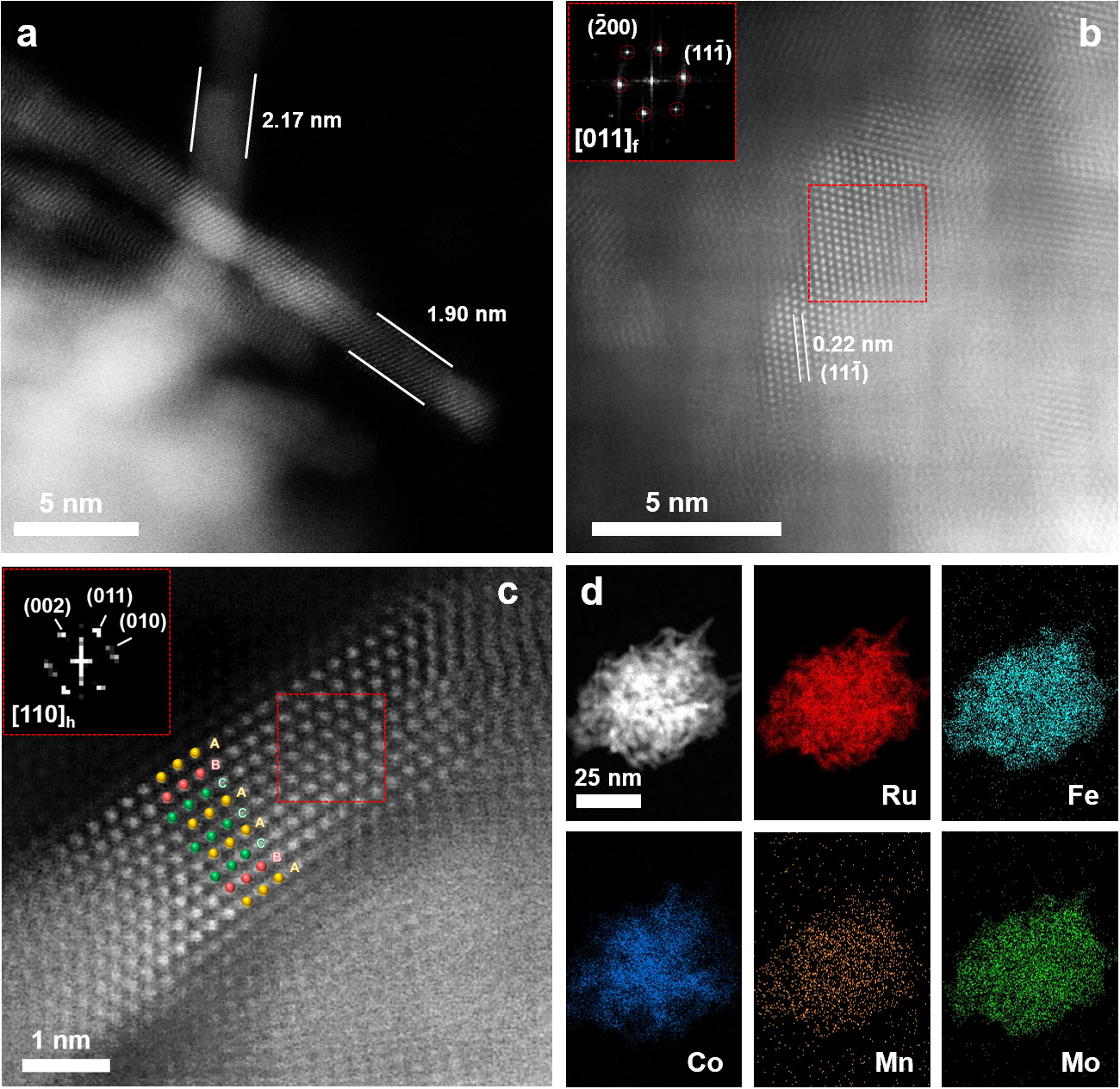


**Figure S17.** (a) HAADF-STEM image, (b,c) atomic resolution HAADF-STEM images (Insets: FFT patterns corresponding to the regions marked by red dashed squares), and (d) EDS elemental mappings of RuFeCoMnMo HEAs.


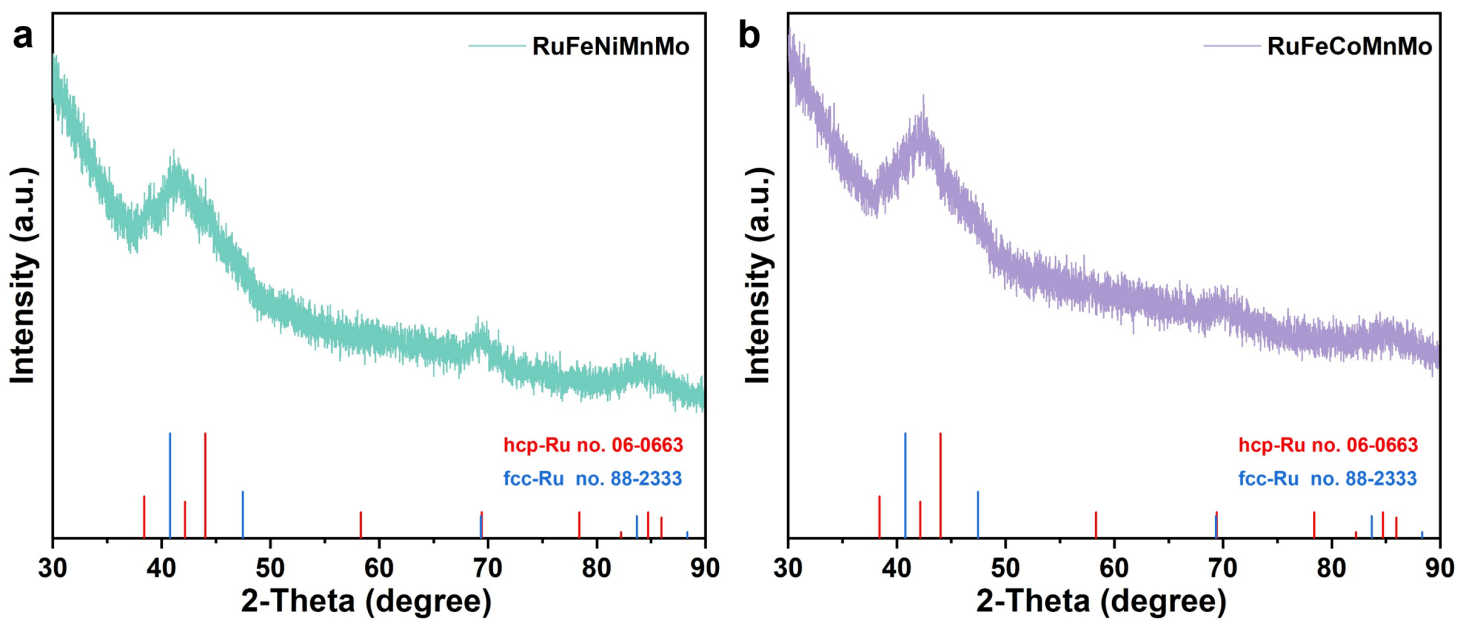


**Figure S18.** XRD patterns of RuFeNiMnMo and RuFeCoMnMo HEAs.


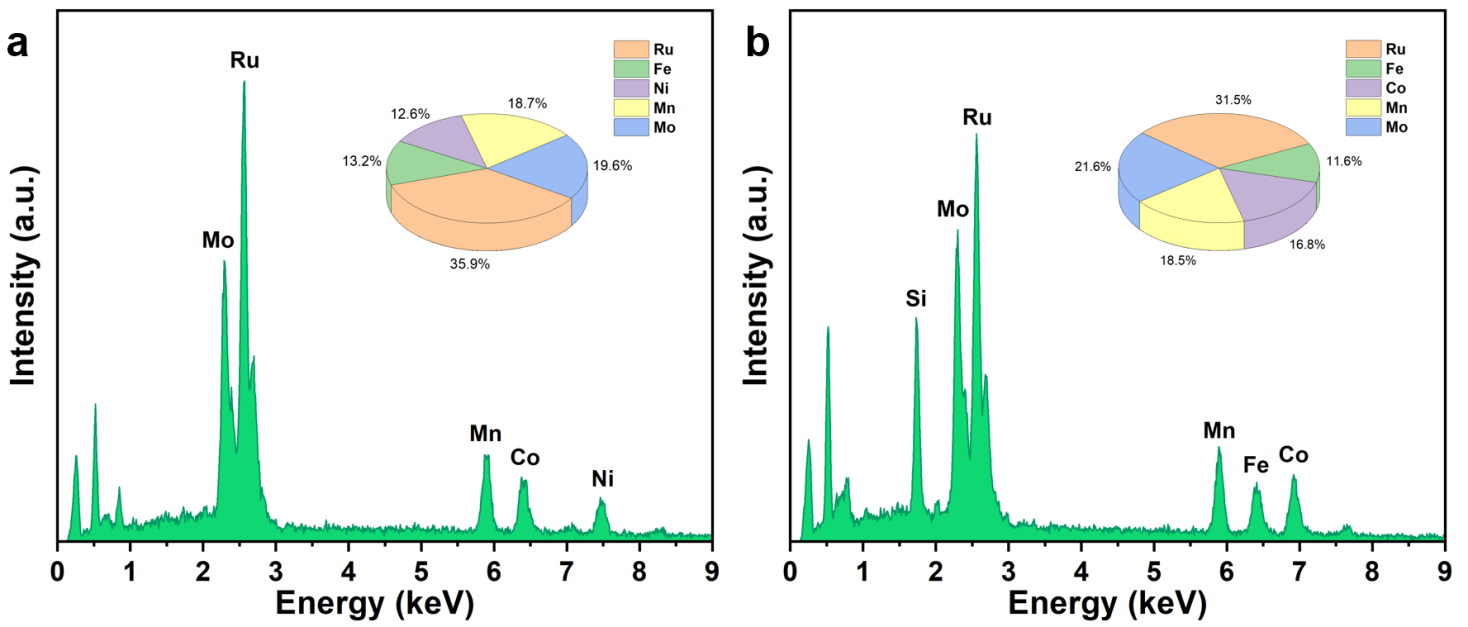


**Figure S19.** SEM-EDS spectra of (a) RuFeNiMnMo and (b) RuFeCoMnMo HEAs. Insets: pie charts showing the atomic ratios of different metal elements.


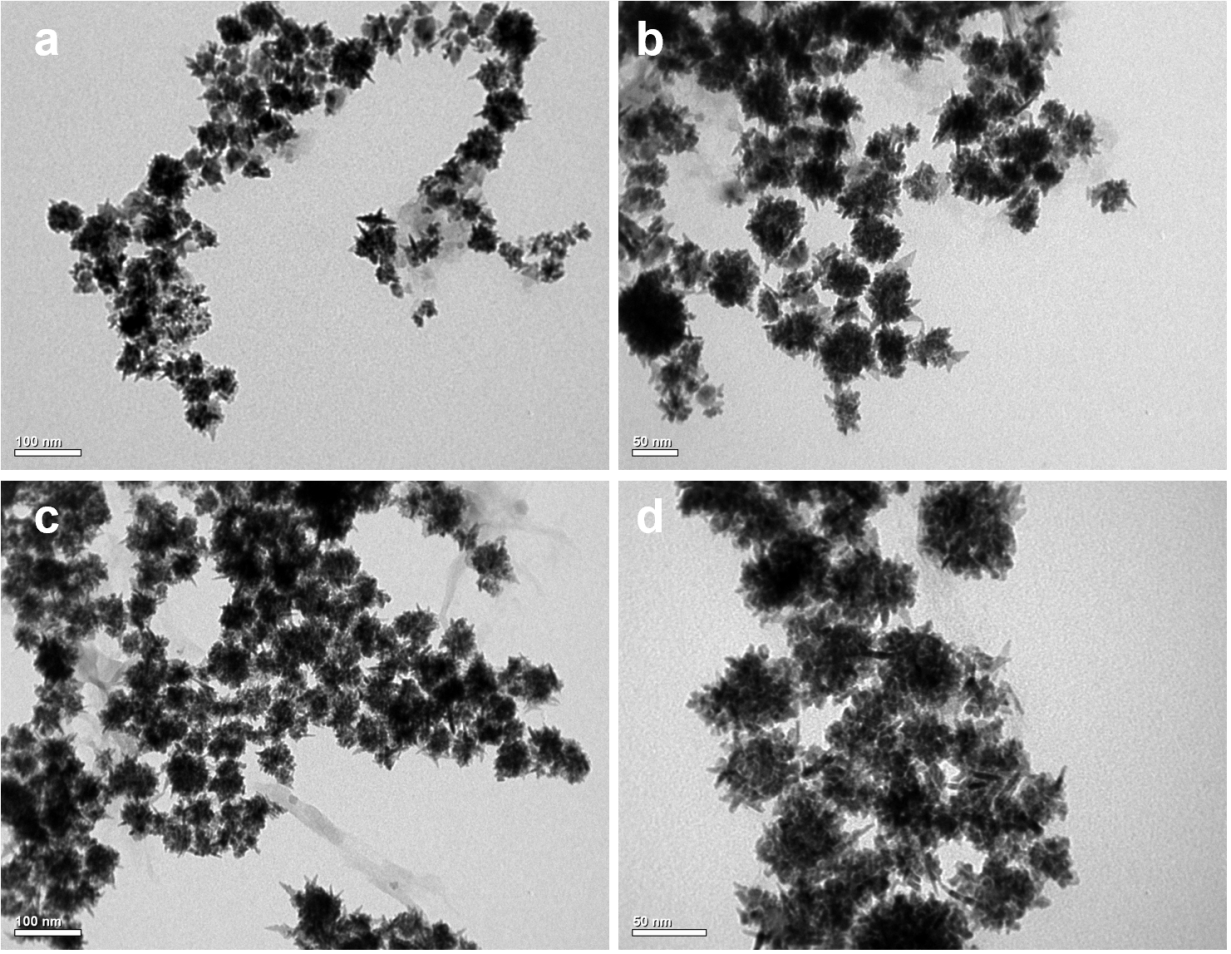


**Figure S20.** TEM images of (a,b) RuMoCoNiMn and (c,d) RuMoCoNiFe alloys.


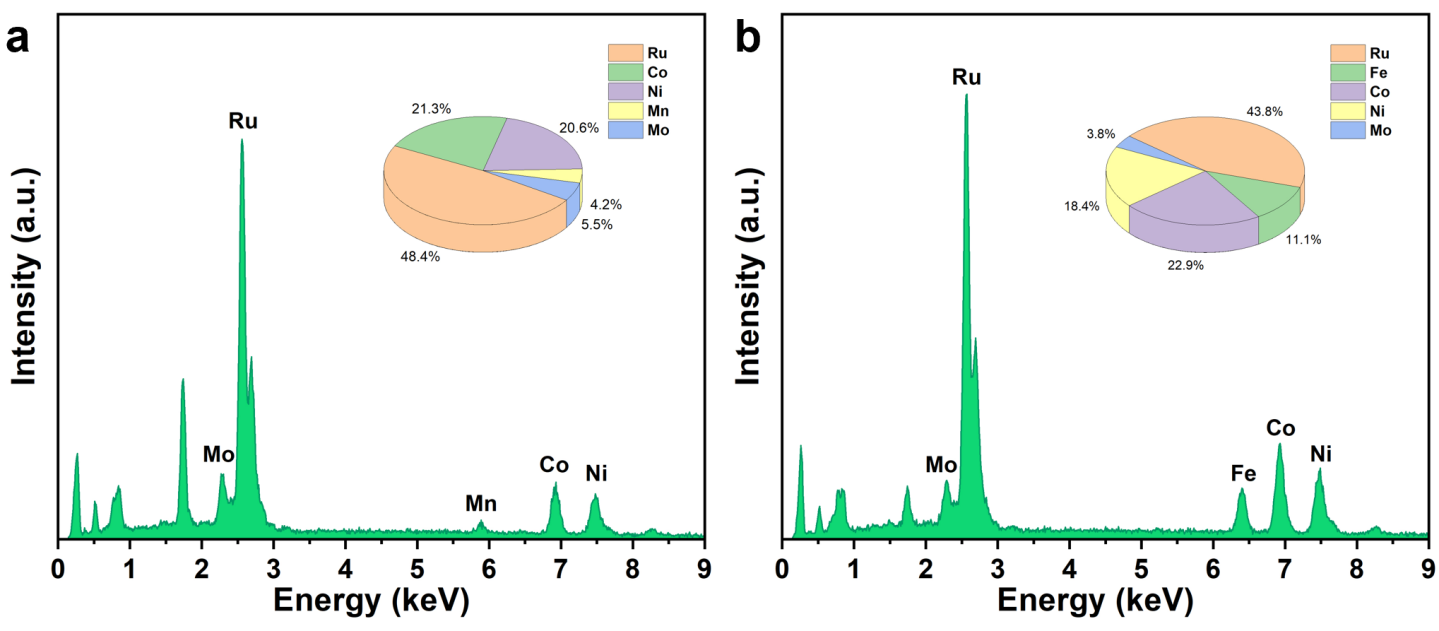


**Figure S21.** SEM-EDS spectra of (a) RuMoCoNiMn and (b) RuMoCoNiFe alloys. Insets: pie charts showing the atomic ratios of different metal elements.


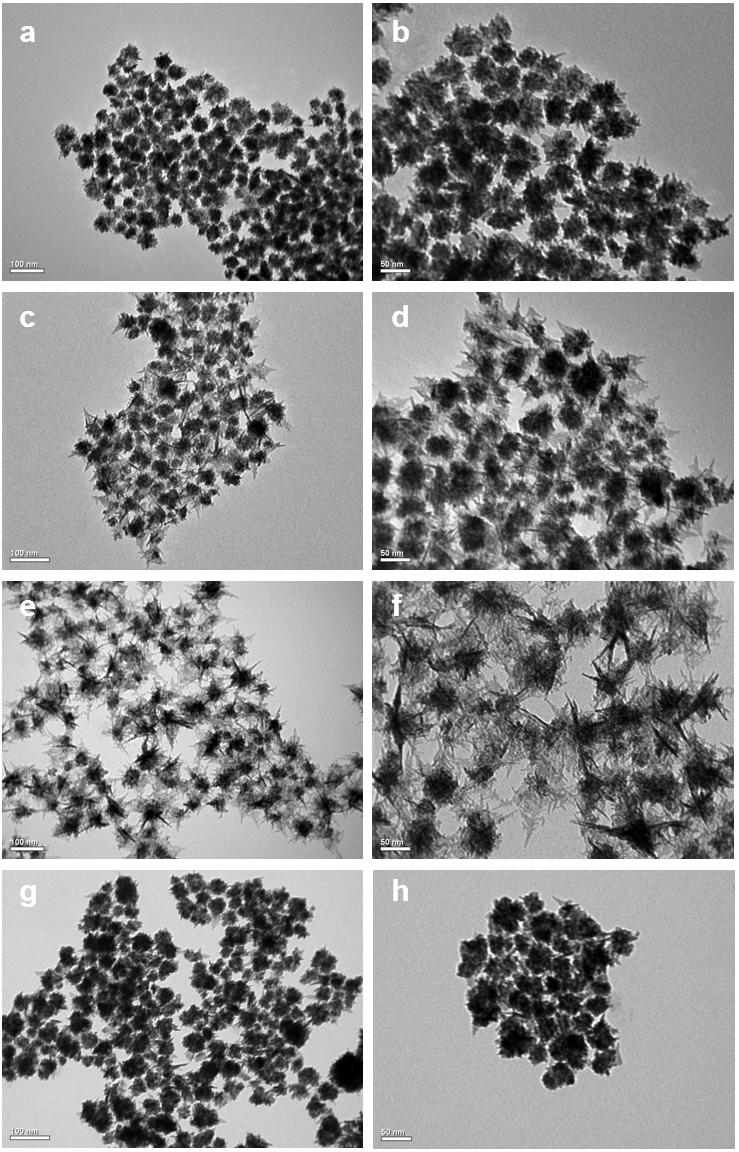


**Figure S22.** TEM images of (a,b) RuMoFeCo, (c,d) RuMoFeNi, (e,f) RuMoFeMn, and (g,h) RuMoCoNi alloys.


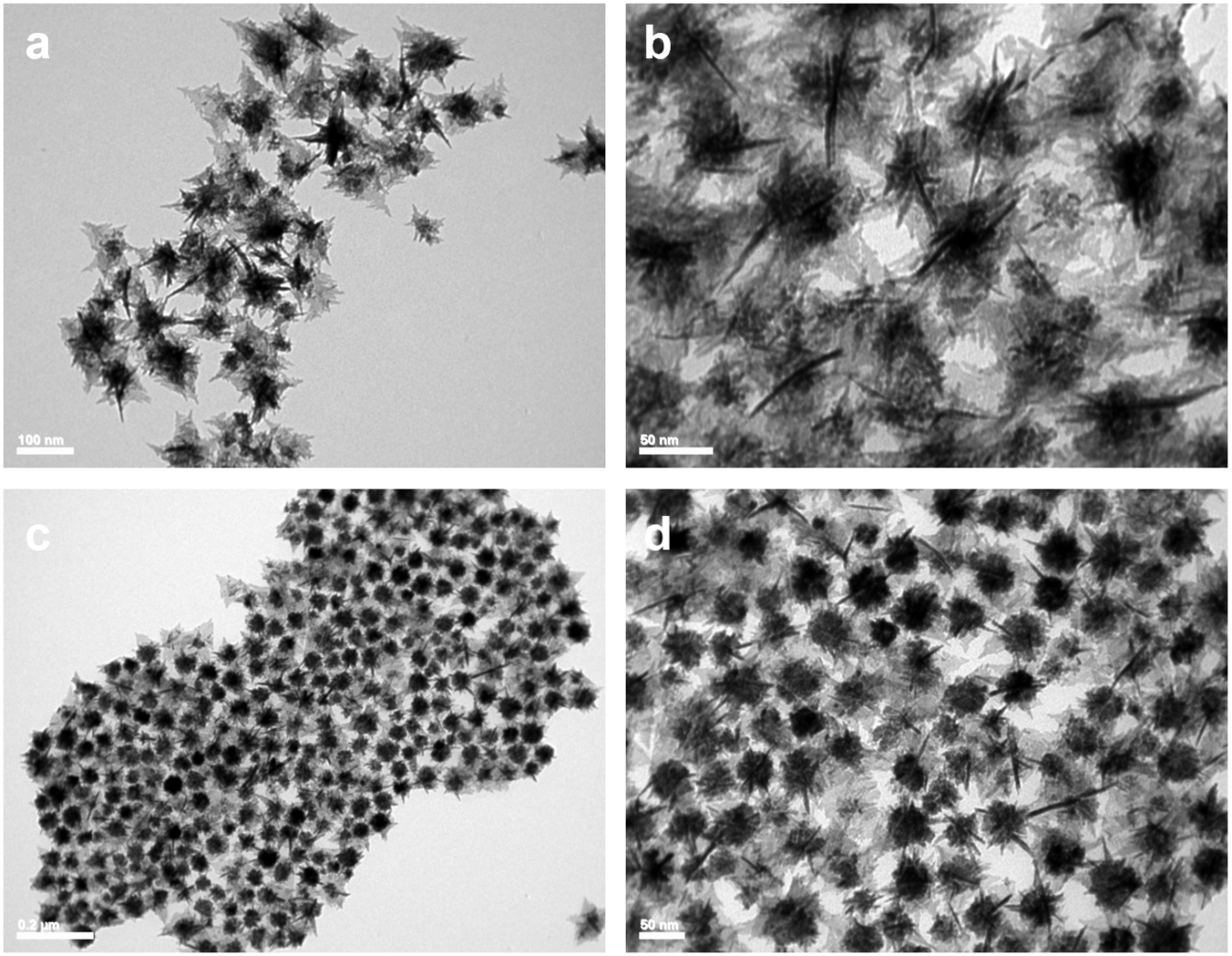


**Figure S23.** TEM images of (a,b) RuMoFe and (c,d) RuMoMn alloys.


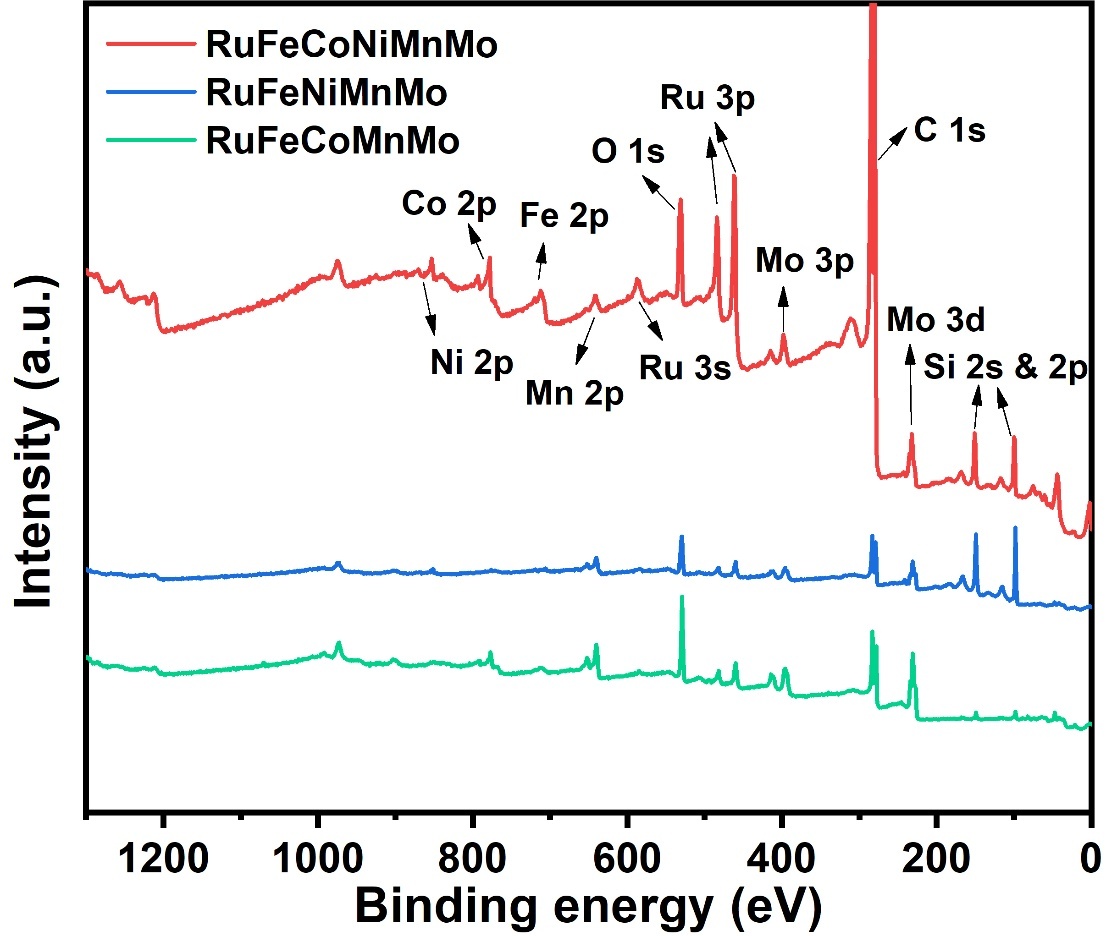


**Figure S24.** XPS full spectra of RuFeCoNiMnMo, RuFeNiMnMo, and RuFeCoMnMo HEAs.


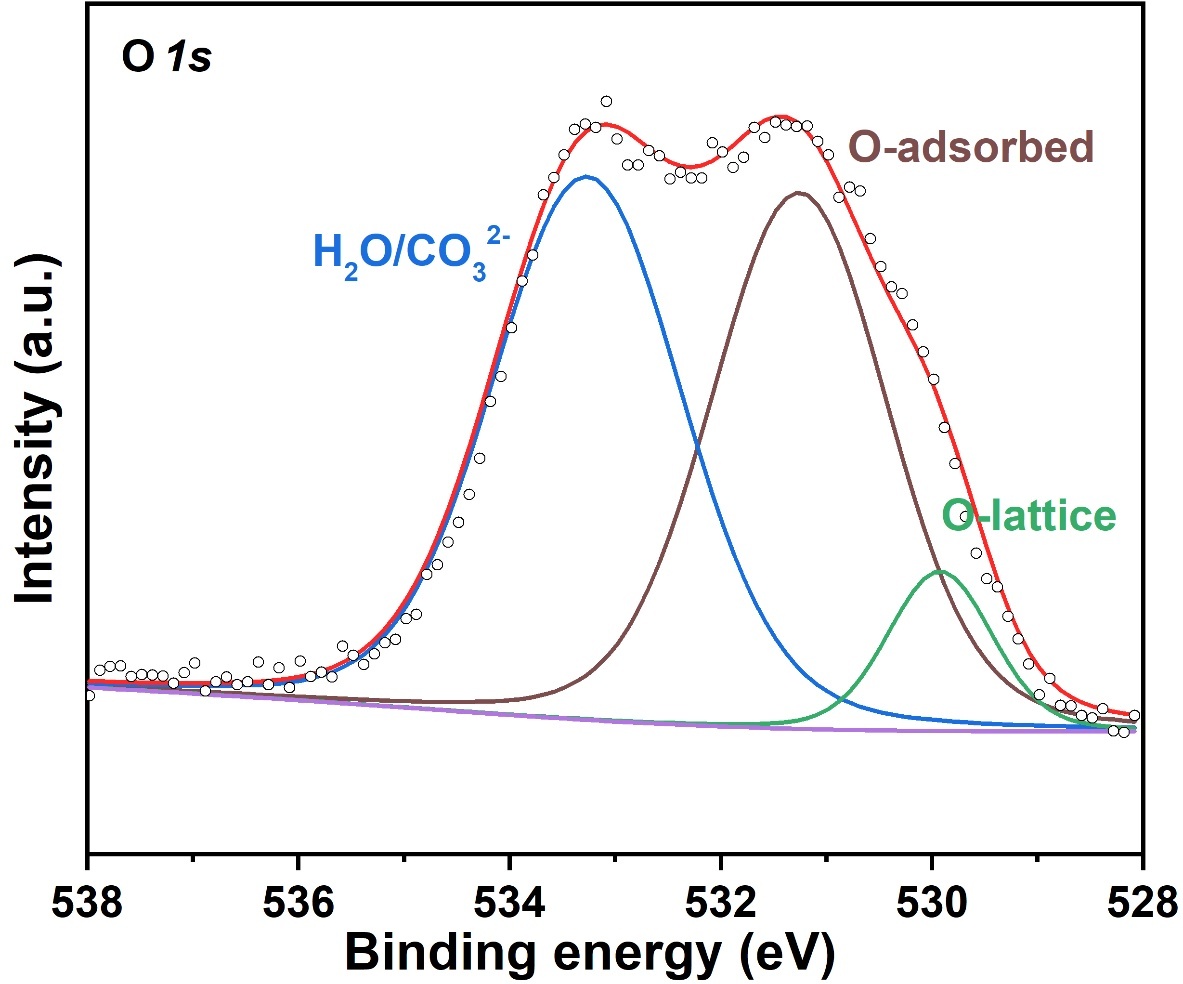


**Figure S25.** The O 1s XPS spectrum of RuFeCoNiMnMo HEAs.


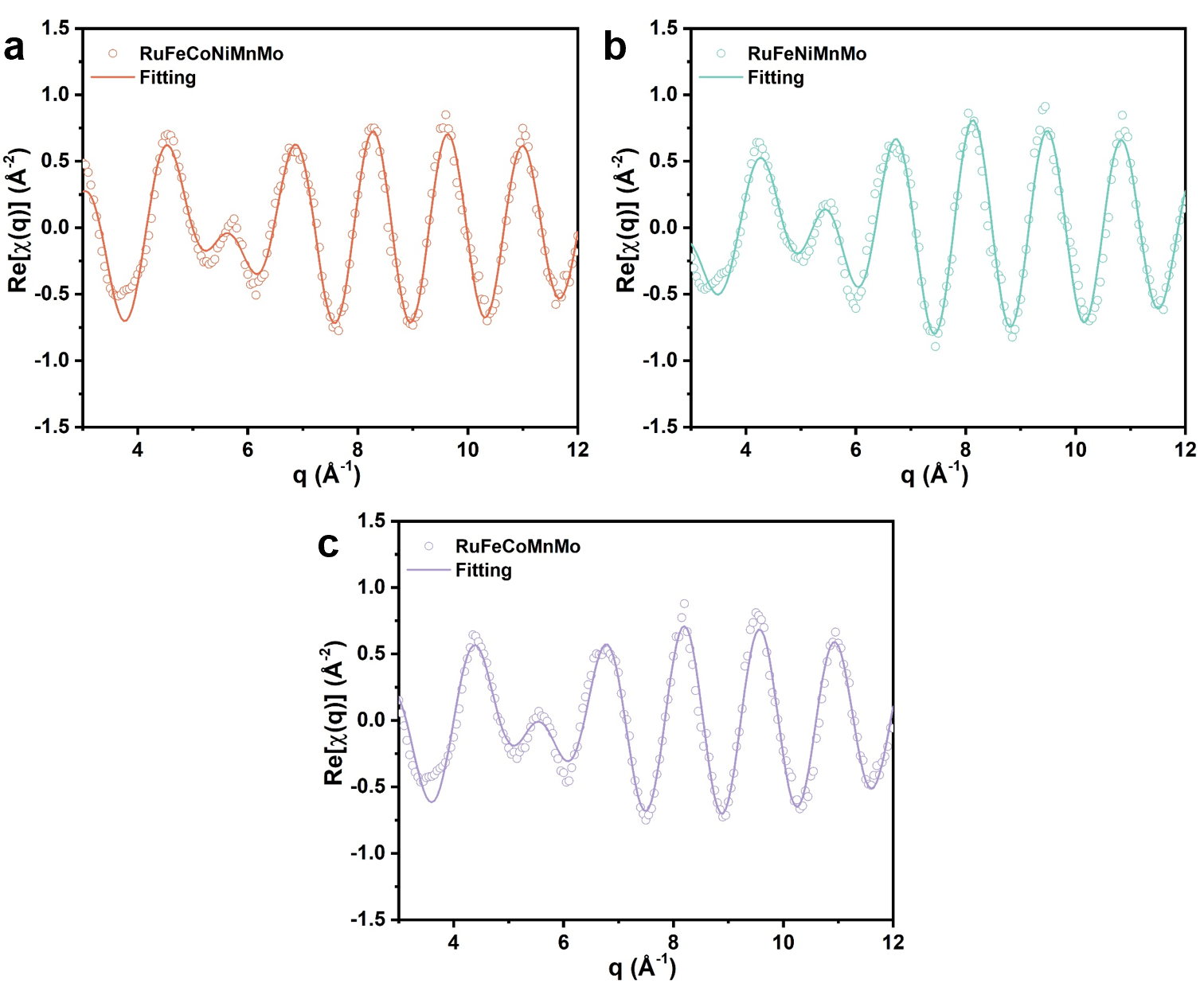


**Figure S26.** Inverse Fourier transform EXAFS fitting results of Ru for (a) RuFeCoNiMnMo, (b) RuFeNiMnMo, and (c) RuFeCoMnMo HEAs, respectively.

**
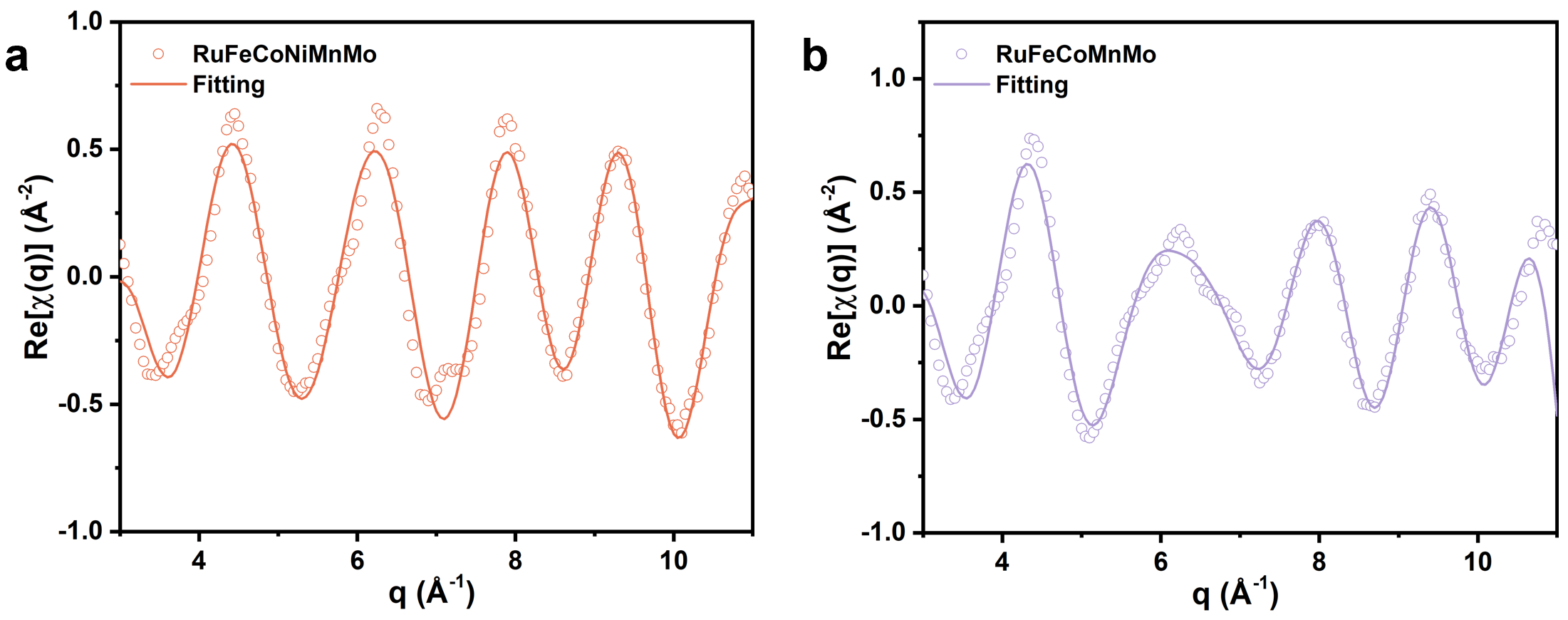
**

**Figure S27.** Inverse Fourier transform EXAFS fitting results of Co for (a) RuFeCoNiMnMo and (b) RuFeCoMnMo HEAs.

**
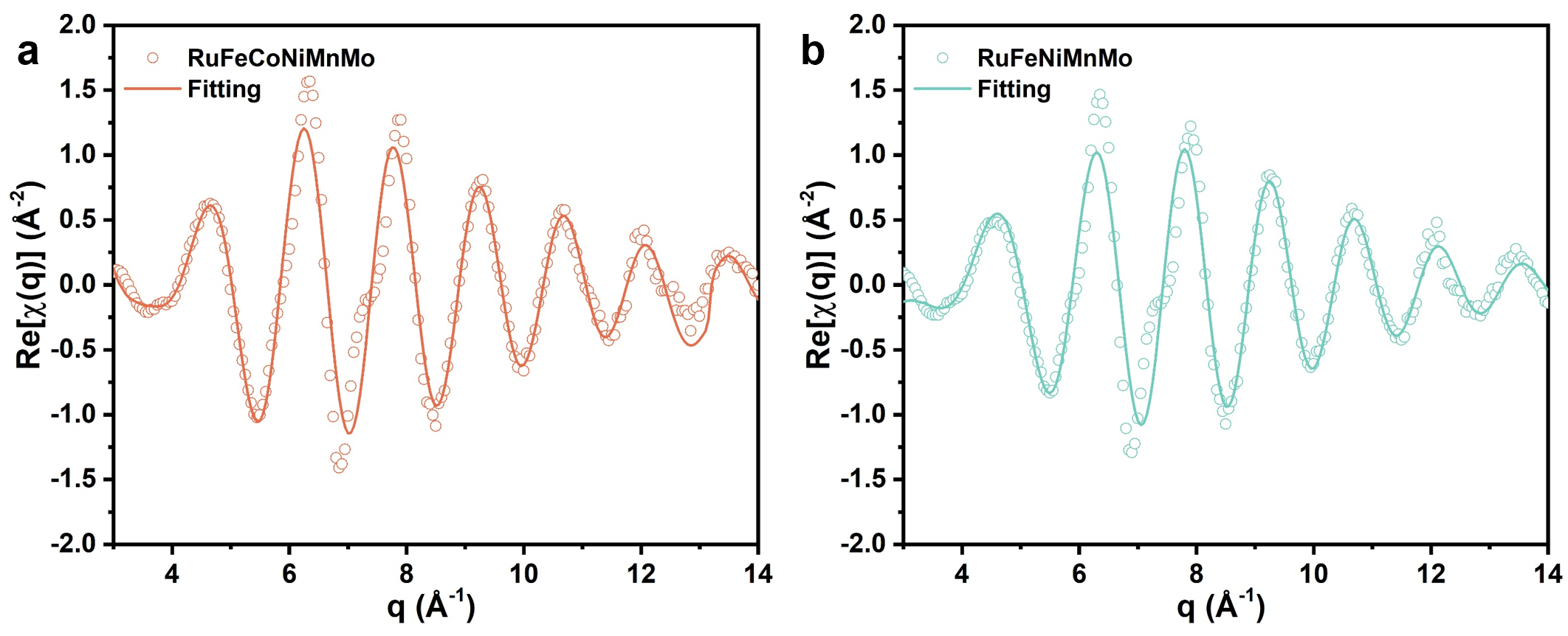
**

**Figure S28.** Inverse Fourier transform EXAFS fitting results of Ni for (a) RuFeCoNiMnMo and (b) RuFeNiMnMo HEAs.


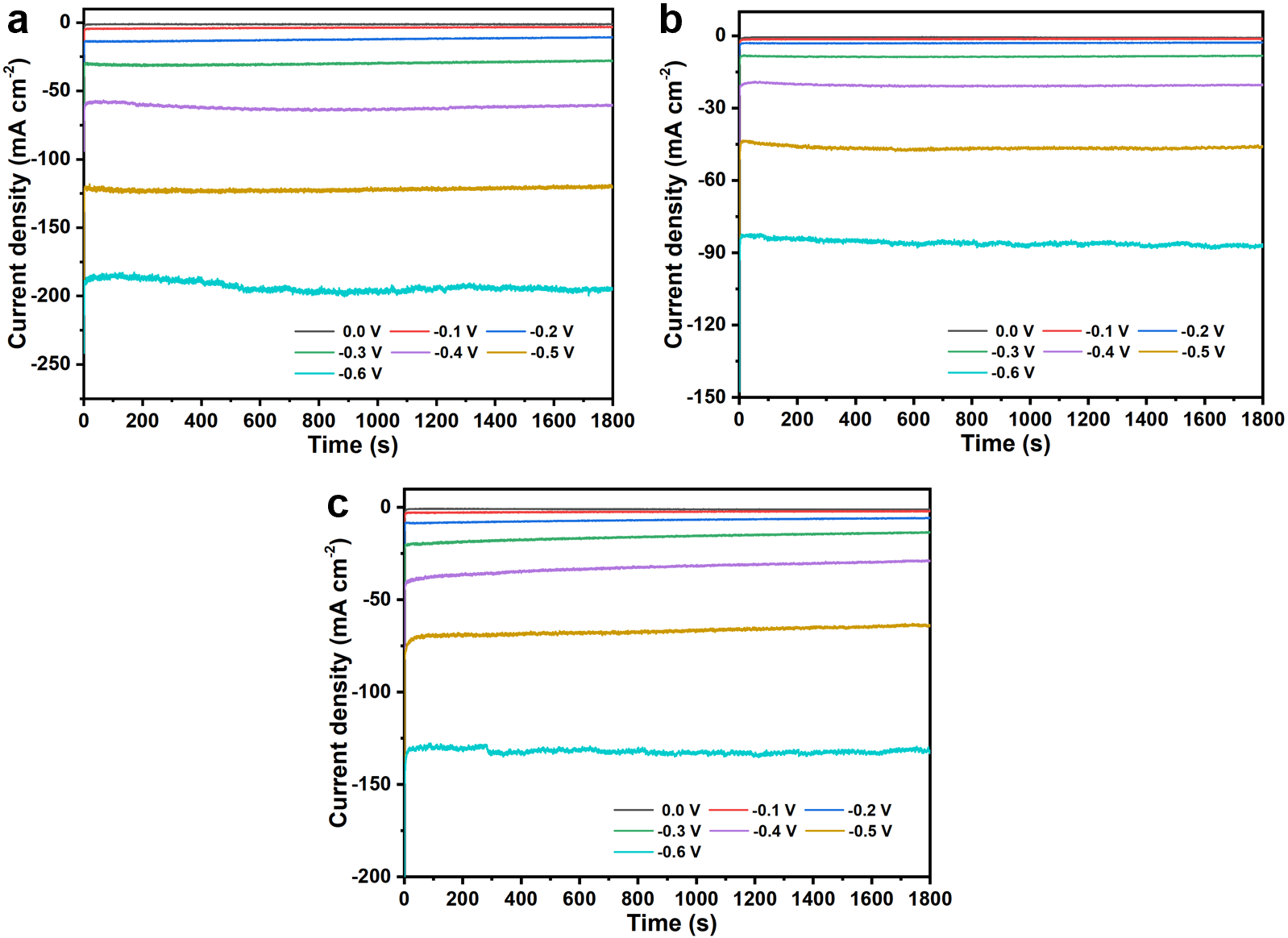


**Figure S29.** The chronoamperometric curves for (a) RuFeCoNiMnMo, (b) RuFeNiMnMo, and (c) RuFeCoMnMo HEAs, respectively, at various potentials (vs. RHE).

**
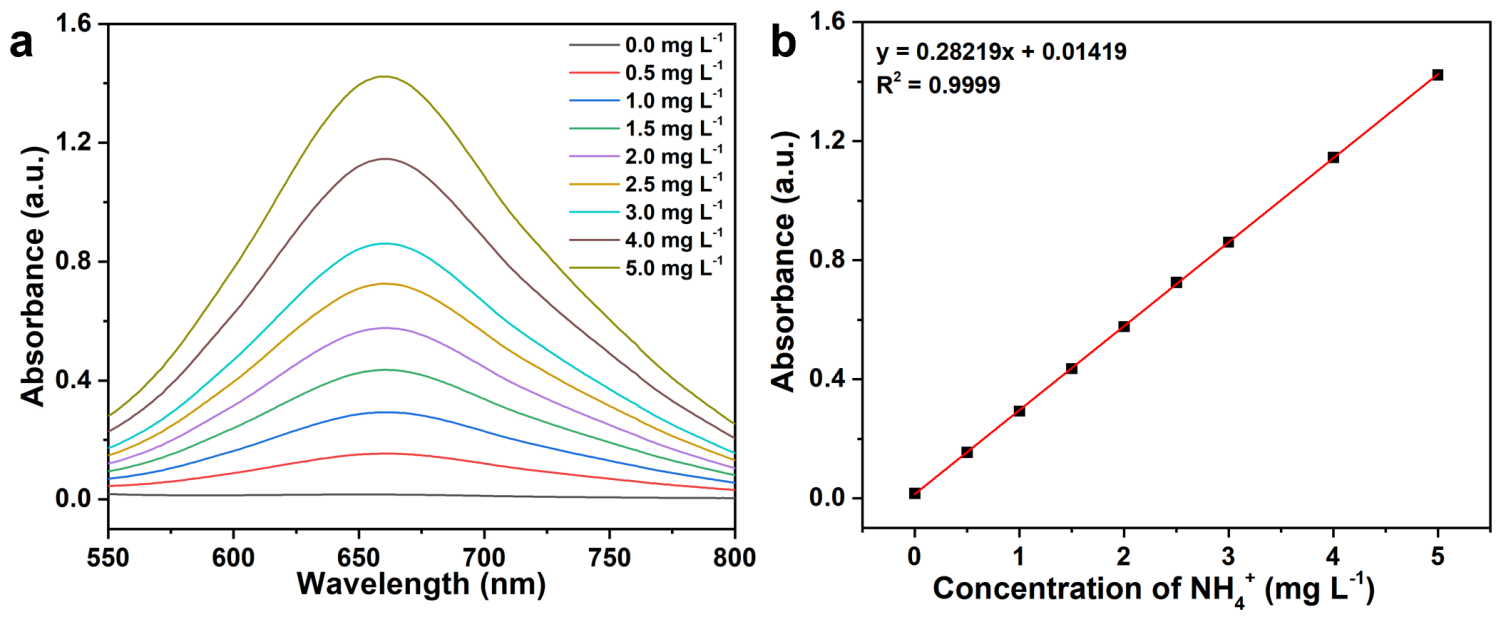
**

**Figure S30.** The UV-vis calibration curves of NH_3_ using different concentrations of NH_4_Cl solutions as standards. (a) UV-vis curves. (b) The linear fitting result.

**
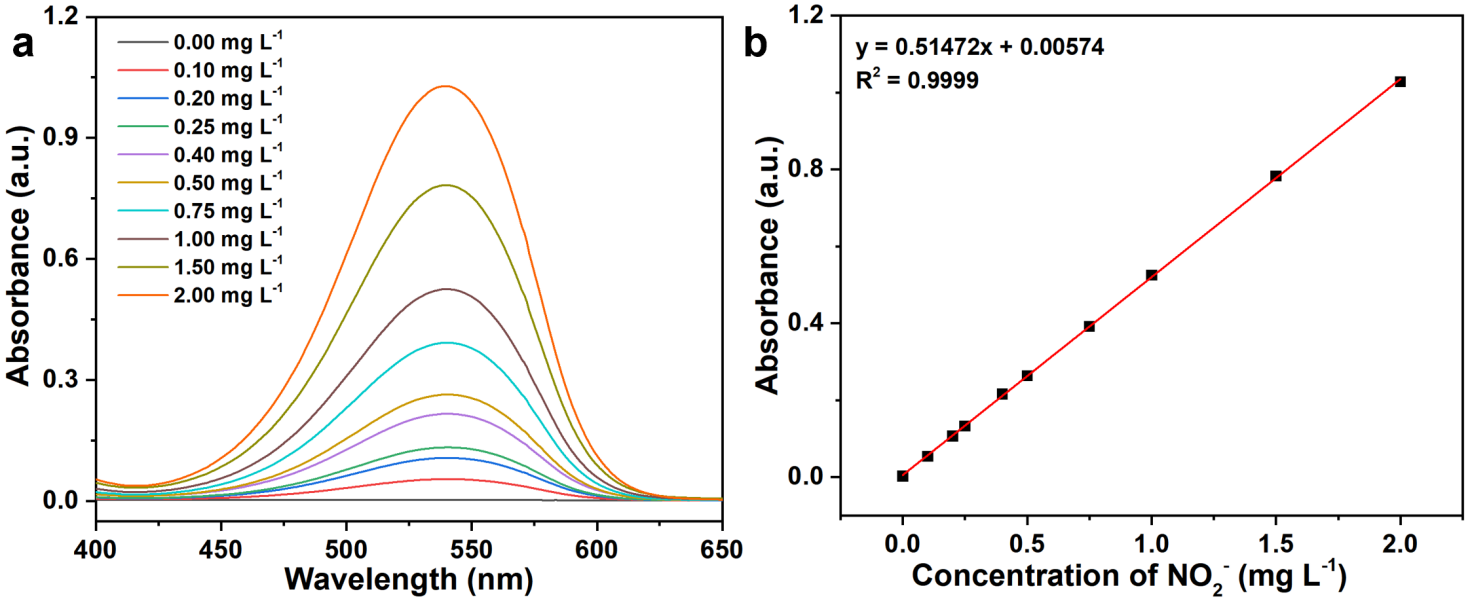
**

**Figure S31.** The UV-vis calibration curves of NO_2_^-^ using different concentrations of KNO_2_ solutions as standards. (a) UV-vis curves. (b) The linear fitting result.

**
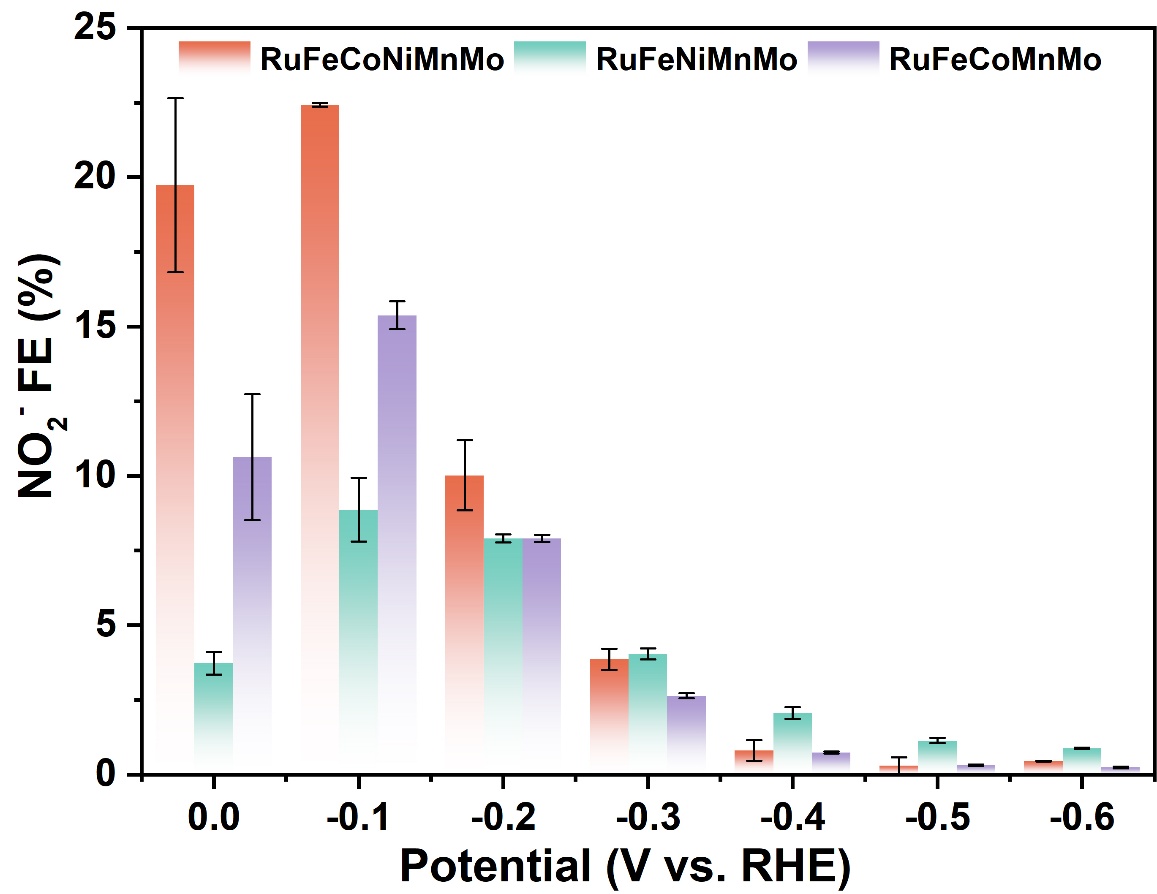
**

**Figure S32.** The NO_2_^-^ FE for RuFeCoNiMnMo, RuFeNiMnMo, and RuFeCoMnMo HEAs, respectively, at various potentials.

**
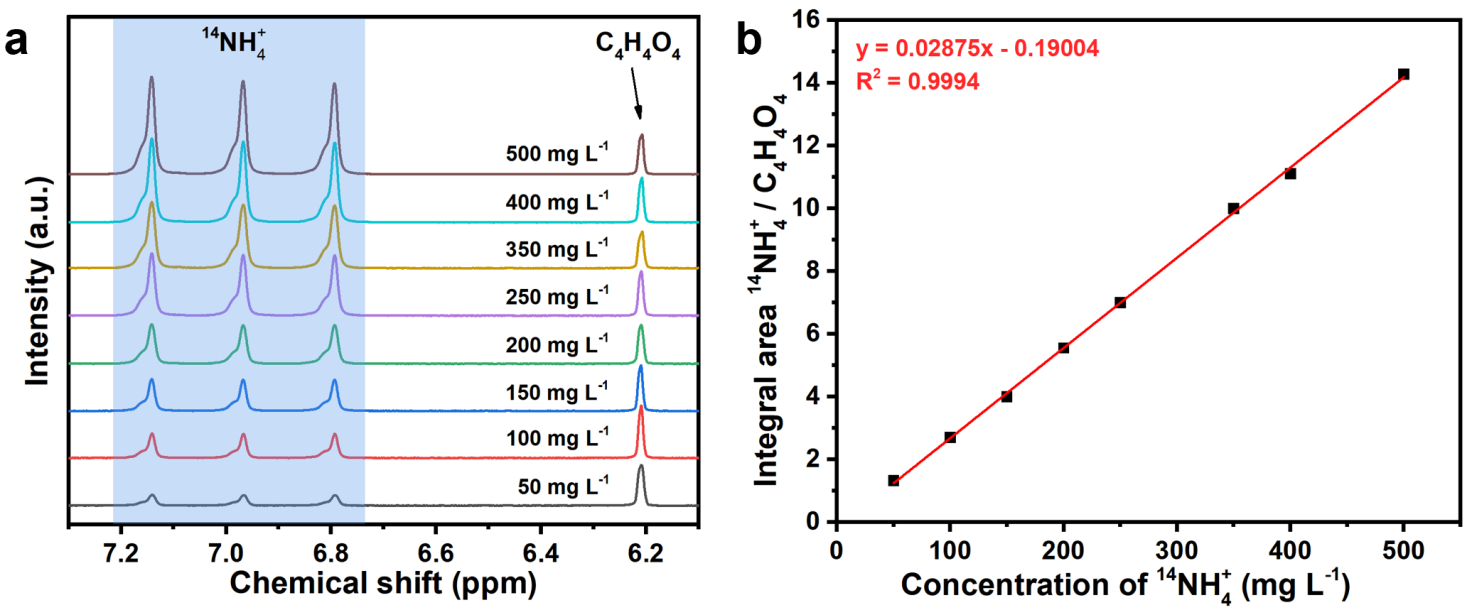
**

**Figure S33.** The NMR calibration curves of NH_4_^+^ using C_4_H_4_O_4_ as internal standard. (a) ^1^H NMR spectra with different ^14^NH_4_Cl concentrations. (b) The linear fitting result.

**
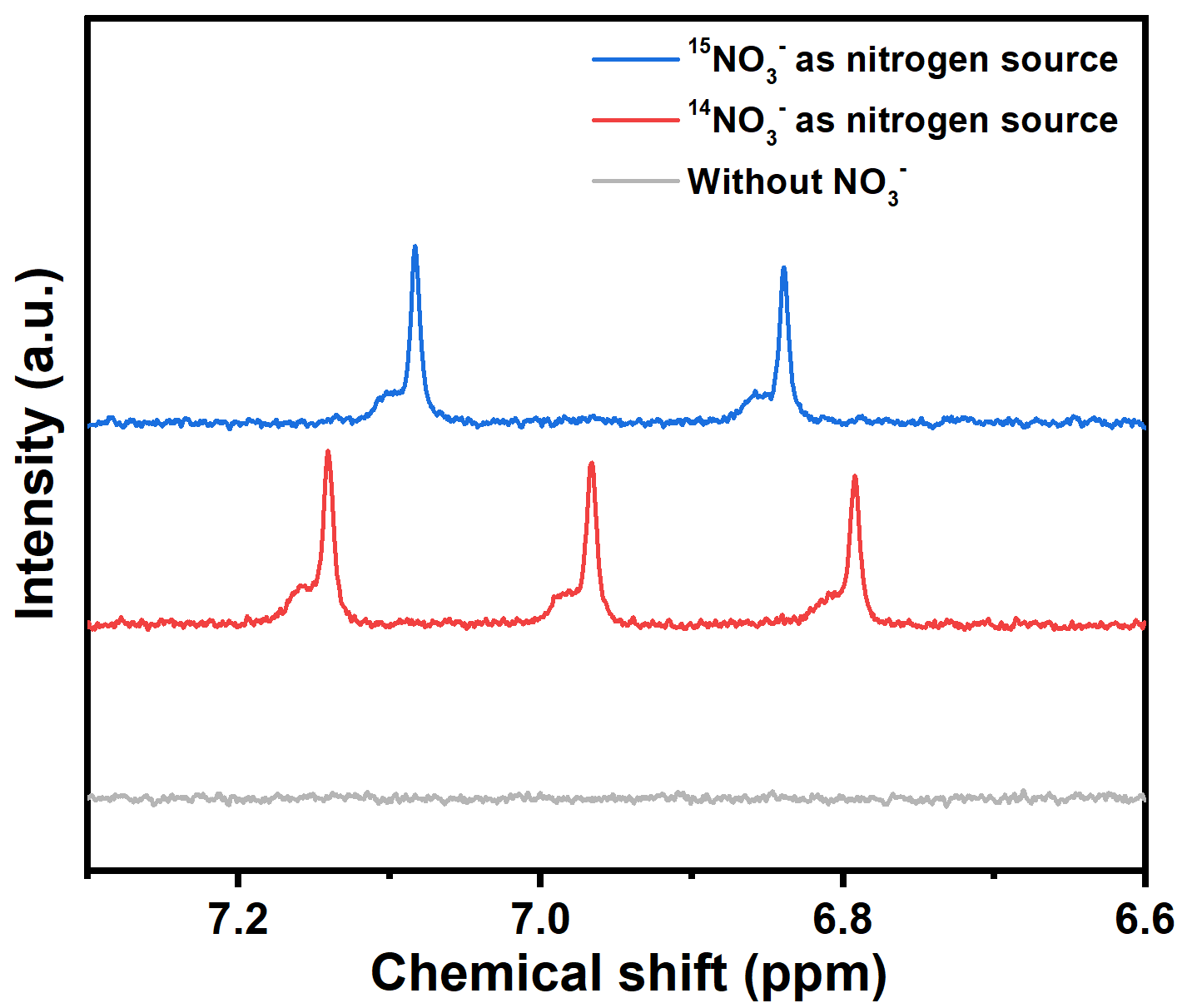
**

**Figure S34.** NMR spectra of the electrolytes after electrolysis at -0.30 V (vs. RHE) without or with K^14^NO_3_ and K^15^NO_3_ as the feeding nitrogen sources.

**
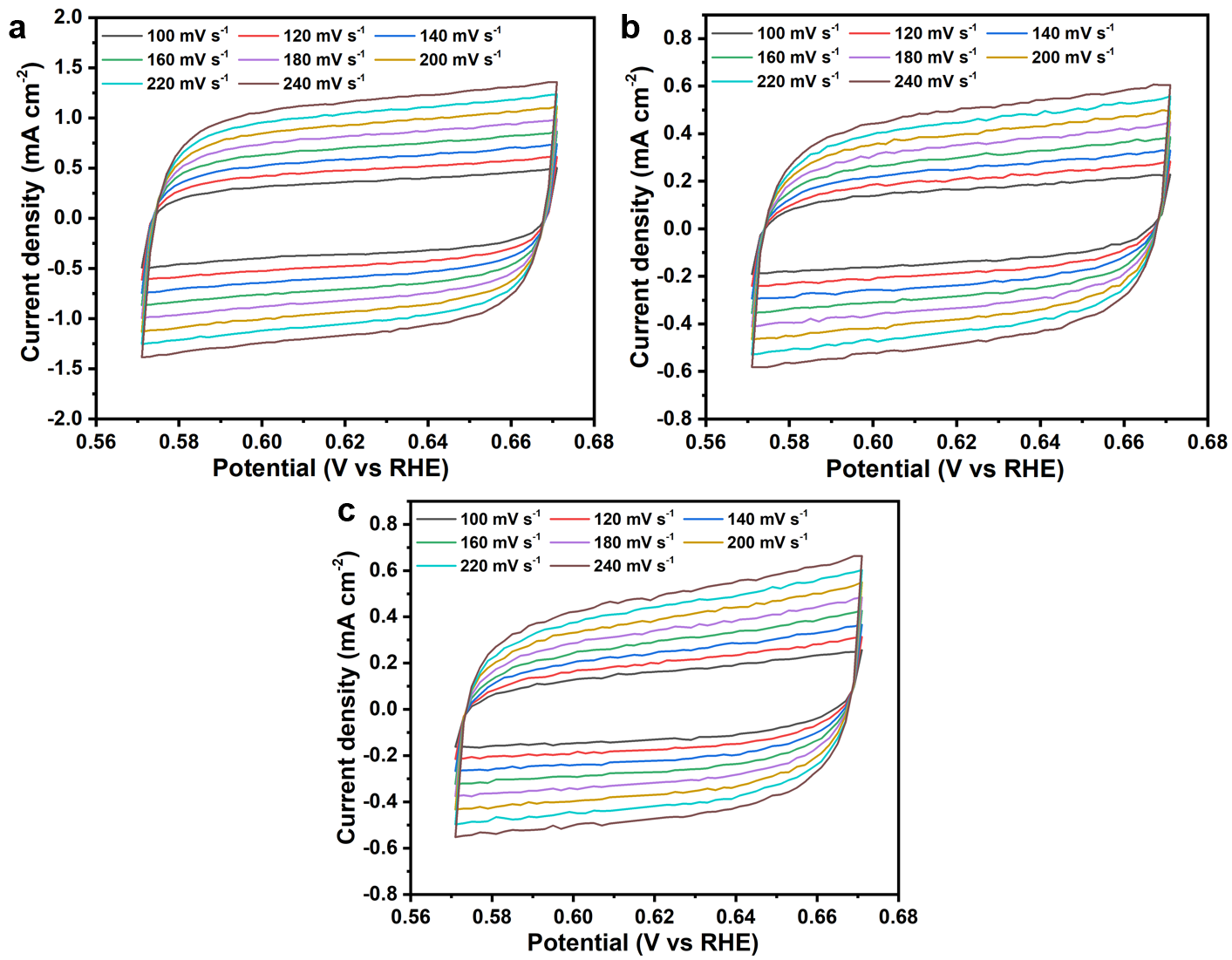
**

**Figure S35.** The CV curves for (a) RuFeCoNiMnMo, (b) RuFeNiMnMo, and (c) RuFeCoMnMo HEAs, respectively, at the sweep rates of 100, 120, 140, 160, 180, 200, 220, and 240 mV s^-1^. The ECSA was calculated as follows:

$$\mathrm{ECSA}\left( \mathrm{RuFeCoNiMnMo} \right)=\frac{11.4 mF \mathrm{cm}^{-2}}{40 \mu m \mathrm{cm}^{-2}\mathrm{per}\mathrm{cm}^{2}}=285 \mathrm{cm}^{2}$$

$$\mathrm{ECSA}\left( \mathrm{RuFeNiMnMo} \right)=\frac{4.8 mF \mathrm{cm}^{-2}}{40 \mu m \mathrm{cm}^{-2}\mathrm{per}\mathrm{cm}^{2}}=120 \mathrm{cm}^{2}$$

$$\mathrm{ECSA}\left( \mathrm{RuFeCoMnMo} \right)=\frac{4.8 mF \mathrm{cm}^{-2}}{40 \mu m \mathrm{cm}^{-2}\mathrm{per}\mathrm{cm}^{2}}=120 \mathrm{cm}^{2}$$

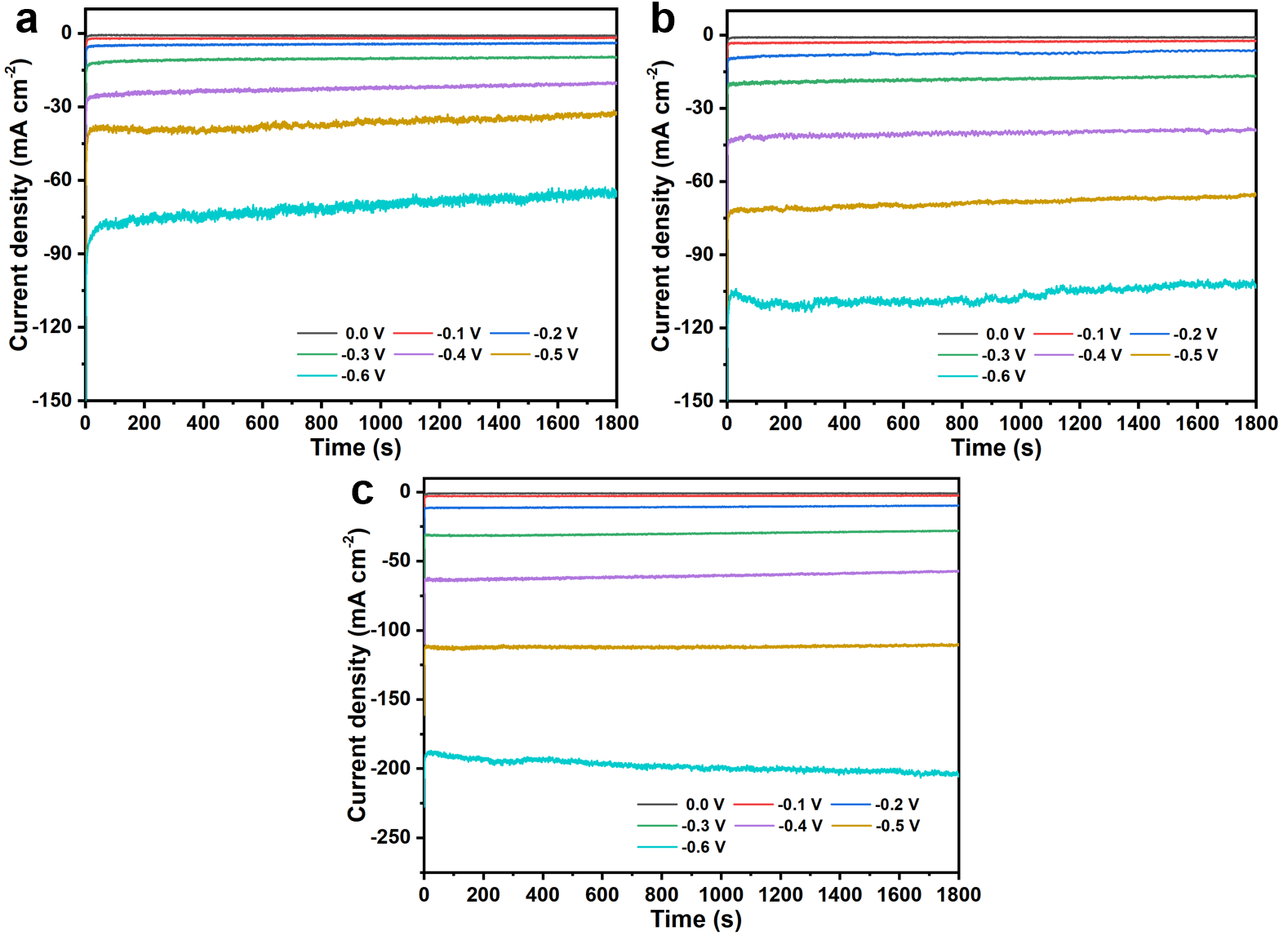


**Figure S36.** The chronoamperometric curves for RuFeCoNiMnMo at various potentials with different concentrations of KNO_3_: (a) 0.01 M, (b) 0.05 M, and (c) 0.20 M.


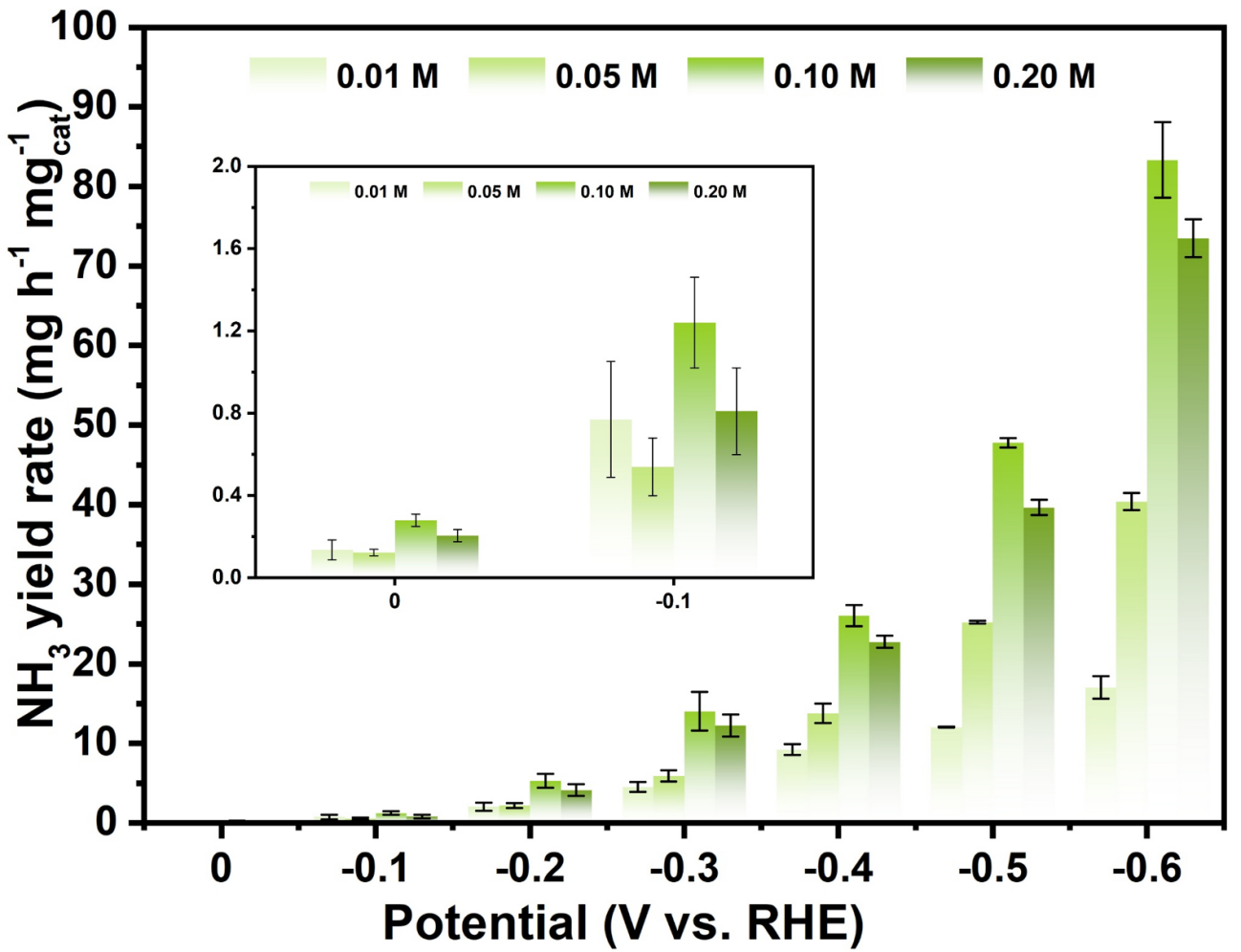


**Figure S37.** The NH_3_ yield rate for RuFeCoNiMnMo HEAs with different concentrations of KNO_3_ (0.01 M, 0.05 M, 0.10 M, and 0.20 M) at various potentials.


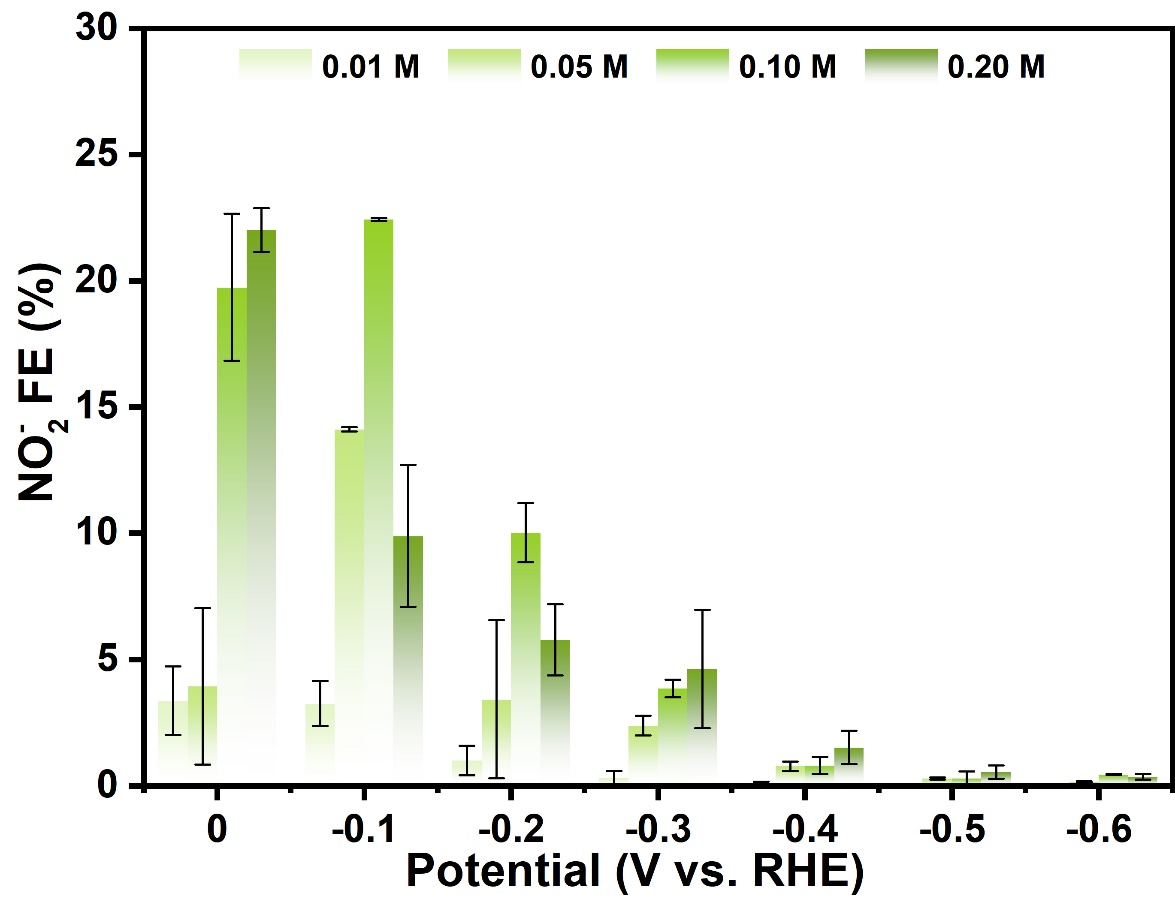


**Figure S38.** The NO_2_^-^ FE for RuFeCoNiMnMo HEAs with different concentrations of KNO_3_ (0.01 M, 0.05 M, 0.10 M, and 0.20 M) at various potentials.


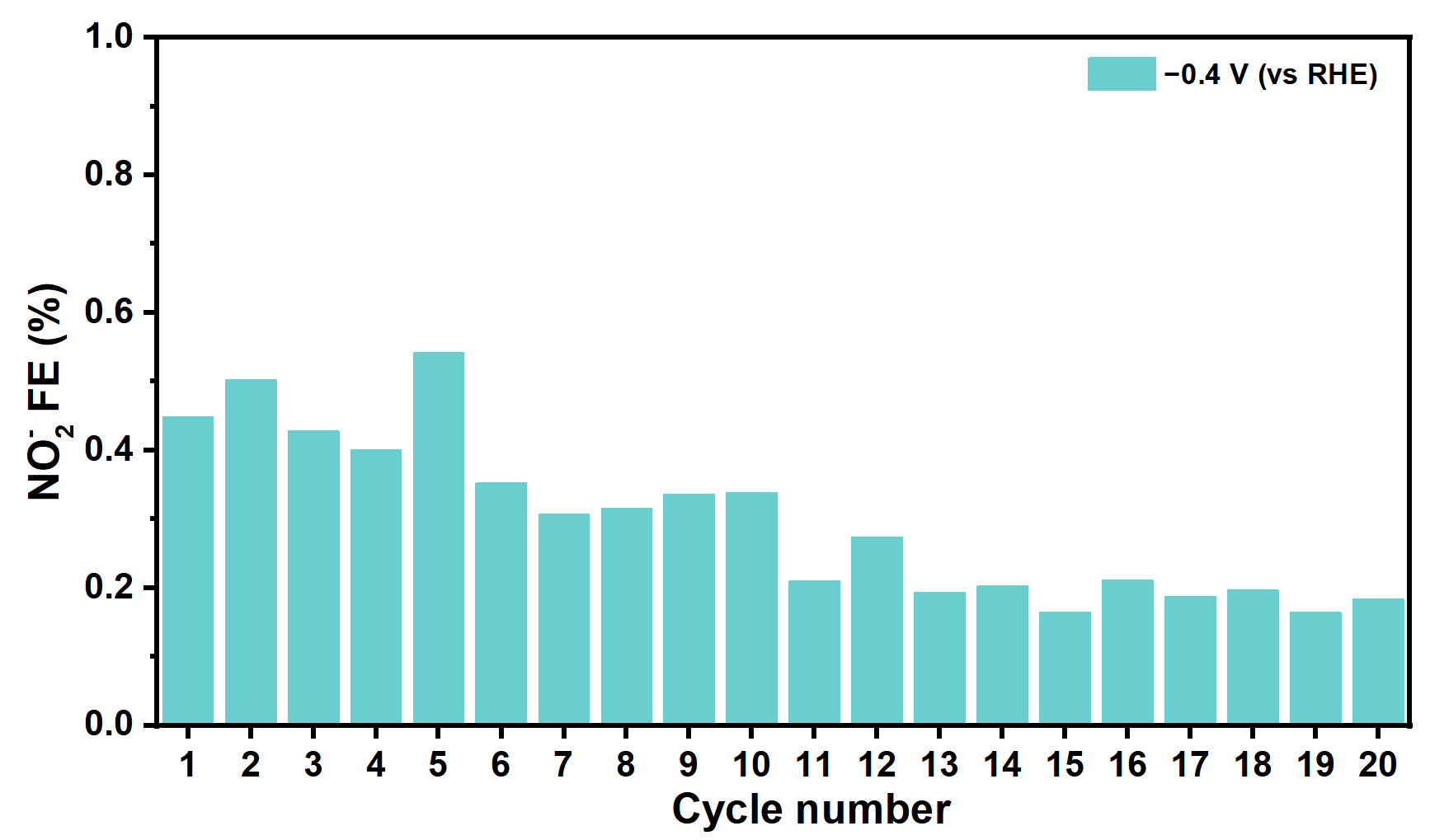


**Figure S39.** The NO_2_^-^ FE for RuFeCoNiMnMo HEAs during the consecutive electrolysis of 20 cycles at -0.4 V (vs. RHE).


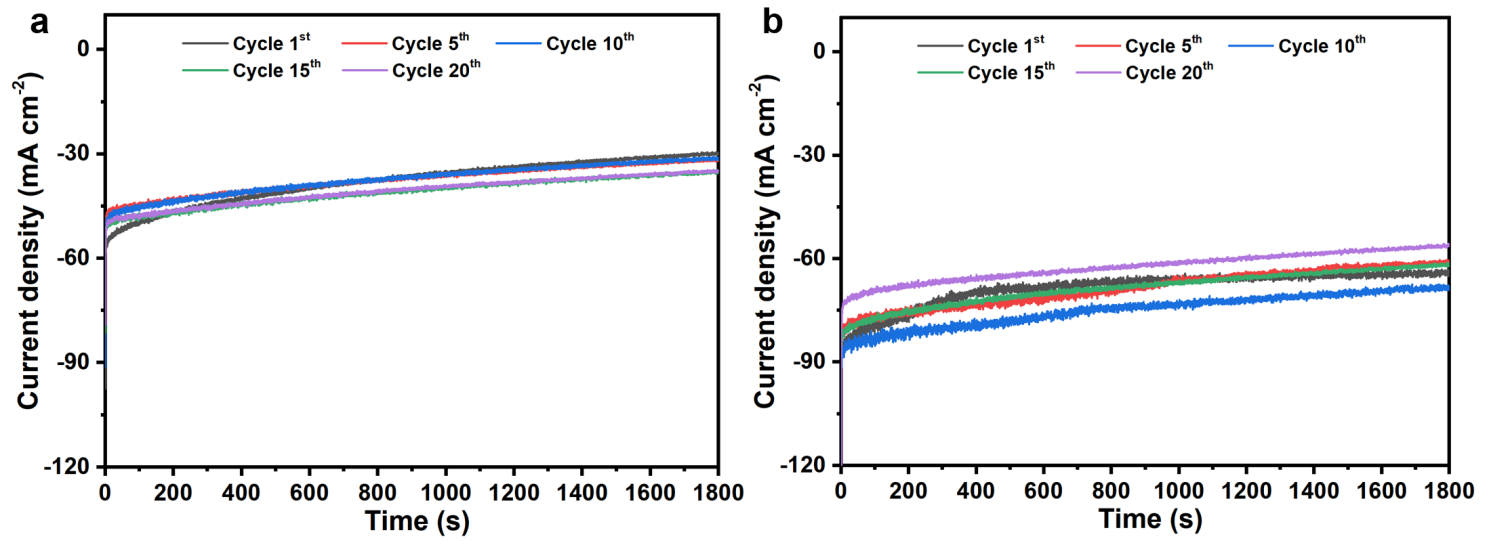


**Figure S40.** The chronoamperometric curves for RuFeCoNiMnMo HEAs during the consecutive electrolysis of 20 cycles at (a) -0.3 and (b) -0.4 V (vs. RHE).


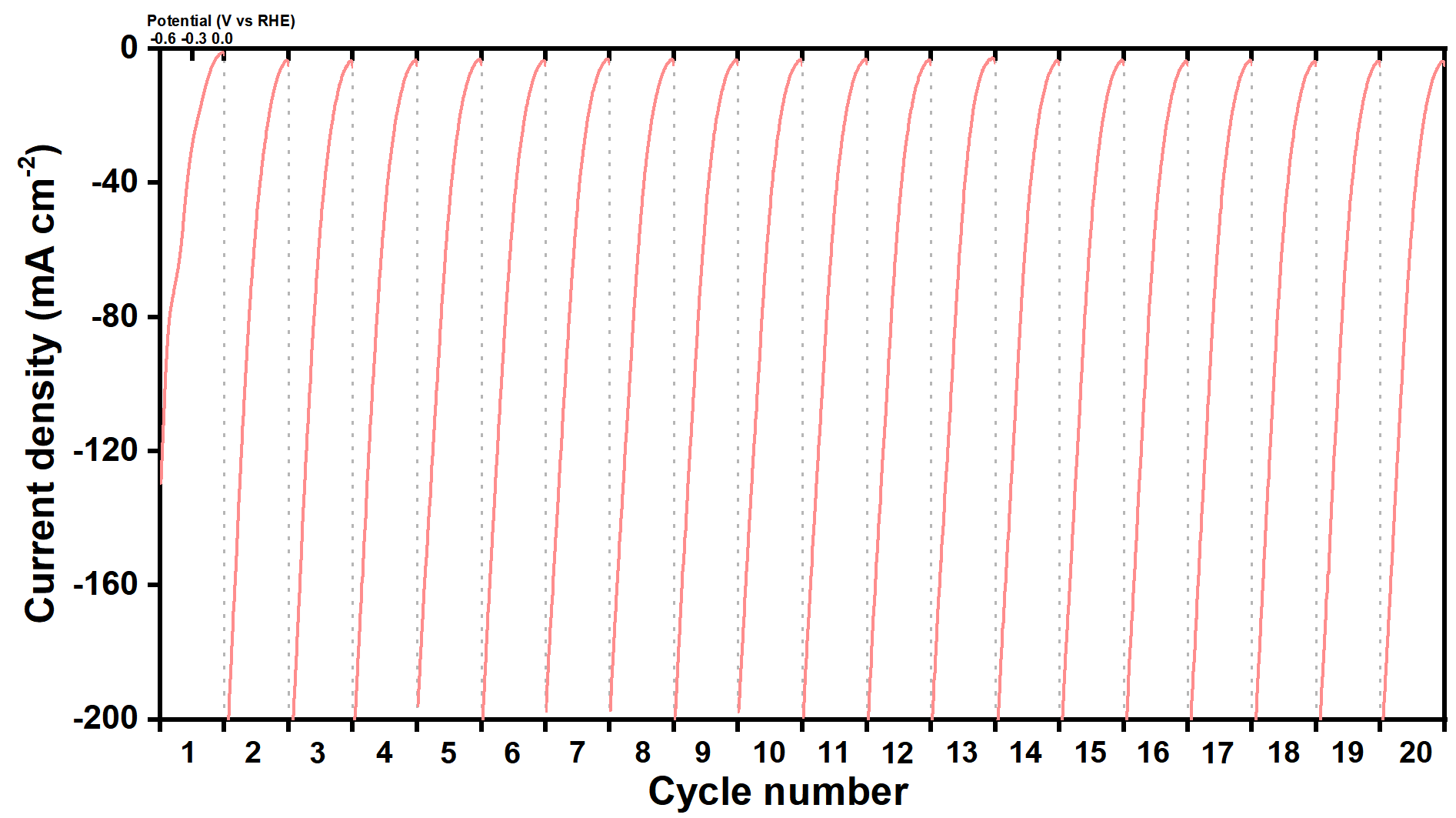


**Figure S41.** The LSV curves for RuFeCoNiMnMo HEAs after every consecutive electrolysis.


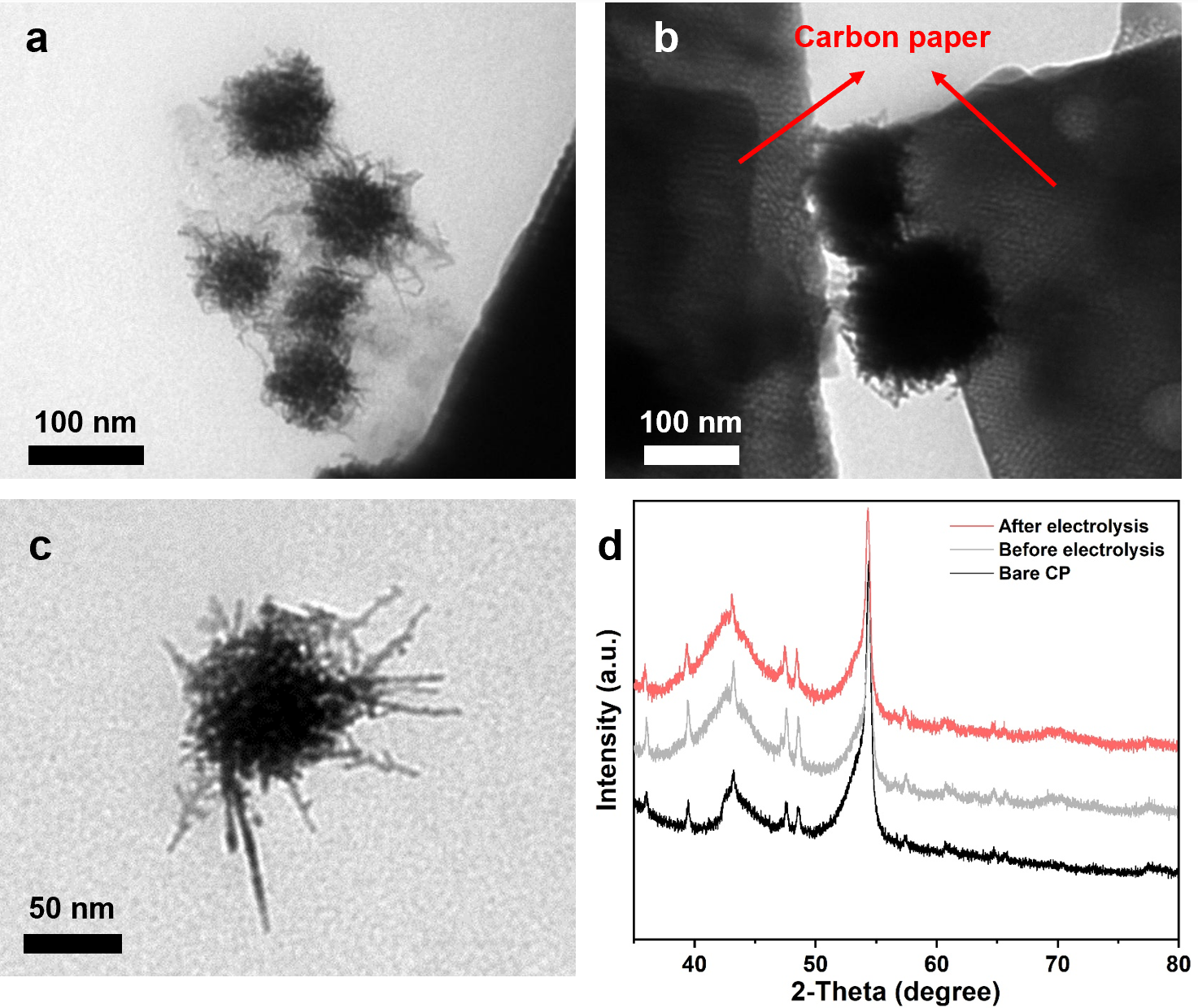


**Figure S42.** (a-c) TEM images, and (d) XRD patterns of RuFeCoNiMnMo HEAs after the electrolysis test. CP: carbon paper.


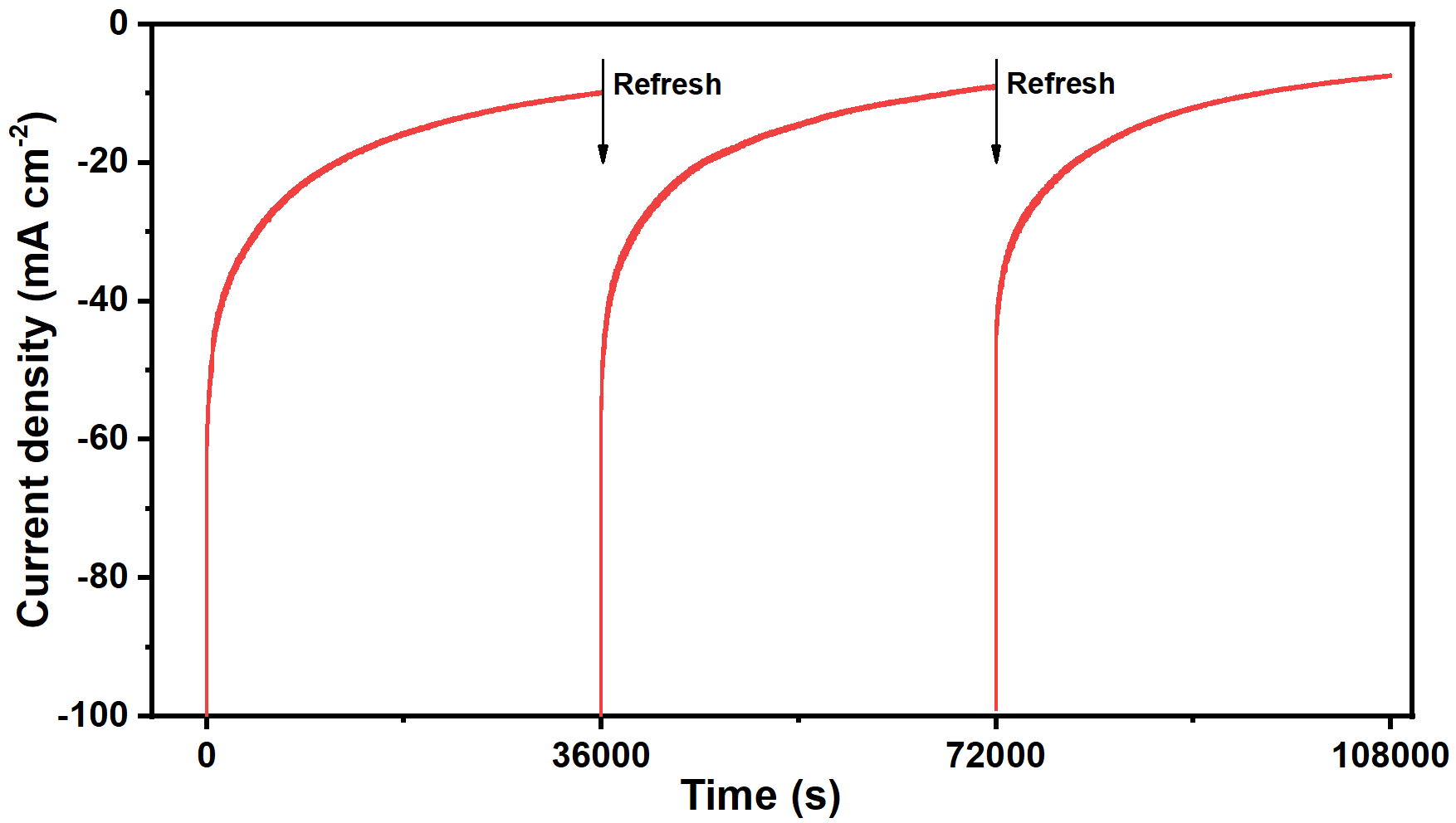


**Figure S43.** The chronoamperometric curves for RuFeCoNiMnMo HEAs during the long-term electrolysis (refresh the electrolyte every 10 h).


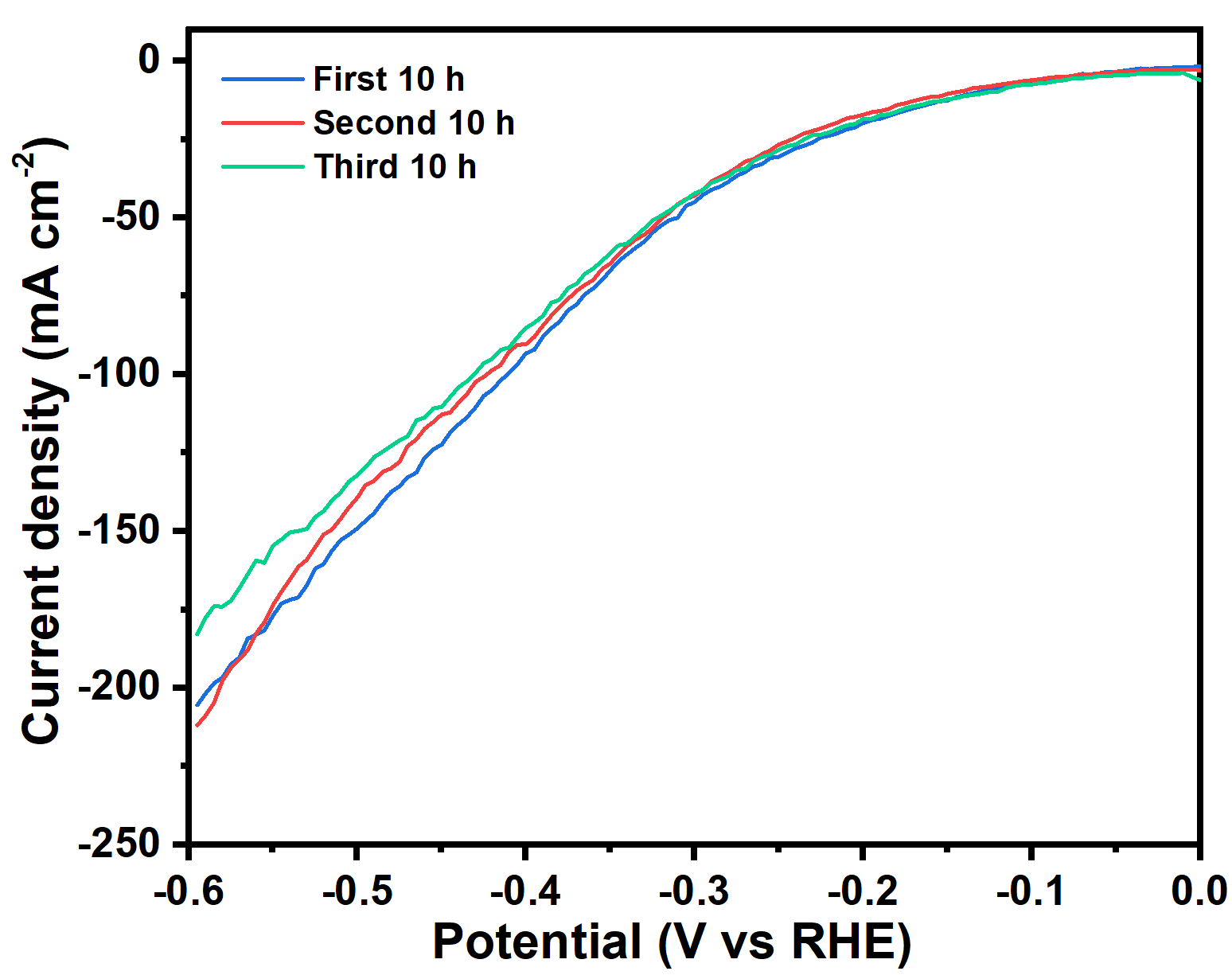


**Figure S44.** The LSV curves for RuFeCoNiMnMo HEAs after the long-term electrolysis.


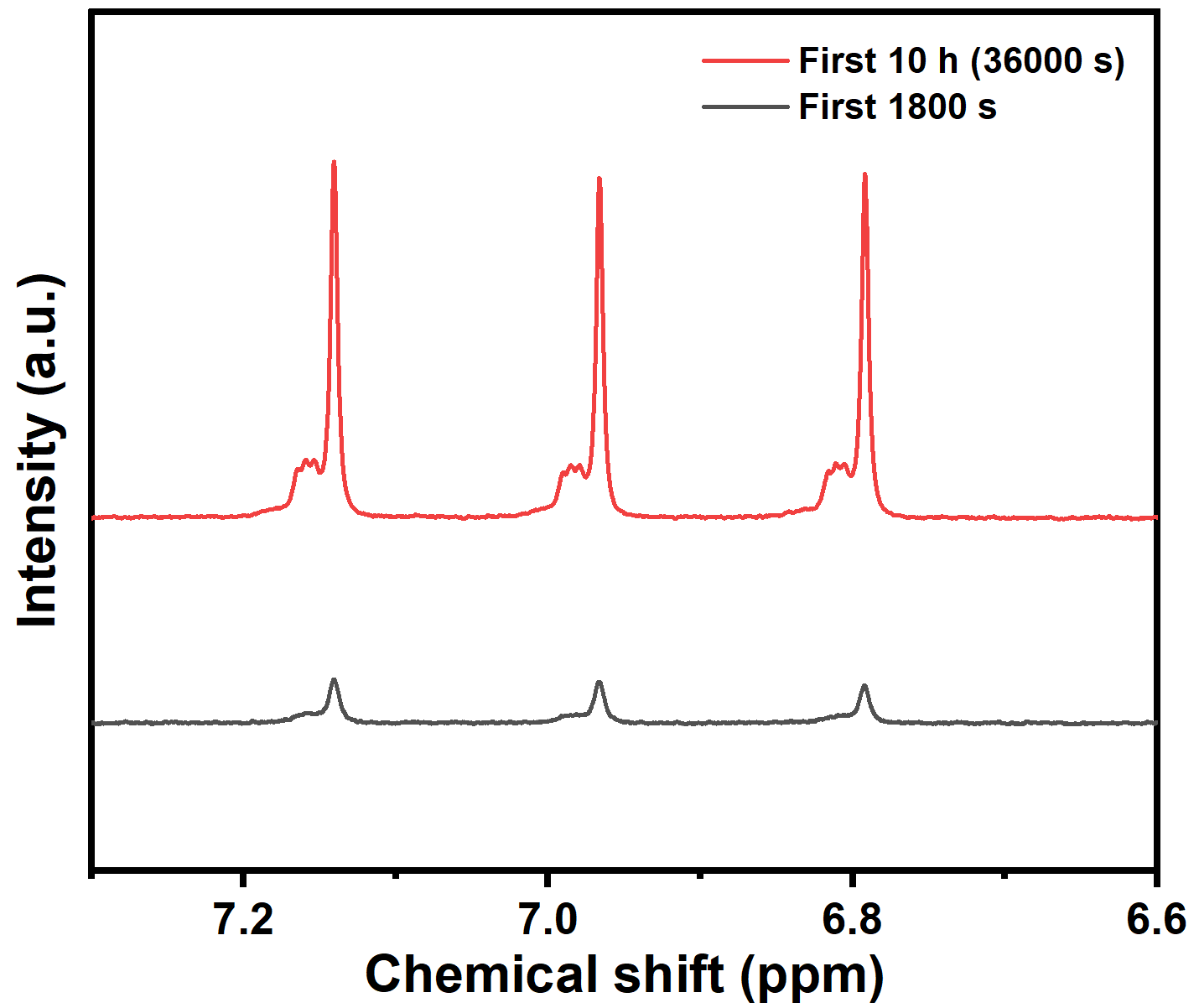


**Figure S45.** The NMR spectra for RuFeCoNiMnMo HEAs after the long-term electrolysis.


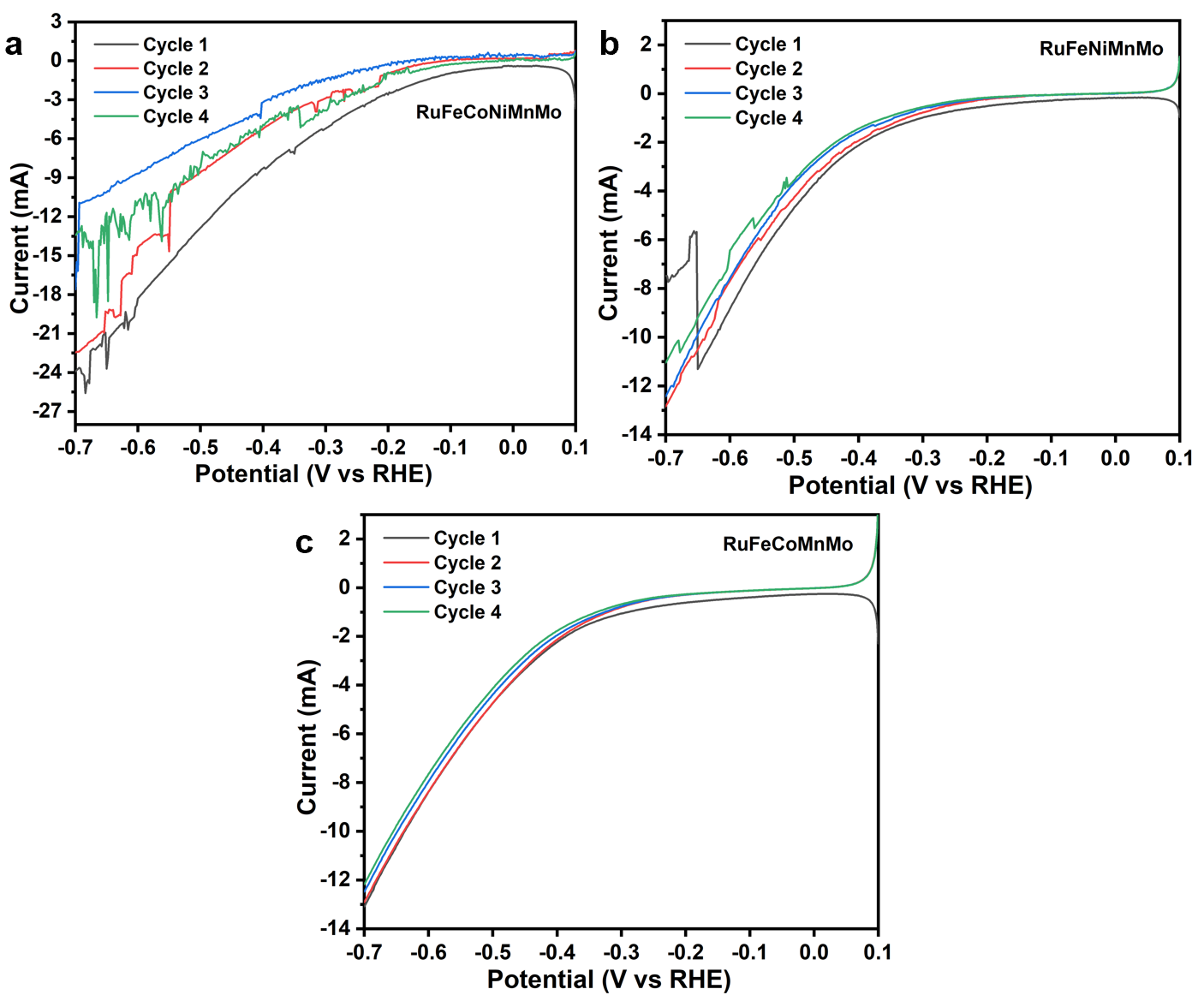


**Figure S46.** Four LSV scan cycles (0.1 to -0.7 V (vs. RHE)) during in-situ DEMS test for (a) RuFeCoNiMnMo, (b) RuFeNiMnMo, and (c) RuFeCoMnMo HEAs, respectively.


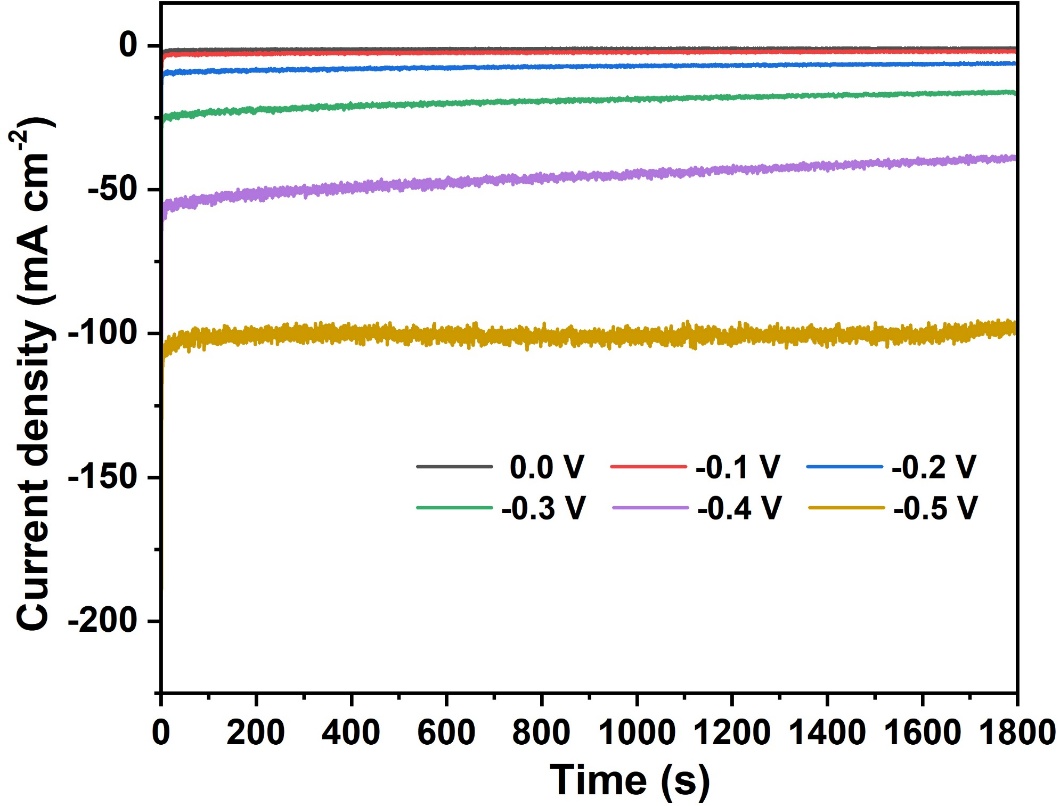


**Figure S47.** The chronoamperometric curves for RuFeCoNiMnMo HEAs at 0.2 M TBA.


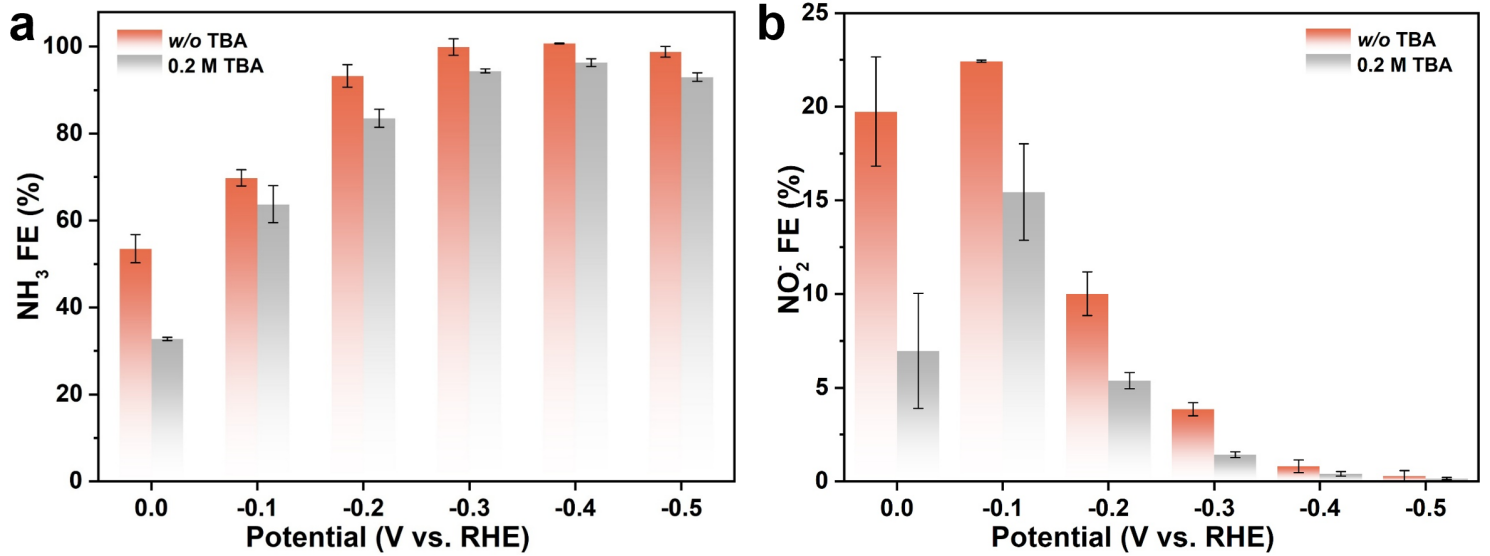


**Figure S48.** (a) The NH_3_ FE, and (b) NO_2_^-^ FE for RuFeCoNiMnMo HEAs without (*w/o*) TBA and with 0.2 M TBA, respectively.

**Note S1.** Calculations of three prerequisites (the mixing entropy, mixing enthalpy, and atomic radius difference) for the formation of high entropy alloys.

1. **Mixing entropy (Δ*S*_mix_) could be calculated by:**

$${\Delta S}_{mix}=-R\sum_{i=1}^{n} C_{i}\ln C_{i}$$

where *C_i_* is the atomic percentage of the i^th^ element, and *R* is the molar gas constant.

**Table S1.** Calculation of Δ*S*_mix_ of RuFeCoNiMnMo, RuFeNiMnMo, and RuFeCoMnMo HEAs.

| **Element** | **RuFeCoNiMnMo** | | **RuFeNiMnMo** | | **RuFeCoMnMo** | |
| --- | --- | --- | --- | --- | --- | --- |
|  | ***C_i_*** | ***C_i_*ln*C_i_*** | ***C_i_*** | ***C_i_*ln*C_i_*** | ***C_i_*** | ***C_i_*ln*C_i_*** |
| Mo | 0.135 | -0.27 | 0.196 | -0.32 | 0.216 | -0.33 |
| Ru | 0.284 | -0.36 | 0.359 | -0.37 | 0.315 | -0.36 |
| Mn | 0.145 | -0.28 | 0.187 | -0.31 | 0.185 | -0.31 |
| Fe | 0.147 | -0.28 | 0.132 | -0.27 | 0.116 | -0.25 |
| Co | 0.161 | -0.29 | / | / | 0.168 | -0.30 |
| Ni | 0.128 | -0.26 | 0.126 | -0.26 | / | / |
| RuFeCoNiMnMo | Δ*S*_mix_ = 1.74*R* | | Δ*S*_mix_ = 1.53*R* | | Δ*S*_mix_ = 1.55*R* | |

1. **Mixing enthalpy (Δ*H*_mix_) could be calculated by:**

$${\Delta H}_{mix}=4\sum_{i=1, i\neq j}^{n} {{\Delta H}_{ij}^{mix}C}_{i}C_{j}$$

where Δ*H*mix ijis the mixing enthalpy of binary liquid ij alloys, and *C_i_* or *C_j_* is the atomic percentage of the i^th^ or j^th^ element.

**Table S2.** Calculation of Δ*H*_mix_ of RuFeCoNiMnMo, RuFeNiMnMo, and RuFeCoMnMo HEAs.

| **Binary alloy** | **RuFeCoNiMnMo** | | **RuFeNiMnMo** | | **RuFeCoMnMo** | |
| --- | --- | --- | --- | --- | --- | --- |
|  | **Δ*H*mix ij**  **(kJ mol^-1^)** | **Δ*H*mix ij*C_i_C_j_*** | **Δ*H*mix ij**  **(kJ mol^-1^)** | **Δ*H*mix ij*C_i_C_j_*** | **Δ*H*mix ij**  **(kJ mol^-1^)** | **Δ*H*mix ij*C_i_C_j_*** |
| RuFe | -5 | -0.21 | -5 | -0.24 | -5 | -0.18 |
| RuCo | -1 | -0.05 | / | / | -1 | -0.05 |
| RuNi | 0 | 0 | 0 | 0 | / | / |
| RuMn | -11 | -0.45 | -11 | -0.74 | -11 | -0.64 |
| RuMo | -14 | -0.54 | -14 | -0.99 | -14 | -0.95 |
| FeCo | -1 | -0.02 | / | / | -1 | -0.02 |
| FeNi | -2 | -0.04 | -2 | -0.03 | / | / |
| FeMn | 0 | 0 | 0 | 0 | 0 | 0 |
| FeMo | -2 | -0.04 | -2 | -0.05 | -2 | -0.05 |
| CoNi | 0 | 0 | / | / | / | / |
| CoMn | -5 | -0.12 | / | / | -5 | -0.16 |
| CoMo | -5 | -0.11 | / | / | -5 | -0.18 |
| NiMn | -8 | -0.15 | -8 | -0.19 | / | / |
| NiMo | -7 | -0.12 | -7 | -0.17 | / | / |
| MnMo | 5 | 0.10 | 5 | 0.18 | 5 | 0.20 |
|  | Δ*H*_mix_ = -7.0 kJ mol^-1^ | | Δ*H*_mix_ = -8.9 kJ mol^-1^ | | Δ*H*_mix_ = -8.1 kJ mol^-1^ | |

1. **Atomic radius difference (*δ*) could be calculated by:**

$$\bar{r}=\sum_{i=1}^{n} C_{i}r_{i}$$

$$\delta=\sqrt{\sum_{i=1}^{n} {C_{i}(1-\frac{r_{i}}{\bar{r}})}^{2}}$$

where $\bar{r}$ is the average atomic radius of HEAs.

**Table S3.** Calculation of *δ* for RuFeCoNiMnMo, RuFeNiMnMo, and RuFeCoMnMo HEAs.

| **Element** | **RuFeCoNiMnMo** | | **RuFeNiMnMo** | | **RuFeCoMnMo** | |
| --- | --- | --- | --- | --- | --- | --- |
|  | ***r_i_* (pm)** | ${\boldsymbol{C}_{\boldsymbol{i}}\boldsymbol{(1-}\frac{\boldsymbol{r}_{\boldsymbol{i}}}{\bar{\boldsymbol{r}}}\boldsymbol{)}}^{\boldsymbol{2}}$ | ***r_i_* (pm)** | ${\boldsymbol{C}_{\boldsymbol{i}}\boldsymbol{(1-}\frac{\boldsymbol{r}_{\boldsymbol{i}}}{\bar{\boldsymbol{r}}}\boldsymbol{)}}^{\boldsymbol{2}}$ | ***r_i_* (pm)** | ${\boldsymbol{C}_{\boldsymbol{i}}\boldsymbol{(1-}\frac{\boldsymbol{r}_{\boldsymbol{i}}}{\bar{\boldsymbol{r}}}\boldsymbol{)}}^{\boldsymbol{2}}$ |
| Mo | 130 | 0.00081827 | 130 | 0.00080510 | 130 | 0.00088967 |
| Ru | 125 | 0.00037625 | 125 | 0.00019264 | 125 | 0.00017025 |
| Mn | 117 | 0.00012990 | 117 | 0.00033488 | 117 | 0.00033008 |
| Fe | 117 | 0.00013169 | 117 | 0.00023639 | 117 | 0.00020697 |
| Co | 116 | 0.00023521 | / | / | 116 | 0.00042718 |
| Ni | 115 | 0.00027693 | 115 | 0.00043399 | / | / |
|  | $\bar{r}$= 120.61 | *δ* = 0.044365 | $\bar{r}$= 122.17 | *δ* = 0.044755 | $\bar{r}$= 122.16 | *δ* = 0.044991 |

According to the above calculation, the as-synthesized RuFeCoNiMnMo satisfies three prerequisites for the formation of HEAs: (a) Δ*S*_mix_ is 1.74*R*, higher than 1.5*R*, which is theoretically beneficial for forming a complete disorder state; (b) Δ*H*_mix_ is -7.0 kJ mol^-1^, between -11.6 to 3.2 kJ mol^-1^, which indicates that neither form intermetallic compounds nor cause elemental segregation; (c) *δ* is 4.4%, lower than 6.6%, which satisfies the formation for disordered solid solution.

**Table S4.** A summary of XPS results for RuFeCoNiMnMo, RuFeNiMnMo, and RuFeCoMnMo HEAs.

| **Element** | **Species** | **Binding Energy (eV)** | | |
| --- | --- | --- | --- | --- |
|  |  | **RuFeCoNiMnMo** | **RuFeNiMnMo** | **RuFeCoMnMo** |
| Ru | Ru^0^ 3*p*_3/2_ | 461.76 | 461.18 | 461.39 |
|  | Ru^0^ 3*p*_1/2_ | 484.04 | 483.45 | 483.64 |
| Fe | Fe^0^ 2*p*_3/2_ | 707.36 | 706.75 | 706.87 |
|  | Fe^2+^ 2*p*_3/2_ | 710.34 | 708.94 | 710.28 |
|  | Fe^3+^ 2*p*_3/2_ | 713.40 | 712.30 | 713.92 |
|  | *Sat.* | 716.97 | 716.90 | 718.86 |
|  | Fe^0^ 2*p*_1/2_ | 720.34 | 719.99 | 720.10 |
|  | Fe^2+^ 2*p*_1/2_ | 722.17 | 721.82 | 722.96 |
|  | Fe^3+^ 2*p*_1/2_ | 726.61 | 725.68 | 727.16 |
| Mn | Mn^0^ 2*p*_3/2_ | 638.28 | / | / |
|  | Mn^2+^ 2*p*_3/2_ | 641.67 | 641.49 | 641.60 |
|  | *Sat.* | 645.75 | 646.19 | 645.71 |
|  | Mn^0^ 2*p*_1/2_ | 649.35 | / | / |
|  | Mn^2+^ 2*p*_1/2_ | 653.14 | 653.55 | 653.78 |
| Mo | Mo^0^ 3*d*_5/2_ | 228.42 | 228.20 | 228.42 |
|  | Mo^4+^ 3*d*_5/2_ | 229.53 | 229.39 | 229.60 |
|  | Mo^0^ 3*d*_3/2_ | 231.43 | 230.91 | 231.23 |
|  | Mo^4+^ 3*d*_3/2_ | 232.44 | 232.45 | 232.25 |
|  | Mo^6+^ 3*d*_5/2_ | 232.63 | 232.63 | 233.07 |
|  | Mo^6+^ 3*d*_3/2_ | 235.67 | 235.80 | 235.97 |
| Co | Co^0^ 2*p*_3/2_ | 778.40 | / | 778.27 |
|  | Co^3+^ 2*p*_3/2_ | 780.58 | / | 780.47 |
|  | Co^2+^ 2*p*_3/2_ | 782.76 | / | 782.99 |
|  | *Sat.* 2*p*_3/2_ | 785.45 | / | 787.33 |
|  | Co^0^ 2*p*_1/2_ | 793.38 | / | 793.40 |
|  | Co^3+^ 2*p*_1/2_ | 796.84 | / | 796.23 |
|  | Co^2+^ 2*p*_1/2_ | 800.46 | / | 798.14 |
|  | *Sat.* 2*p*_1/2_ | 803.54 | / | 802.95 |
| Ni | Ni^0^ 2*p*_3/2_ | 853.05 | 852.92 | / |
|  | Ni^3+^ 2*p*_3/2_ | 856.06 | 856.53 | / |
|  | *Sat.* 2*p*_3/2_ | 860.47 | 860.58 | / |
|  | Ni^0^ 2*p*_1/2_ | 870.38 | 870.17 | / |
|  | Ni^3+^ 2*p*_1/2_ | 873.95 | 874.09 | / |
|  | *Sat.* 2*p*_1/2_ | 878.52 | 876.01 | / |

| **Sample** | **Scattering path** | **Distance (Å)** | **C.N.** | **σ^2^ (Å^2^)** | **ΔE_0_ (eV)** | **R-factor** |
| --- | --- | --- | --- | --- | --- | --- |
| RuFeCoNiMnMo | Ru-Ru | 2.66 | 3.9 | 0.005 | -4.428 | 0.00303 |
|  | Ru-M | 2.62 | 3.9 | 0.023 | -4.428 |  |
|  | Ru-O | 1.97 | 1.3 | 0.010 | -4.428 |  |
| RuFeNiMnMo | Ru-Ru | 2.69 | 3.8 | 0.005 | -9.100 | 0.01563 |
|  | Ru-M | 2.56 | 3.8 | 0.041 | -9.100 |  |
|  | Ru-O | 2.02 | 1.3 | 0.009 | -9.100 |  |
| RuFeCoMnMo | Ru-Ru | 2.67 | 3.8 | 0.005 | -9.144 | 0.01282 |
|  | Ru-M | 2.63 | 3.8 | 0.024 | -9.144 |  |
|  | Ru-O | 1.99 | 1.3 | 0.012 | -9.144 |  |

**Table S5.** A summary of the Ru *K*-edge EXAFS fitting results for RuFeCoNiMnMo, RuFeNiMnMo, and RuFeCoMnMo HEAs.

Note: C.N.: coordination numbers; R: interatomic distance; σ^2^: Debye-Waller factors; ΔE_0_: inner potential shift; R factor: fitting goodness. The amplitude reduction factor of S_0_^2^ was obtained by the fitting of Ru foil (S_0_^2^ = 0.830). To control maximum number of fitting variables ≤ the N_idp_ (N_idp_ = 2ΔkΔR/π), the Ru-M path was used to represent the possible Ru-Fe, Ru-Co, Ru-Ni, Ru-Mn, and Ru-Mo paths.

**Table S6.** A summary of the Co *K*-edge EXAFS fitting results for RuFeCoNiMnMo and RuFeCoMnMo HEAs.

| **Sample** | **Scattering path** | **Distance (Å)** | **C.N.** | **σ^2^ (Å^2^)** | **ΔE_0_ (eV)** | **R-factor** |
| --- | --- | --- | --- | --- | --- | --- |
| RuFeCoNiMnMo | Co-Co | 2.55 | 2.87 | 0.010 | -4.204 | 0.00143 |
|  | Co-M | 2.57 | 1.43 | 0.004 | -4.204 |  |
|  | Co-O | 1.99 | 0.96 | 0.007 | -4.204 |  |
| RuFeCoMnMo | Co-Co | 2.59 | 3.32 | 0.015 | -5.712 | 0.00621 |
|  | Co-M | 2.61 | 1.68 | 0.004 | -5.712 |  |
|  | Co-O | 1.98 | 1.11 | 0.011 | -5.712 |  |

Note: C.N.: coordination numbers; R: interatomic distance; σ^2^: Debye-Waller factors; ΔE_0_: inner potential shift; R factor: fitting goodness. The amplitude reduction factor of S_0_^2^ was obtained by the fitting of Co foil (S_0_^2^ = 0.746). To control maximum number of fitting variables ≤ the N_idp_ (N_idp_ = 2ΔkΔR/π), the Co-M path was used to represent the possible Co-Ru, Co-Fe, Co-Ni, Co-Mn, and Co-Mo paths.

**Table S7.** A summary of the Ni *K*-edge EXAFS fitting results for RuFeCoNiMnMo and RuFeNiMnMo HEAs.

| **Sample** | **Scattering path** | **Distance (Å)** | **C.N.** | **σ^2^ (Å^2^)** | **ΔE_0_ (eV)** | **R-factor** |
| --- | --- | --- | --- | --- | --- | --- |
| RuFeCoNiMnMo | Ni-Ni | 2.51 | 4.03 | 0.005 | 7.866 | 0.00083 |
|  | Ni-M | 2.61 | 6.23 | 0.011 | -4.015 |  |
|  | Ni-O | 2.12 | 2.02 | 0.013 | 7.866 |  |
| RuFeNiMnMo | Ni-Ni | 2.47 | 4.30 | 0.006 | -9.127 | 0.00315 |
|  | Ni-M | 2.77 | 2.86 | 0.018 | -9.127 |  |
|  | Ni-O | 1.97 | 2.15 | 0.029 | -9.127 |  |

Note: C.N.: coordination numbers; R: interatomic distance; σ^2^: Debye-Waller factors; ΔE_0_: inner potential shift; R factor: fitting goodness. The amplitude reduction factor of S_0_^2^ was obtained by the fitting of Ni foil (S_0_^2^ = 0.741). To control maximum number of fitting variables ≤ the N_idp_ (N_idp_ = 2ΔkΔR/π), the Ni-M path was used to represent the possible Ni-Ru, Ni-Fe, Ni-Co, Ni-Mn, and Ni-Mo paths.

**Table S8.** The concentration of metal ions dissolved in the electrolyte after the electrolysis, which is detected by ICP-OES.

| **Element** | **RuFeCoNiMnMo** | | **RuFeNiMnMo** | | **RuFeCoMnMo** | |
| --- | --- | --- | --- | --- | --- | --- |
|  | Con.  (mg L^-1^) | Std. Dev. | Con.  (mg L^-1^) | Std. Dev. | Con.  (mg L^-1^) | Std. Dev. |
| Ru | -0.0000 | 0.00092 | -0.0018 | 0.00050 | -0.0022 | 0.00039 |
| Fe | 0.0047 | 0.00041 | 0.0015 | 0.00004 | 0.0017 | 0.00020 |
| Co | -0.0012 | 0.00012 | / | / | -0.0009 | 0.00016 |
| Ni | -0.0096 | 0.00008 | -0.0104 | 0.00040 | / | / |
| Mn | -0.0006 | 0.00003 | 0.0001 | 0.00011 | -0.0001 | 0.00005 |
| Mo | 0.0176 | 0.00133 | 0.0024 | 0.00031 | 0.0016 | 0.00062 |

**Table S9.** Comparison of NO_3_RR performances between HEAs in this work and previously reported high-entropy/multicomponent electrocatalysts.

| **Catalysts** | **Electrolytes** | **Potentials**  **(V vs. RHE)** | **NH_3_ FE**  **(%)** | **NH_3_ yield rates**  **(mg h^-1^ mg_cat_^-1^)** | **Refs.** |
| --- | --- | --- | --- | --- | --- |
| RuFeCoNiMnMo HEAs | 0.5 M K_2_SO_4_ with 0.1 M KNO_3_ | −0.60 | 99.3 | 83.35 | This work |
| RuFeNiMnMo HEAs | 0.5 M K_2_SO_4_ with 0.1 M KNO_3_ | −0.60 | 88.6 | 31.09 | This work |
| RuFeCoMnMo HEAs | 0.5 M K_2_SO_4_ with 0.1 M KNO_3_ | −0.60 | 97.2 | 50.48 | This work |
| FL-Ag/HEA/CNFs | 0.5 M K_2_SO_4_ with 0.1 M KNO_3_ | – 0.57 | 92.7 | 41.65 | ^[6]^ |
| MnFeCoNiCu HEA | 0.5 M Na_2_SO_4_ with 0.1 M NO_3_^−^ | −0.60 | 94.5 ± 4.3 | 10.2 ± 0.5 | ^[7]^ |
| Fe-HESA NCs | 0.5 M Na_2_SO_4_ with 100 mM NaNO_3_ | −0.60 | 93.4 | 81.4 | ^[8]^ |
| LSNCMFC PNTs | 0.5 M K_2_SO_4_ with KNO_3_–N (500 mg L^–1^) | −0.9 | almost 100 (−0.6 to −0.8 V_RHE_) | 1.6575 | ^[9]^ |
| Mg_0.2_Co_0.2_Ni_0.2_Cu_0.2_Zn_0.2_O | 1 M KOH with 0.1 M KNO_3_ | −0.35 | 93.4 | 4.84 | ^[10]^ |
| A^5^Fe_2_O_4_ | 1 M KOH with 0.1 M KNO_3_  (pH = 14) | −0.50 | 98.1 | 35.7 | ^[11]^ |
| Co_1_Ni_2_Cu_1_Mn_1_Fe_1_ | 0.1 M KOH with 0.05 M KNO_3_ | −0.60 | 92 | 6.5 | ^[12]^ |
| LNPEEC NTs | 0.5 m K_2_SO_4_ with 500 mg L^-1^-N KNO_3_ | −1.00 | almost 100  (-0.7 V_RHE_) | 1.378 | ^[13]^ |
| PdMoCu trimetallene | 1 M KOH with 0.1 M KNO_3_ | −0.60 | 56.95 | 21.284 | ^[14]^ |
| Pt_0.8_Fe_0.2_Co_0.2_Ni_0.2_Cu_0.2_ HEI | 0.5 M Na_2_SO_4_ with 0.1 M NaNO_3_ | −0.90 | 88.44 | 64.4 | ^[15]^ |
| RuFeCoNiMnMo HEAs | 0.5 M K_2_SO_4_ with 0.1 M KNO_3_ | −0.60 | 99.3 | 16.67 (mg h^-1^ cm^-2^) | This work |
| FeCoNiAlTi (n-HEA) | 0.2 M K_2_SO_4_ with 50 mM KNO_3_ | −0.50 | 95.23 | 0.52 (mg h^-1^ cm^-2^) | ^[16]^ |
| Ru-MEA | 0.5 M Na_2_SO_4_ with 50 mM NaNO_3_ (pH 2.5) | −1.13 | 79.8 ± 5.8 | 3.171 (mg h^-1^ cm^-2^) | ^[17]^ |
| CuNiCoZnMn | 0.5 M Na_2_SO_4_ with 0.1 M KNO_3_ | −0.35 | 96.62 | 12.3029 (mg h^-1^ cm^-2^) | ^[18]^ |

Note: All the data were directly obtained or converted from the contents and figures presented in the relevant reported works.

**Table S10.** Comparison of NO_3_RR performances between RuFeCoNiMnMo HEAs in this work and previously reported Ru-containing electrocatalysts.

| **Catalysts** | **Electrolytes** | **Potentials**  **(V vs. RHE)** | **NH_3_ FE (%)** | **NH_3_ yield rates** | **Refs.** |
| --- | --- | --- | --- | --- | --- |
| RuFeCoNiMnMo HEAs | 0.5 M K_2_SO_4_ with 0.1 M KNO_3_ | −0.60 | 99.3 | 83.35 mg h^-1^ mg_cat_^-1^  (16.67 mg h^-1^ cm^-2^) | This work |
| Ru_15_Co_85_ HNDs | 0.1 M KNO_3_ with 0.1 M KOH | +0 | 97 ± 5 | 54.57 mg h^−1^ mg_cat_^−1^ | ^[19]^ |
| RuCu DAs/NGA | 0.1 M KNO_3_ with 0.1 M KOH | −0.40 | 95.7 | 3.10 mg h^−1^ cm^−2^ | ^[20]^ |
| Ru/MSN-WO_3−x_ | 0.1 M KNO_3_/0.25 M K_2_SO_4_/0.5 M phosphate | −0.60 | 82.4 | 12.38 mg h^−1^ cm^−2^ | ^[21]^ |
| Ru_20_Ni_80_ | 0.5 M K_2_SO_4_ with 0.1 M KNO_3_ | −0.65 | 80.8 | 8.65 mg h^−1^ cm^−2^ | ^[22]^ |
| Ru_SA_@Cu_2+1_O | 0.1 M KOH with 0.1 M KNO_3_ | −0.40 | 98.02 | 13.77 mg h^−1^ cm^−2^ | ^[23]^ |
| 10Ru/Co_3_O_4_ | 0.1 M KOH with 0.1 M KNO_3_ | −1.00 | 97.5 | 6.0 mg h^−1^ cm^−2^ | ^[24]^ |
| Ru/Cu@NOMC | 1 M KOH with 0.1 M NaNO_3_ | −0.50 | 91 | 21.54 mg h^–1^ mg_cat_^–1^ | ^[25]^ |
| Ru_5_Mo_5_-NC | 0.5 M K_2_SO_4_ with 0.1 M KNO_3_ | −0.40 | 98.3 | 1.30 mg h^–1^ mg_cat_^–1^ | ^[26]^ |
| FeCoNiCuRu | 0.1 M KNO_3_ with 0.5 M K_2_SO_4_ | −0.50 | 94.5 | 6.74 mg h^−1^ cm^−2^ | ^[27]^ |
| RuFe NFs | 0.5 M Na_2_SO_4_ with 0.1 M NaNO_3_ | −0.65 | 92.9 (−0.3 V) | 38.68 mg h^−1^ mg_cat_^−1^ | ^[28]^ |
| O-Cu-SRO | 0.1 M KNO_3_ with 0.1 M KOH | −0.90 | 95.4 | 7.20 mg h^−1^ mg_cat_^−1^ | ^[29]^ |
| Ru-Fe_2_O_3_ | 0.5 M Na_2_SO_4_ with 0.1 M NaNO_3_ | −0.90 | 72.8 | 5.59 mg h^−1^ cm^−2^ | ^[30]^ |

Note: All the data were directly obtained or converted from the contents and figures presented in the relevant reported works.

**References**

[1] S. J. Clark, M. D. Segall, C. J. Pickard, P. J. Hasnip, M. I. Probert, K. Refson, M. C. Payne, *Z. fur Krist. - Cryst. Mater.* **2005**, *220*, 567-570.

[2] J. P. Perdew, K. Burke, M. Ernzerhof, *Phys. Rev. Lett.* **1996**, *77*, 3865.

[3] P. Hasnip, C. Pickard, *Comput. Phys. Commun.* **2006**, *174*, 24-29.

[4] J. P. Perdew, J. A. Chevary, S. H. Vosko, K. A. Jackson, M. R. Pederson, D. J. Singh, C. Fiolhais, *Phys. Rev. B* **1992**, *46*, 6671.

[5] J. D. Head, M. C. Zerner, *Chem. Phys. Lett.* **1985**, *122*, 264-270.

[6] J. Hao, T. Wang, R. Yu, J. Cai, G. Gao, Z. Zhuang, Q. Kang, S. Lu, Z. Liu, J. Wu, G. Wu, M. Du, D. Wang, H. Zhu, *Nat. Commun.* **2024**, *15*, 9020.

[7] D. Yin, B. Li, B. Gao, M. Chen, D. Chen, Y. Meng, S. Zhang, C. Zhang, Q. Quan, L. Chen, C. Yang, C.-Y. Wong, J. C. Y. Ho, *Adv. Mater.* **2025**, *37*, 2415739.

[8] S. Tang, M. Xie, S. Yu, X. Zhan, R. Wei, M. Wang, W. Guan, B. Zhang, Y. Wang, H. Zhou, G. Zheng, Y. Liu, J. H. Warner, G. Yu, *Nat. Commun.* **2024**, *15*, 6932.

[9] Y. Chen, C. Chen, W.-H. Huang, C.-W. Pao, C.-C. Chang, T. Mao, J. Wang, H. Fu, F. Lai, N. Zhang, T. Liu, *ACS Nano* **2024**, *18*, 20530-20540.

[10] S. Sun, C. Dai, P. Zhao, S. Xi, Y. Ren, H. R. Tan, P. C. Lim, M. Lin, C. Diao, D. Zhang, C. Wu, A. Yu, J. C. J. Koh, W. Y. Lieu, D. H. L. Seng, L. Sun, Y. Li, T. L. Tan, J. Zhang, Z. J. Xu, Z. W. Seh, *Nat. Commun.* **2024**, *15*, 260.

[11] S. Qi, Z. Lei, Q. Huo, J. Zhao, T. Huang, N. Meng, J. Liao, J. Yi, C. Shang, X. Zhang, H. Yang, Q. Hu, C. He, *Adv. Mater.* **2024**, *36*, 2403958.

[12] W. Qiu, Y. Guo, X.-Z. Fu, J.-L. Luo, *Adv. Funct. Mater.* **2025**, *35*, 2415970.

[13] C. Chen, Z. Xu, G. Hai, W.-H. Huang, C.-W. Pao, H. Li, K. Jiang, N. Zhang, T. Liu, *Small* **2025**, *21*, 2407964.

[14] X. Tong, Z. Zhang, Z. Fang, J. Guo, Y. Zheng, X. Liang, R. Liu, L. Zhang, W. Chen, *J. Phys. Chem. C* **2023**, *127*, 5262-5270.

[15] G. Zhu, W. Bao, M. Xie, C. Qi, F. Xu, Y. Jiang, B. Chen, Y. Fan, B. Liu, L. Wang, W. Jiang, P. Qiu, W. Luo, *Adv. Mater.* **2025**, *37*, 2413560.

[16] R. Zhang, Y. Zhang, B. Xiao, S. Zhang, Y. Wang, H. Cui, C. Li, Y. Hou, Y. Guo, T. Yang, J. Fan, C. Zhi, *Angew. Chem. Int. Ed.* **2024**, *63*, e202407589.

[17] M. Yang, B. Li, S. Li, Q. Dong, Z. Huang, S. Zheng, Y. Fang, G. Zhou, X. Chen, X. Zhu, T. Li, M. Chi, G. Wang, L. Hu, Z. J. Ren, *Nano Lett.* **2023**, *23*, 7733-7742.

[18] K. Zhang, Z. Zhang, T. Yang, S. Wang, S. Liu, Z. Zhao, S. Hu, Z. Ma, J. Huang, Y. Yang, Y. Chen, B. Ge, *ACS Appl. Mater. Interfaces* **2024**, *16*, 43526-43534.

[19] S. Han, H. Li, T. Li, F. Chen, R. Yang, Y. Yu, B. Zhang, *Nat. Catal.* **2023**, *6*, 402-414.

[20] K. Liu, Z. Sun, X. Peng, X. Liu, X. Zhang, B. Zhou, K. Yu, Z. Chen, Q. Zhou, F. Zhang, Y. Wang, X. Gao, W. Chen, P. Chen, *Nat. Commun.* **2025**, *16*, 2167.

[21] H. Fu, S. Lu, Y. Xin, S. Xiao, L. Chen, Y. Li, K. Shen, *Energy Environ. Sci.* **2025**, *18*, 818-830.

[22] Y. Xiong, M. Sun, S. Wang, Y. Wang, J. Zhou, F. Hao, F. Liu, Y. Yan, X. Meng, L. Guo, Y. Liu, S. Chu, Q. Zhang, B. Huang, Z. Fan, *Adv. Funct. Mater.* **2025**, *35*, 2420153.

[23] Y. Lin, Y. Wang, Y. Xu, H. Liu, X. Liu, L. Shan, C. Wu, L. Yang, L. Song, *Adv. Funct. Mater.* **2025**, *35*, 2417486.

[24] M. Lim, Z. Ma, G. O'Connell, J. A. Yuwono, P. Kumar, R. Jalili, R. Amal, R. Daiyan, E. C. Lovell, *Small* **2024**, *20*, 2401333.

[25] J.-J. Zhang, Y.-Y. Lou, Z. Wu, X. J. Huang, S.-G. Sun, *J. Am. Chem. Soc.* **2024**, *146*, 24966-24977.

[26] X. Ge, R. Pan, H. Xie, S. Hu, J. Yuan, *Nano Lett.* **2024**, *24*, 12218-12225.

[27] T. Yang, J. Huang, S. Hu, Z. Zhang, Z. Ma, S. Liu, Y. Liu, B. Ge, P. K. Shen, S. Gao, *Chem. Eng. J.* **2024**, *500*, 157426.

[28] Y. Wang, M. Sun, J. Zhou, Y. Xiong, Q. Zhang, C. Ye, X. Wang, P. Lu, T. Feng, F. Hao, F. Liu, J. Wang, Y. Ma, J. Yin, S. Chu, L. Gu, B. Huang, Z. Fan, *Proc. Natl. Acad. Sci. U.S.A.* **2023**, *120*, e2306461120.

[29] L. Lv, H. Tan, Y. Liu, N. Li, Q. Ji, Y. Kong, H. Wang, M. Sun, M. Fan, C. Wang, W. Yan, *Adv. Funct. Mater.* **2025**, *35*, 2423612.

[30] S. Luo, H. Guo, T. Li, H. Wu, F. Zhang, C. Tang, G. Chen, G. Yang, Y. Zhou, *Appl. Catal. B Environ.* **2024**, *351*, 123967.
